
JOURNAL INFORMATION
==============================
NLM Title Abbreviation: Innov Surg Sci
Journal ID: Innov Surg Sci
NLM Journal ID: 101708165
Journal ID: iss

Innovative Surgical Sciences

EISSN: 2364-7485
Publisher: De Gruyter

ARTICLE INFORMATION
==============================
PMCID: PMC11005952
DOI: 10.1515/iss-2024-2008
Article ID: iss-2024-2008
Article version: 1
Subjects: Article

Abstracts Surgical Research

Electronic publication date: 2024 Apr 9
Publication date: 2024 Apr
Volume: 9
Issue: Suppl1
First page: 303
Last page: 385
Copyright: © 2024 by Walter de Gruyter Berlin/Boston
Copyright year: 2024
Copyright holder: De Gruyter
License: This work is licensed under the Creative Commons Attribution 4.0 International License.
License URL: https://creativecommons.org/licenses/by/4.0/

Page count: 83

==============================

Abstracts – DCK 2024 – 141st Congress of the German Society of Surgery – Leipzig, April 24–26 • DOI 10.1515/iss-2024-2008
Innov Surg Sci 2024; 9 (Special Suppl 1): s303–s385

**Advancing patient involvement in clinical research - The Patient Advisory Board of the Study Center of the German Society of Surgery**
ID: 221

**SCF - Clinical Studies**

Magdalena Holze[1], Sophia Vogel-Adigozalov[2], Maximilian Joos[2], Solveig Tenckhoff[2], Rosa Klotz[1]

[1]*Universitätsklinikum Heidelberg, Klinik für Allgemein-, Viszeral- und Transplantationschirurgie, Heidelberg, Deutschland*, [2]*Universitätsklinikum Heidelberg, Studienzentrum der Deutschen Gesellschaft für Chirurgie, Heidelberg, Deutschland*

**Background:**
The perspective of patients has historically not been heard in both clinical research and care. Currently, just few scientific institutions provide sustainable mechanisms for patient involvement in research projects. Nevertheless, patient involvement has gained importance and is now advocated not solely by patient representatives and researchers, but also demanded by funders. To meet these requirements and to strengthen active patient involvement the Patient Advisory Board (PAB) of the Study Center of the German Society of Surgery (SDGC) was founded at Heidelberg University Hospital in 2021.

**Materials and methods:**
The PAB comprises 16 members including patients undergoing major surgery, their relatives, and representatives of patient support groups, such as ILCO, and AdP. The PAB has developed and adopted its own bylaws and meets regularly. PAB activities include prioritizing research topics, advising on grant applications, generating trial documents and publications in lay terms, and involvement in study design and study-related committees.

**Results:**
As of September 2023, the PAB has conducted a total of 17 meetings and advised 20 study ideas from researchers nationwide for funding applications. 6 PAB representatives have been integrated into trial committees, contributing actively to the conduct of these trials. Besides, 2 own BMBF-funded projects were initiated in cooperation with the PAB: a systematic review examining the current status of patient involvement in surgical trials and a conceptional phase aimed at designing a multicenter RCT on prehabilitation in colorectal cancer patients.

**Conclusion:**
Institutions like the PAB play a pivotal role in advancing patient involvement, enabling researchers to actively incorporate patient experience.
The PAB is one of the first patient advisory boards in Germany. Its high level of activity and advocacy by patients, researchers and funders demonstrates the need for such institutions within the German research landscape. In addition to the general work of the PAB, first results of the conceptual phase of the PREHAB trial will be presented at the congress.

Open Access. © 2024 The Author(s), published by De Gruyter. This work is licensed under the Creative Commons Attribution 4.0 International License.

Abstracts – DCK 2024 – 141st Congress of the German Society of Surgery – Leipzig, April 24–26 • DOI 10.1515/iss-2024-2008
Innov Surg Sci 2024; 9 (Special Suppl 1): s303–s385

**Prospective, monocentric observational study on the clinical use and patient satisfaction of pfmmedical implantable vascular access ports**
ID: 553

**SCF - Clinical Studies**

Alexander Werba[1], Frank Pianka[1], Fabian Schuh[1], Julia Raba[1], Roland Hennes[1], Phillip Knebel[1], Rosa Klotz[1]

[1]*Chirurgische Klinik, Heidelberg, Deutschland*

**Background:**
A long-term central venous port catheter is an indispensable tool mainly used for the application of chemotherapy or parenteral nutrition in various benign and malignant diseases. Patient reported outcome and experience measures gain importance in the joint therapy decision between physicians and particularly chronically ill patients. The aim of this study was to evaluate patient satisfaction and postoperative complications in patients undergoing implantation of a specific port system compared to available literature.

**Materials and methods:**
A prospective, monocentric observational study was conducted. Adult patients with any indication for a venous access port were included. Based on available studies, a sample size of 150 patients was needed to prove non-inferiority for the primary endpoint patient satisfaction compared to previous studies assessed via a validated 15 questions questionnaire. The score ranged from 0 (best value) to 100 (worst value). The endpoint was assessed via an unpaired t-test for non-inferiority in the per-protocol analysis set. Besides, patient diaries were used to document frequency and type of use of the port and complications were documented. The follow-up included study visits at 4 weeks and 6 months after implantation.

**Results:**
Between April 13, 2022, and August 17, 2022, 149 patients (68 male (45.64%) and 81 female (54.36%) patients) with a mean age of 58.8 (IQR 13.0) years were included. All patients suffered from an oncological disease. 117 (78.52%) ports were implanted using the Cut-Down-Technique, 5 (3.36%) using seldinger-technique and 27 (18.12%) a combination of both techniques. Venous access was established using the cephalic vein in 126 (84.56%) cases. Mainly, “Jet Port Plus II” (81, 54.36%) and “T-Port Contrast” (62, 41,61%) systems were used. Overall, 20 patients died during the follow-up of 6 months and in two patients the port system had to be removed due to infection. None of the events were directly related to the implanted port system. According to patient diaries, there were 1240 accesses of the implanted catheter, of which 867 (79,83%) proceeded without complications or multiple punctures. Patient satisfaction surpassed the reference value of the comparison literature in all four analyzed domains based on the validated questionnaire (‘satisfied overall’ 14.9 ± 18.9 vs. 23.6, $p<0.0001$; ‘choose port again’ 11.6 ± 21.1 vs. 17.4, $p<0.0001$; ‘cosmetic result’ 32.9 ± 26.8 vs. 27.9, $p=0.0034$; ‘pain’ 13.1 ± 21.0 vs. 19.5, $p<0.0001$). Statistically, it is permissible that in the case where non-inferiority has been proven also a test for superiority can be performed, however superiority could not be shown.

**Conclusion:**
When evaluating the risks and benefits of a particular therapy, patient reported outcome measures are essential. Patients undergoing implantation of vascular access ports report adequate postoperative satisfaction in comparison to previous studies. This study was sponsored by ‘pfm medical ag’.

**B7-1 and PlGF-1 are two possible new markers to identify trauma patients at risk to develop post-surgical complications**
ID: 662

**SCF - Clinical Studies**

Andreas Nüssler[1], Regina Breinbauer[2], Sabrina Ehnert[1], Marie Karolina Reumann[1], Mika Rollmann[1], Tina Histing[1]

[1]*University Tübingen, Dept. of Traumatology and Reconstructive Surgery, Siegfried Weller Institute, Tübingen, Deutschland*, [2]*Tübingen, University Tübingen, Siegfried Weller Institute, Tübingen, Deutschland*

**Background:**
Around 10% of fractures lead to complications. With increasing fracture incidences in recent years, this poses a serious burden on the healthcare system, with increasing costs for treatment. In the present study, we aimed to identify potential 'new' blood markers to predict the development of post-surgical complications in trauma patients following a fracture.

**Materials and methods:**
292 (244 for blood analysis) trauma patients with a complete three-months follow-up were included in this study. 2 complication groups were distinguished based on the Clavien-Dindo (CD) classification: CD grade I and CD grade III groups were compared to the controls (CD 0). The patients' data were analyzed using JMP 16.2 software (SAS Institute Inc., Cary, NC, USA), to identify potential risk factors based on the questionnaire responses. Subsequently, a partitioning analysis was conducted to distinguish between the control group (with no complications) and the two distinct complication groups. Based on the identified risk factors, a meticulous personal matching process was undertaken to minimize the influence of any other variables that could potentially contribute to complications. Human Cytokine Antibody Arrays C5 and C1 were chosen to screen different factors affecting the immune system. In addition, Human Angiogenesis Antibody Array C2 and Human TGF beta Array C2 were selected to screen angiogenic factors changed in sera. All arrays (BioCat, Heidelberg, Germany) were performed following the manufacturer's protocol. Based on array analyses specific Enzyme-Linked-Immunosorbent-Assays (ELISA) were performed to verify array analyses.

**Results:**
Analysis of the patients' data revealed that risk factors are dependent on sex. For men (CD 0: 133; CD I: 12; CD III 18 patients) alcohol consumption was significantly increased for CD I and CD III compared to control with $p = 0.009$ and $p = 0.007$, respectively. For women (CD 0: 107; CD I: 10; CD III 12 patients) a significantly increased average BMI [kg/$m^2$] from 25.5 to 29.7 with CD III was observed, as well as an elevation from one to three comorbidities ($p = 0.003$). Both men and women developing a CD III complication showed elevated blood levels of B7-1 ($p = 0.015$ and $p = 0.018$, respectively) and PlGF-1 ($p = 0.009$ and $p = 0.031$, respectively), with B7-1 having a better sensitivity.

**Conclusion:**
In this study, we identified two potential new markers, B7-1 and PlGF-1, that hold promise for predicting future complication development in trauma patients. Our study revealed gender differences in cytokine profiles to identify patients at risk in trauma patients. Nevertheless, it is important to emphasize that further studies are needed to evaluate the effectiveness of B7-1 and PlGF-1 in predicting complications in trauma patients.

Abstracts – DCK 2024 – 141st Congress of the German Society of Surgery – Leipzig, April 24–26 • DOI 10.1515/iss-2024-2008
Innov Surg Sci 2024; 9 (Special Suppl 1): s303–s385

**Pharmacokinetics and Pharmacodynamics of Therapeutic Rivaroxaban in Bariatric Patients**
ID: 723

**SCF - Clinical Studies**

Jonas Fischer[1], Guido Stirnimann[1], Philipp Nett[1], Yves Borbély[1], Sabine Schädelin[2], Debora Bertaggia[3], Daniel Candinas[1], Dino Kröll[1]

[1]*Inselspital, Universitätsspital Bern, UVCM, Bern, Schweiz*, [2]*Clinical Trial Unit, Department of Clinical Research, Basel, Schweiz*, [3]*Division of Haematology and Central Haematology Laboratory, University of Lausanne, Lausanne, Schweiz*

**Background:**
Thromboembolic events after bariatric surgery are serious complications. Limited knowledge exists about the use of direct oral anticoagulants (DOACs) in these patients. Therefore, investigating the pharmacokinetics (PK) and pharmacodynamics (PD) of Rivaroxaban in this population is crucial.

**Materials and methods:**
In this phase I monocentric study, bariatric patients were administered single oral doses of Rivaroxaban (20 mg once daily) before, at 3 and 30 days, and 3 months after undergoing sleeve gastrectomy (SG) or Roux-en-Y-gastric bypass (RYGB). PK and PD parameters were evaluated by collecting data at specific time points within a 24-hour period.

**Results:**
We included 9 patients (age 49.9 ± 14.4y; 78% female) with a mean BMI of 46.8 ± 9 kg/m$^2$. 7 SG and 2 RYGB patients were included. Rivaroxaban mean areas under the curve (AUC) 3 days, 30 days, and 3 months after the bariatric procedure were (2474.7 µg*h/L, CV 1.4., 2468.2 µg*h/L, CV 1.7, 3266.4.2 µg*h/L, CV 1.2) comparable to those measured preoperatively (2441.8 µg*h/L, CV 1.4). The bioavailability of Rivaroxaban measured by parameters $C_{max}$ and AUC showed no significant difference between the pre- and postoperative periods.

**Conclusion:**
Rivaroxaban exhibited consistent PD and PK values in the early postoperative bariatric period similar to the preoperative values. In particular, early begin with Rivaroxaban (e.g. 3 days postoperatively) is considered feasible and safe. Both surgical procedures (RYGB vs. SG) did not affect the PD and PK values.

**Organization, function and gene expression of tertiary lymphoid structures in pancreatic cancer resembles lymphoid follicles in secondary lymphoid organs and their abundance is related to superior survival**
ID: 58

**SCF - Immunotherapy**

Jonas Lehmann[1], Martin Thelen[1], Simon Schran[1], Maria A. Garcia-Marquez[1], Christoph Kreer[1], Stephan Kruger[2], Stefan Böck[2], Steffen Ormanns[3], Martina Rudelius[3], Felix Popp[4], Florian Klein[1 5 6], Michael von Bergwelt-Baildon[1 2 7], Christiane Bruns[1 4], Alexander Quaas[8], Kerstin Wennhold[1], Hans Schlößer[1 4]

[1]*Center for Molecular Medicine Cologne (CMMC), Köln, Deutschland*, [2]*Ludwig-Maximilians-Universität München, Klinik für Innere Medizin III, München, Deutschland*, [3]*Ludwig-Maximilians-Universität München, Institut für Pathologie, München, Deutschland*, [4]*Uniklinik Köln, Klinik und Poliklinik für Allgemein-, Viszeral- und Tumorchirurgie, Köln, Deutschland*, [5]*Uniklinik Köln, Laboratory of Experimental Immunology, Institute of Virology, Köln, Deutschland*, [6]*Deutsches Zentrum für Infektionsforschung (DZIF) e. V., Braunschweig, Deutschland*, [7]*Deutsches Krebskonsortium (DKTK)*, [8]*Uniklinik Köln, Institut für Pathologie, Köln, Deutschland*

**Background:**
Tertiary lymphoid structures (TLS) have been described in close proximity to tumor areas in a variety of cancer types, where they are assumed to represent hotspots for T cell and B-cell activation leading to tumor-specific immune responses. Thus, abundance of TLS is related to cancer-specific survival and susceptibility to immune checkpoint inhibition.

**Materials and methods:**
To identify shared and TLS/secondary lymphoid organ (SLO)-specific features we performed immunohistochemistry staining and 5-color immunofluorescence (IF) of previously untreated pancreatic ductal adenocarcinoma (PDAC) samples. NanoString-based RNA expression analysis of laser microdissected tissue was conducted for 12 patients to compare gene expression in TLS, PDAC, SLOs and normal pancreatic tissue. B-cell receptor sequencing was performed to determine clonal expansion.

**Results:**
PDAC patients with a high abundance of TLS inside and surrounding the tumor showed a higher overall survival and TLS abundance correlated with infiltration of T cells in the tumor microenvironment. IF revealed structural homologies between TLS and SLO with a similar distribution of B cells, T cells, follicular dendritic cells and lymphatic vessels. In addition, we found largely overlapping expression patterns of a variety of immune related gene clusters. However, especially expression levels of T-cell and complement-associated genes were distinct. Most TLS expressed Ki-67, Pax5, AID and IgG, proving proliferation, class switching and affinity maturation of B cells in close proximity to the tumor. B-cell receptor sequencing of microdissected TLS and SLO, identified an overlap of expanded sequences.

**Conclusion:**
Our analyses of organization, function and gene expression patterns revealed a high overlap between SLOs and TLS in PDAC. These results indicate a role of TLS in cancer immunosurveillance of PDAC, which may be susceptible to therapeutic targeting in this highly aggressive and immunotherapy-resistant disease.

**Functional role of tumor-specific B cells and tertiary-lymphoid structures in preclinical models of immune checkpoint therapy**
ID: 59

**SCF - Immunotherapy**

Jonas Lehmann[1], Martin Thelen[1], Maria Garcia-Marquez[1], Pauline Volkmar[1], Klara Siepmann[1], Therese Seidlitz[2], Daniel Stange[2], Philipp Fervers[3], Thorsten Persigehl[3], Christiane Bruns[1 4], Michael von Bergwelt-Baildon[1 5], Kerstin Wennhold[1], Hans Schlößer[1 4]

[1]*Center for Molecular Medicine Cologne (CMMC), Köln, Deutschland*, [2]*Universitätsklinikum Carl Gustav Carus Dresden, Dresden, Deutschland*, [3]*Uniklinik Köln, Institut für Radiologie, Köln, Deutschland*, [4]*Uniklinik Köln, Klinik und Poliklinik für Allgemein-, Viszeral- und Tumorchirurgie, Köln, Deutschland*, [5]*Ludwig-Maximilians-Universität München, Klinik für Innere Medizin III, München, Deutschland*

**Background:**
Immune checkpoint inhibition (CKI) demonstrated breakthrough therapeutic efficacy in a variety of cancer types. Recent data suggest an important role of B cells and tertiary-lymphoid structures for susceptibility of cancer patients to CKI.

**Materials and methods:**
To advance our understanding of B cell specific mechanisms in the setting of CKI, we used two genetically modified mouse models, which express a tamoxifen-inducible Cre-recombinase in the stomach-specific Anxa10 locus. The genomically stable (GS) cancer model ($Cdh1^{fl/fl}$, $Kras^{G12D/+}$ and $Smad4^{fl/fl}$) demonstrates a slow progressing tumor growth as observed by magnetic resonance imaging (MRI). The chromosomally instable (CIN) model ($Kras^{G12D/+}$, $Tp53^{R172H/+}$ and $Smad4^{fl/f}$) shows a more rapid, intestinal tumor growth. For study of antigen-specific immune responses, we included the subcutaneous PancO2-OVA tumor model. All three mouse models received single or combination CKI with anti-PD-1 and anti-4-1BB.

**Results:**
CKI in the PancO2-OVA model resulted in a significant increase of OVA-specific B cells and secretion of OVA-specific antibodies. This was accompanied by an increase of T follicular helper cells and activated $CD86^+$ B cells. In the genetically modified cancer models, tumor growth was accompanied by development of metastases in liver, lymph nodes and lungs at later stages. We detected B-cell clusters in the tumor microenvironment indicating the presence of tertiary-lymphoid structures. In the GS model, combination therapy with anti-PD1 and anti-4-1BB did not result in a significant reduction of tumor growth. However, we observed increased percentages of memory B cells, germinal center B cells, activated $CD86^+$ B cells and activated T-cell subpopulations.

**Conclusion:**
TLS are usually not observed in cancer mouse models, thus the here described gastric cancer models offer a unique opportunity to study TLS formation. CKI seems to increase the anti-tumor B-cell response by enhanced antigen presentation and humoral-immune response. Our results underline the role of B cells in CKI.

Abstracts – DCK 2024 – 141st Congress of the German Society of Surgery – Leipzig, April 24–26 • DOI 10.1515/iss-2024-2008
Innov Surg Sci 2024; 9 (Special Suppl 1): s303–s385

**Navigating the Tumor Landscape: Tumor Associated Macrophages Drive Pro-Tumorous Microenvironment through CCL17-CCR4 and CCL2-CCR2 axes Mediated Monocyte Recruitment in Pancreatic cancer**
ID: 467

**SCF - Immunotherapy**

Aydar Khabipov[1], Florian Novak[2], Maik Lenz[1], Tobias Schulze[1], Lars - Ivo Partecke[2], Stephan Kersting[1]

[1]*Universitätsmedizin Greifswald, Klinik für Allgemein und Viszeralchirurgie, Greifswald, Deutschland*, [2]*Helios Klinikum Schleswig, Klinik für Allgemein- und Viszeralchirurgie, Schleswig, Deutschland*

**Background:**
Pancreatic cancer is known for its tumor microenvironment (TME), which is rich in stromal and immune cells supporting cancer growth and facilitating therapy resistance. In particular, tumor-associated macrophages (TAMs) are the most prominent immune cells in the tumor stroma and known for their angiogenesis- and metastasis-promoting properties, which contribute to the failure of conventional therapies for pancreatic cancer. However, molecular mechanisms behind macrophage recruitment and the role of TAM in the establishment of TME is not finally understood yet. In this study we investigated the influence of pancreatic cancer cells on macrophage differentiation and the impact of TAM on macrophage recruitment focusing on the CCR4-CCL17 and CCR2 –CCL2 axes.

**Materials and methods:**
Effects of pancreatic cancer cells (PCC) (cell lines: PDA6606/ Panc02) on naïve Macrophages (RAW264.7) regarding M2 differentiation was analyzed morphologically and via flow cytometry. The induction of migration related chemokine receptors CCR2/CCR4 on TAM was investigated via flow cytometry. Migration assays (newly established “modified ibidi-chamber assay” and transwell assay) were used to detect effects of TAMs and/or PCC on migration of naïve macrophages. Cytokine profiles were analyzed with multiplex ELISA. Block of CCR2- and CCR4-receptors on macrophages were blocked using antibodies to validate the hypothesis.

**Results:**
Pancreatic cancer cells induced the pro-tumorous M2 polarization and the expression of CCR2 and CCR4 in macrophages. TAM secrete CCL17 and CCL2, the ligands for CCR2 and CCR4. Migration assays showed increased recruitment of naïve macrophages to macrophage-tumor co-cultures (compared to tumor- or macrophage mono-cultures). these, the recruited naïve macrophages are polarized to (M2)-TAM and force further macrophage recruitment by releasing CCR ligands. Chemokine receptor block of CCR2/4 revealed efficient reduction of TAM-triggered macrophage recruitment.

**Conclusion:**
The interaction between pancreatic cancer cells, TAM and naïve macrophages facilitates the establishment of a pro-tumorous microenvironment in a sort of snowball effect. TAM seem to play a crucial role in recruitment of further macrophages. Hereby CCR4 –CCL17 and CCR2 –CCL2 are central axes for macrophage recruitment. Chemokine receptor block targeting CCR2 / CCR4 could inhibit re-establishment of malign TME after curative surgery.

**Tumor-derived CD4-CD8- double-negative (DN) T cells predominantly kill autologous liposarcoma tumoroids.**
ID: 839

**SCF - Immunotherapy**

Adrian Sagebiel[1], Simone Schewe[1], Jana Striefler[1], Matthias Priemel[1], Thilo Hackert[1], Nicola Gagliani[1], Anna Dupree[1]

[1]*Universitätsklinikum Hamburg-Eppendorf, Hamburg, Deutschland*

**Background:**
Sarcomas are a group of heterogeneous malignancies rising from mesenchymal tissues. Despite their rarity, a lack of sufficient systemic therapy strategies makes sarcomas a challenging and clinically relevant subject of basic scientific research. While immunotherapies are revolutionizing therapies in many solid malignancies, such approaches don’t play a relevant role in sarcoma oncology yet.
To establish potent immunotherapies like targeted checkpoint inhibition in sarcoma, a deeper understanding of the immunological tumor microenvironment is crucial. Many sarcoma tumors are considered to be immunological “cold” tumors, as the expression of neoantigens and inflammation is relatively low. With this study we aim to get a broader understanding of relevant T cells populations and mechanisms resulting in the silence of the immune system in liposarcoma.

**Materials and methods:**
Freshly processes liposarcoma tumors (n=5) were digested and processed for Single cell RNA-Sequencing. Large scale phenotypic analysis of liposarcoma samples (n=32) were performed using multiparameter flow cytometry. Isolated lymphocytes from sarcoma tissues were sorted for specific populations of interest and clonally expanded. Sarcoma cells from the freshly processed samples were taken into culture and after 2-3 propagations used to generate patient-derived three dimensional sarcoma tumoroids. Expanded lymphocytes of interest were subsequently added to their autologous tumoroids. Killing capacity as well as tumor cell growth were analyzed by apoptosis/live-dead staining as well as by using proliferation dyes like CFSE.

**Results:**
Single cell RNA-Sequencing allows for an in-depth analysis of the whole transcriptome of the immunological tumor microenvironment in liposarcoma. Findings from this unsupervised Single cell analysis were then confirmed in a larger scale using multiparameter flow cytometry. Besides the presence CD8+ T cells, the abundance of CD8-CD4- double-negative T cells stands out. Interestingly CD4+ T cells were hardly found in sarcoma tumor tissues. While CD8+ T cells were mostly presented exhausted, expressing genes like PD1 and TOX, especially the CD8-CD4- double-negative populations showed high expressions of different Granzymes and Perforin.
Besides in-depth phenotyping of these T cell populations, we designed three-dimensional patient-derived tumoroid models in which sarcoma cells were co-cultured three dimensionally together with patient-derived T cell populations of interest, to assess their biological role in the tumor microenvironment. After 7-10 days of co-culture sarcoma and T cells were harvested and analyzed by flow cytometry.
Here we showed that especially the CD8-CD4- double negative T cell population showed increased cytotoxic effects against their autologous tumoroids, compared to blood- and tumor-derived CD8+ T cells. Interestingly upon co-culture with sarcoma tumoroids a relevant fraction of the initially CD8-CD4- double-negative T cells (re)-acquired CD4-expression during the process of expansion as well as tumoroid-T cell coculture.

**Conclusion:**
These findings show that in a controlled environment, T cell populations originating from sarcoma tumors bare the potential to effectively kill and engage against sarcoma tumoroids in an autologous tumor model. Why the immunological response in the original tumors is rather low, still needs to be elucidated. Our pipeline of co-culturing patient-derived tumoroids with specific populations of interest allows for their functional assessment bedsides in-depth phenotyping from single cell sequencing.
The majority of T cells in sarcoma being CD8-CD4- double-negative with a striking absence of CD4+ T cells, implicates that sarcomas can render their tumor microenvironment to become immunosuppressive, which can be reverted in vitro via clonal expansion of specific populations of interest.

Abstracts – DCK 2024 – 141st Congress of the German Society of Surgery – Leipzig, April 24–26 • DOI 10.1515/iss-2024-2008
Innov Surg Sci 2024; 9 (Special Suppl 1): s303–s385

**Patterns of immune evasion in primary and metastatic colorectal cancer**
ID: 850

**SCF - Immunotherapy**

Elena Marie Hagen[1], Martin Thelen[1], Kerstin Wennhold[1], Maria Garcia-Marquez[1], Jonas Lehmann[1], Miriam Werner[1], Pauline Volkmar[1], Su Ir Lyu[2], Michael von Bergwelt[3 4], Georg Dieplinger[5], Christiane Bruns[5], Alexander Quaas[2], Hans Schlößer[1 5]

[1]*Center for Molecular Medicine Cologne (CMMC), Faculty of Medicine and University Hospital Cologne, Cologne, Deutschland*, [2]*Institute of Pathology , Faculty of Medicine and University Hospital Cologne, Cologne, Deutschland*, [3]*Department of Internal Medicine III, University Hospital of Ludwig Maximilians University, Munich, Deutschland*, [4]*German Cancer Consortium (DKTK), Heidelberg, Deutschland*, [5]*Department of General, Visceral, Cancer and Transplantation Surgery, Faculty of Medicine and University Hospital Cologne, Cologne, Deutschland*

**Background:**
Colorectal cancer (CRC) is one of the most common cancer entities worldwide and contributes significantly to the overall cancer-related mortality. Stage-dependent therapeutic algorithms of CRC are mainly based on surgery, chemotherapy, and radiation. The NICHE-I and NICHE-II trials demonstrated exceptional efficacy of neoadjuvant immunotherapy targeting the PD-(L)1 pathway in a subgroup of CRC patients with DNA-mismatch-repair deficiency (dMMR)/microsatellite instability (MSI). In contrast, results of large clinical trials evaluating anti-PD-(L)1 immunotherapy in DNA-mismatch-repair-proficient (pMMR)/ microsatellite-stable CRC were disappointing. As the liver is the most important metastatic site of CRC, we aim to elucidate mechanisms of resistance in the microenvironment of hepatic metastasis of CRC patients.

**Materials and methods:**
We included samples from 68 patients in our study, 28 paired primary tumor and hepatic metastases of CRC patients, 15 colorectal carcinomas and 15 hepatocellular carcinomas. Tumor-infiltrating lymphocytes (TILs), PBMCs, healthy tissue and metastases infiltrating lymphocytes were generated from patient samples. We perform flow cytometry to compare immune cell subsets in different samples. Furthermore, immunohistochemical stainings (CD3, CD8, CD20) were performed to calculate the immune score and to quantify TLS in the TME. Expression of genes related to antigen presentation will be analyzed by 3'-RNA-sequencing (out of FFPE-Material). Additionally, we will compare expression of 11 important proteins of the antigen-presenting machinery by immunohistochemistry.

**Results:**
We found several differences of the immunological infiltrate in the metastases compared with the corresponding primary tumor. Analysis of T-cell abundance in primary tumors and the corresponding metastasis revealed a lower T-cell infiltrate in metastasis. Preliminary FACS analyses show a significant decrease of $CD4^{+}$ T- fractions in metastasis infiltrating lymphocytes (MILs) compared to TILs. This reduction is not due to regulatory T cells. Furthermore, we found no differences in PD1 or PDL1 expression on T cells between TILs and MILs. We will present detailed analyses regarding expression of co-inhibitory molecules on lymphocyte subsets in the different compartments.

**Conclusion:**
First results of our study demonstrate decreased immune cell abundance in the metastatic environment, and we aim to elucidate the underlying mechanisms of immune escape. Especially combination therapies aiming to upregulate antigen presentation within metastases appear promising for combined immunotherapy approaches.

Abstracts – DCK 2024 – 141st Congress of the German Society of Surgery – Leipzig, April 24–26 • DOI 10.1515/iss-2024-2008
Innov Surg Sci 2024; 9 (Special Suppl 1): s303–s385

**IMAGINER – Improving accuracy with Augmented Reality Navigation system during placement of external ventricular drains: A feasibility study**
ID: 305

**SCF - Innovative Surgical Techniques**

Rony Grunert[1 2], Martin Vychopen[1], Fabian Kropla[1], Johannes Wach[1], Dirk Winkler[1], Erdem Güresir[1]

[1]*University of Leipzig, Department of Neurosurgery, Leipzig, Deutschland*, [2]*Medical Engineering Technologies Ltd, Dresden*

**Background:**
The placement of a ventricular catheter (i.e. external ventricular drain, EVD) is a common and essential neurosurgical procedure. In addition, this procedure is one of the first performed by young neurosurgeons. [1] With or without surgical experience, placement of an EVD according to anatomical landmarks alone can be difficult, with the potential risk of inaccurate catheter placement. Repeated corrections always imply the risk of avoidable complications [2,3]. Augmented reality (AR) could provide a useful guide and improve the accuracy of drain placement, especially in patients with acute pathology leading to displacement of anatomical structures [4,5]. We therefore performed a feasibility study using AR in EVD placement procedures performed in a human cadaver model.

**Materials and methods:**
20 medical students performed the EVD placement procedure with a Cushing's ventricular cannula on the right and left side. The cannula was placed according to landmarks on one side and with the assistance of AR (Microsoft Hololens 2) on the other side. With AR a planned trajectory was displayed in the field of view, which guides the placement of the cannula. Subsequently, the actual position of the cannula was assessed with the help of a CT with a 1mm layer thickness. The bony structure as well as the left and right cannula positions were registered to the CT with the planned target point before the placement procedure. The software Cloudcompare was applied for the registration and the evaluation of accuracy.

**Results:**
EVD placement using AR was easily performed by all medical students. The predefined target point (inside the lateral ventricle) was stuck with both techniques. However, it could be shown that the scattering radius of the target point reached with AR (12mm) could be reduced by more than 54% compared to the puncture without AR (26mm) and this represents a doubling of the puncture accuracy.

**Conclusion:**
This feasibility study specifically showed that the integration and use of augmented reality helps to achieve more than double the accuracy in the placement of ventricular catheters. Due to the easy availability of these new tools and the intuitive handling, we see great potential in AR to improve the accuracy.

**IMAGINER 2 – Improving accuracy with Augmented Reality Navigation system during placement of external ventricular drains over Kaufman´s, Keen´s, Kocher´s and Frazier's point**
ID: 308

**SCF - Innovative Surgical Techniques**

Martin Vychopen[1], Johannes Wach[1], Fabian Kropla[1], Dirk Winkler[1], Erdem Güresir[1], Rony Grunert[1 2]

[1]*University of Leipzig, Department of Neurosurgery, Leipzig, Deutschland*, [2]*Medical Engineering Technologies Ltd, Frauenhofer Institute for Machine Tools and Forming Technology, Dresden*

**Background:**
External ventricular drain (EVD) placement is a routinely performed neurosurgical procedure that plays a crucial role in a treatment of acute hydrocephalus and provides intracranial pressure monitoring. Kocher´s point is the most commonly used anatomical landmark for the procedure. [1,2,3] In several cases, other anatomical landmarks must be used in order to perform the procedure safely and successfully, which might be challenging even for an experienced neurosurgeon. In such cases, augmented reality (AR) might be the decisive factor for a successfully and safely performed procedure. The previous study (IMAGINER I) [4] presented an improved accuracy by ventriculostomy performed by medical students without previous neurosurgical experience. In this study, we evaluate the impact of augmented reality on the accuracy of EVD placement performed by experienced neurosurgeons.

**Materials and methods:**
In our study, neurosurgeons perform ventriculostomies over Kaufman´s, Keen´s, Kocher´s and Fraizer´s point on a Styrofoam head [5]. We compare ventriculostomies according to anatomical landmarks with ventriculostomies performed with the aid of the Microsoft Hololens Augmented Reality system according to a pre-planned trajectory. Subsequently, CT-Scans are performed to assess the data. The scattering radius of the target points and the individual trajectories are then compared. Furhermore, Kakarla Grading [6] is used to evaluate the catheter tip location.

**Results:**
The scattering radius of the target points with AR will be compared between both methods.
Furthermore, we will compare the scattering radius differences between neurosurgeons with +7 years of experience compared to neurosurgeons with ≤ 7 years of experience and general neurosurgeons vs. neurosurgeons with pediatric experience.
The Kakarla Grading of catheter placement scale will be used to compare the catheter position in relation to the ventricle system.

**Conclusion:**
Following the IMAGINER I results, we will perform an evaluation of the augmented reality supported placement of a ventricular drain over Kaufman´s, Keen´s, Kocher´s and Fraizer´s point performed by experienced neurosurgeons.

1) Todnem, N.; Nguyen, K.D. ; Reddy, V. ; Grogan, D. ; Waitt, T. ; Alleyne, C.H. A simple and cost-effective model for ventricular catheter placement training: technical note. *J Neurosurg.* **2020** 1;134(5):1640-1643.
2) Fried, H.I. ; Nathan, B.R. ; Rowe, A.S.; Zabramski, J.M.; Andaluz, N.; Bhimraj, A.; Guanci, M.M.; Seder, D.B.; Singh, J.M. The Insertion and Management of External Ventricular Drains: An Evidence-Based Consensus Statement : A Statement for Healthcare Professionals from the Neurocritical Care Society. *Neurocrit Care.* **2016** 24(1):61-81.
3) Maher Hulou, M.; Maglinger, B.; McLouth, C. J.; Reusche, Catalina M.; Fraser, Justin F. Freehand frontal external ventricular drain (EVD) placement. Accuracy and complications. *Journal of clinical neuroscience: official journal of the Neurosurgical Society of Australasia* **2022**, 97, S. 7–11.

4) IMAGINER I - Improving accuracy with Augmented Reality Navigation system during placement of external ventricular drains – under revision
5) Morone PJ, Dewan MC, Zuckerman SL, Tubbs RS, Singer RJ. Craniometrics and Ventricular Access: A Review of Kocher's, Kaufman's, Paine's, Menovksy's, Tubbs', Keen's, Frazier's, Dandy's, and Sanchez's Points. Oper Neurosurg (Hagerstown). 2020 May 1;18(5):461-469. doi: 10.1093/ons/opz194. PMID: 31420653.
6) Kakarla UK, Kim LJ, Chang SW, Theodore N, Spetzler RF. Safety and accuracy of bedside external ventricular drain placement. Neurosurgery. 2008 Jul;63(1 Suppl 1):ONS162-6; discussion ONS166-7. doi: 10.1227/01.neu.0000335031.23521.d0. PMID: 18728595.

Abstracts – DCK 2024 – 141st Congress of the German Society of Surgery – Leipzig, April 24–26 • DOI 10.1515/iss-2024-2008
Innov Surg Sci 2024; 9 (Special Suppl 1): s303–s385

**Minimally invasive intrathoracic negative-pressure therapy and flexiblethoracoscopy (FlexVATS) for patients with pleural empyema**
ID: 431

**SCF - Innovative Surgical Techniques**

Björn-Ole Stüben[1], Gabriel Plitzko[1], Julia Sauerbeck[2], Philipp Busch[1], Nathaniel Melling[3], Matthias Reeh[1], Jakob Izbicki[4], Thomas Rösch[5], Kai Bachmann[4], Michael Tachezy[4]

[1]*Klinik für Allgemein-, Viszeral- und Thoraxchirurgie, Universitätsklinikum Hamburg-Eppendorf, Hamburg*, [2]*Klinik für diagnostische und interventionelle Radiologie und Nuklearmedizin, Universitätsklinikum Hamburg-Eppendorf, Hamburg*, [3]*Klinik für diagnostische und interventionelle Radiologie und Nuklearmedizin, Universitätsklinikum Hamburg-Eppendorf, Hamburg, Deutschland*, [4]*Klinik für Allgemein-, Viszeral- und Thoraxchirurgie, Universitätsklinikum Hamburg-Eppendorf, Hamburg, Deutschland*, [5]*Klinik für interdisziplinäre Endoskopie, Universitätsklinikum Hamburg-Eppendorf, Hamburg, Deutschland*

**Background:**
Surgical treatment of pleural empyema is associated with considerable postoperative complications and mortality rates, and alternative treatment options are being explored to improve patient outcomes. The aim of this study was to determine whether a new surgical method using a flexible endoscope (FlexVATS) to perform sparing debridement and apply negative-pressure therapy without extensive decortication may be an alternative treatment option for empyema.

**Materials and methods:**
Seventeen consecutive patients treated with FlexVATS between February 2021 and August 2022 were included. Only patients for whom FlexVATS was the first therapeutic intervention for pleural empyema were included. Treatment success, defined as infection resolution, was the primary endpoint of the study. The secondary endpoints were length of hospital stay, 90-day mortality, and empyema cavity volume reduction. Patients who had previously been treated for pleural empyema by either drainage or surgery were excluded.

**Results:**
17 patients with pleural empyema were included in the study. The median (IQR) duration of vacuum treatment was 15 days (8–35 days). Twelve of the 17 (71%) patients were successfully treated, and a significant reduction in the empyema cavity volume was observed. 41% of the dressing changes were performed outside the operating room. Compared with a historic cohort of conventionally treated patients (decortication via VATS or thoracotomy), the 90- day mortality rates tended to be lower without reaching statistical significance. Three patients (18%) died in hospital during treatment. No negative pressure-therapy-related complications were observed.

**Conclusion:**
FlexVATS therapy is a promising alternative therapy for both healthy and debilitated patients with pleural empyema. Larger randomised trials are required to validate this treatment option.

**Biomechanical comparison of five different techniques for the stabilization of a ruptured pubic symphysis**
ID: 582

**SCF - Innovative Surgical Techniques**

Marie Keryakas[1], Dag Wulsten[1], Andreas Ellmerer[2], Alexander Trulson[3], Tina Histing[4], Steven Herath[4], Markus Küper[4]

[1]*Charité-Universitätsmedizin Berlin, Julius Wolff Institut für Biomechanik and muskuloskelettale Regeneration, Berlin, Deutschland*, [2]*Universitätsklinik Innsbruck, Klinik für Orthopädie und Traumatologie, Innsbruck, Österreich*, [3]*BG Unfallklinik Murnau, Klinik für Unfallchirurgie, Orthopädie und Allgemeinchirurgie, Murnau am Staffelsee, Deutschland*, [4]*BG Unfallklinik Tübingen, Klinik für Unfall- und Wiederherstellungschirurgie, Tübingen, Deutschland*

**Background:**
The so-called "open-book injury" (symphyseal rupture with or without sacroiliac joint (ISG) injury) is a typical injury of the pelvic ring in the young patient. Therapy of choice is to reconstruct the pelvic ring as stably as possible. The most stable treatment for the anterior pelvic ring is symphysis-bridging plate osteosynthesis. This usually requires an open surgical procedure. In contrast, there is a desire for minimally invasive approaches, as it has been shown that minimally invasive approaches lead to a better perioperative outcome. The aim of the present biomechanical work is to compare different stabilization techniques for the symphysis, of which the two plate/screwless procedures can also be inserted minimally invasively.

**Materials and methods:**
Testing was performed on human pelvis models (Pelvis with Partial Fusion, 4th Gen., Composite, 17 PCF Solid Foam Core, Large; Sawbones Europe AB, Malmoe, Sweden) in which an open-book injury was placed (Burgess/Young APC II).
The following 5 groups (5 pelvises per group) were studied:

*Group A: Supraacetabular external fixator [1].*
*Group B: Symphysis-bridging plate osteosynthesis [2].*
*Group C: internal fixator (INFIX) [3].*
*Group D: plate/screwless stabilization using suture-buttons and tightropes [4].*
*Group E: plate/screwless stabilization using suture anchors and fibretapes [5].*

Using a biomechanical testing machine (Instron 8871, Norwood, MA, USA), stability testing was then performed according to the following protocol: Axial cyclic loading of 440N at 1Hz for 200,000 cycles (corresponding to partial loading of 20kg for 6 weeks, which is standard post-treatment after stabilization of the pelvic ring).
For this purpose, the movement of the symphysis in the horizontal (opening) and vertical (shearing) direction was measured using an optical system (ARAMIS 5 M System, Gom GmbH, Germany).
The stability of each osteosynthesis was evaluated by comparing the different groups based on the cyclic tests.

**Results:**
The 5 groups differed significantly in stability. The most stable restoration in the horizontal direction (opening) was found in groups B and C (0.01mm each), followed by group D (0.04mm), group E (0.07mm) and group A (0.16mm) (Figure 1). The greatest stability in the vertical direction (shearing) was found in group D (0.03mm), followed by groups B (0.04mm), A (0.07mm), E (0.09mm) and C (0.11mm) (Figure 2).

**Conclusion:**

The plate/screwless osteosynthesis with suture buttons and tight-rope is superior to the external fixator, but do not achieve the stability of the plate osteosynthesis. Compared to the INFIX, both plate/screwless osteosyntheses are superior in terms of vertical stability. Thus, the plate/screwless procedures offer the possibility of stable treatment of an unstable symphysis. Next steps should be the clinical application of the minimally invasive procedures in the context of an observational study.

**References:**

1. Tosounidis TH, Mauffrey C, Giannoudis PV. Optimization of technique for insertion of implants at the supra-acetabular corridor in pelvis and acetabular surgery. Eur J Orthop Surg Traumatol. 2018; 28(1):29-35.
2. Grimshaw CS, Bledsoe JG, Moed BR. Locked Versus Standard Unlocked Plating of the Pubic Symphysis: A Cadaver Biomechanical Study. J Orthop Trauma. 2012 Jul;26(7):402-6.
3. Vigdorchik JM, Esquivel AO, Jin X, Yang KH, Onwudiwe NA, Vaidya R. Biomechanical stability of a supra-acetabular pedicle screw Internal Fixation device (INFIX) vs External Fixation and plates for vertically unstable pelvic fractures. J Orthop Surg Res. 2012; 7: 31.
4. Kiskaddon EM, Wright A, Meeks BD, Froehle AW, Gould GC, Lubitz MG, Prayson MJ, Horne BR. A biomechanical cadaver comparison of suture button fixation to plate fixation for pubic symphysis diastasis. Injury. 2018 Nov;49(11):1993-1998.
5. Arner JW, Albers M, Zuckerbraun BS, Mauro CS. Laparoscopic Treatment of Pubic Symphysis Instability With Anchors and Tape Suture. Arthrosc Tech. 2017 Dec 11;7(1):e23-e27.

**Funding:**
Deutsche Forschungsgemeinschaft (DFG), Project-No.: 446641912

**Figure 1:**

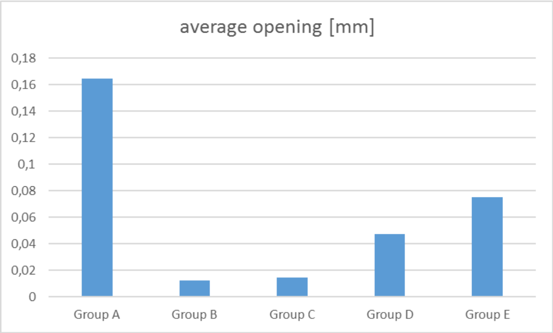

**Figure 2:**

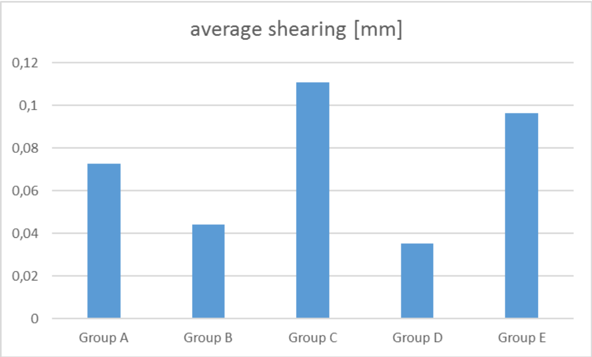

### Anti-inflammatory and analgesic effects of a novel α9α10 nicotinic acetylcholine receptor agonist, pCN-N,N-diethyl-N′-phenyl-piperazine

ID: 33

**SCF - Novel therapeutics, Biomarker, Targeted Therapy**

Katrin Richter[1], Sara M. Herz[2], Clare Stokes[3], Andreas Hecker[1], M. Imad Damaj[2], Veronika Grau[1], Roger L. Papke[3]

[1]*Department of Justus-Liebig University of Giessen, Department of General and Thoracic Surgery, Laboratory of Experimental Surgery, Giessen, Deutschland*, [2]*Virginia Commonwealth University, Medical College of Virginia, Department of Pharmacology and Toxicology, United States*, [3]*University of Florida, Department of Pharmacology and Therapeutics, United States*

**Background:**

Nicotinic acetylcholine receptors (nAChRs) have been increasingly studied for their immune regulatory and analgesic functions. We discovered that activation of nAChRs containing subunits α7, α9 and α10 inhibits the function of the ATP-sensitive P2X7 receptor and down-regulates the maturation and release of inflammasome-dependent cytokines including interleukin (IL)-1β [**1,2**]. IL-1β is a potent pro-inflammatory cytokine that is involved in host defense against infections. This cytokine plays, however a pivotal role in the pathogenesis of numerous inflammatory diseases such as trauma-induced systemic inflammation. Recent evidence further suggests nAChRs containing subunits α9 and α10 as novel targets for the treatment of inflammatory pain. This prompted us to investigate the effects of the novel α9α10 nAChR ligand *p*CN-N,N-diethyl-N′-phenyl-piperazine (*p*CN-diEPP) in an *in vitro* model for inflammation and in an *in vivo* model for inflammatory pain.

**Materials and methods:**

*p*CN-diEPP activity was characterized by electrophysiological two-electrode voltage-clamp measurements on mixed populations of human α7 and α9α10 nAChRs expressed in *Xenopus laevis* oocytes. Human monocytic THP-1 cells and primary peripheral blood mononuclear cells (PBMCs) from healthy volunteers (female and male) were primed with lipopolysaccharide (LPS). Thereafter, the effect of the absence or presence of *p*CN-diEPP on the ATP-induced IL-1β release was measured by an enzyme-linked immunosorbent assay. A murine model for inflammatory pain that is induced by injection of complete Freund's adjuvant (CFA) into the hind paw was used to test for analgesic effects of *p*CN-diEPP (intraperitoneal injection of 0.1, 0.3, and 1 mg/kg body weight). Experiments were performed on wild-type mice and on α7 knockout mice. Three days after intraplantar injection of CFA, the tactile allodynia was as measured by manual plantar stimulation with von Frey filaments. Experimental animals received humane care according to NIH “Guide for the Care and Use of Laboratory Animals.”

**Results:**

*p*CN-diEPP was a full agonist of human α9α10 nAChRs expressed in *X. laevis* oocytes with an EC50 of 2.6 ± 0.6 µM. Moreover, *p*CN-diEPP showed strong anti-inflammatory effects by efficiently inhibiting the ATP-mediated release of IL-1β by THP-1 cells, and primary human PBMCs. In the murine model for inflammatory pain, 0.3 mg/kg *p*CN-diEPP attenuated the CFA-induced mechanical hypersensitivity, while 1 mg/kg of *p*CN-diEPP fully reversed mechanical hypersensitivity. The analgesic effect of *p*CN-diEPP was retained in α7 knockout mice, indicating that its mode of action was independent of α7 nAChRs.

**Conclusion:**

The α9α10 nAChR ligand *p*CN-diEPP efficiently down-modulates the ATP-mediated release of monocytic IL-1β and offsets CFA-induced inflammatory pain in an α7 nAChR-independent manner. Our findings highlight the utility of synthetic α9α10 nAChR agonists for the treatment of inflammatory diseases including trauma-induced sterile inflammation and for the development of non-opiate analgesics.

[1] Richter et al. 2022, Front. Pharmacol. 13:981276.
[2] Richter et al. 2023, Front. Immunol. 14:1140592.

### Exosomal ROR1 in peritoneal fluid identifies peritoneal disseminated PDAC and is associated with poor survival

ID: 112

**SCF - Novel therapeutics, Biomarker, Targeted Therapy**

Anke Mittelstädt[1], Anna Anthuber[1], Paul David[1], Maximilian Brunner[1], Christian Krautz[1], Anne Jacobsen[1], Susanne Merkel[1], Lotta Roßdeutsch[1], Bettina Kloesch[1], Izabela Swierzy[1], Frederik Hansen[1], Deike Strobel[2], Yurdagül Zopf[2], Carol-Immanuel Geppert[3], Andreas Gießl[4], Sebastian Lettmaier[5], Sabine Semrau[5], Robert Gruetzmann[1], Dina Kouhestani[1], Georg Weber[1]

[1]*Chirurgische Klinik, Uniklinikum Erlangen, Erlangen, Deutschland*, [2]*Medizinische Klinik 1, Uniklinikum Erlangen, Erlangen, Deutschland*, [3]*Pathologie, Uniklinikum Erlangen, Erlangen, Deutschland*, [4]*Augenklinik, Uniklinikum Erlangen, Erlangen, Deutschland*, [5]*Strahlenklinik, Uniklinikum Erlangen, Erlangen, Deutschland*

**Background:**
Pancreatic ductal adenocarcinoma (PDAC) is one of the deadliest forms of cancer and peritoneal dissemination is one major cause for this poor prognosis. Exosomes have emerged as promising biomarkers for gastrointestinal cancers and can be found in all kinds of bodily fluids, also in peritoneal lavage fluid and ascites (PF). Both are unique samples due to their closeness to gastrointestinal malignancies. The receptor tyrosine kinase-like orphan receptor 1 (ROR1) has been identified as a potential biomarker in human cancers and represents a promising target for an immunotherapy approach, which could be considered for future treatment strategies. Here we prospectively analyzed the exosomal surface protein ROR1 (exo-ROR1) in PF in localized, locally advanced and oligo-metastasized PDAC patients (PER-) on the one hand and peritoneal disseminated tumor stages (PER+) on the other hand followed by the correlation of exo-ROR1 with clinical-pathological parameters.

**Materials and methods:**
Exosomes were isolated from PF and plasma samples of non-cancerous (NC) (n = 15), chronic pancreatitis (CP) (n = 4), localized, locally advanced or oligo metastasized PDAC (PER-) (n = 18) and peritoneal disseminated PDAC (PER+) (n = 9) patients and the surface protein ROR1 was detected via FACS analysis. Additionally, soluble ROR1 in PF was analyzed. Exosome isolation was proven by Nano Tracking Analysis (NTA), Western Blots, Transmission electron microscopy (TEM), and BCA protein assay. ROR1 expression in tissue was investigated using Western blots and qPCR. The results were correlated with clinical data and survival analysis was performed.

**Results:**
PDAC (PER+) patients have the highest exo-ROR1 concentration in PF and can be discriminated from NC ($p < 0.0001$), PDAC (PER-) ($p < 0.0001$), and CP ($p = 0.0112$). Likewise, PDAC (PER-) can be discriminated from NC ($p = 0.0003$). In plasma Exo-ROR1 is not able to distinguish between the groups. High exo-ROR1 expression is associated with lower survival ($p = 0.0482$).

**Conclusion:**
With exo-ROR1 in PF we found a promising diagnostic and prognostic biomarker possibly discriminating between NC, PDAC (PER-) and PDAC (PER+) and might shed light on future diagnostic and therapeutic concepts in PDAC.

**Targeting the regenerative liver niche in colorectal cancer liver metastasis organoids using AURKA inhibition**
ID: 215

**SCF - Novel therapeutics, Biomarker, Targeted Therapy**

Jean-Paul Bereuter[1], Dylan Liabeuf[1], Tim Schmäche[1 2], Stephan Drukewitz[2], Beatrix Jahnke[1], Sascha Brückmann[3], Vivian Mittne[1], Heike Polster[1], Therese Seidlitz[1], Mark Enrik Geißler[1], Meritxell Huch[4], Jürgen Weitz[1 2], Carina Riediger[1], Thilo Welsch[5], Daniel Stange[1 2], Franziska Baenke[1]

[1]*Department of Visceral, Thoracic and Vascular Surgery, Medical Faculty and University Hospital Carl Gustav Carus, Dresden, Deutschland*, [2]*National Center for Tumour Diseases (NCT), Medical Faculty and University Hospital Carl Gustav Carus, Dresden, Deutschland*, [3]*Institute of Pathology and Tumour- and Normal Tissue Bank of the University Cancer Center (UCC), Medical Faculty and University Hospital Carl Gustav Carus, Dresden, Deutschland*, [4]*Max Planck Institute of Molecular Cell Biology and Genetic, Dresden, Deutschland*, [5]*Department of General, Visceral and Thoracic Surgery, University Medical Center Hamburg-Eppendorf, Hamburg, Deutschland*

**Background:**
Colorectal cancer is one of the most common cancers globally. The appearance of liver metastasis (CRCLM) in colorectal cancer patients is frequently observed. Multimodal treatment of liver metastases encompasses the surgical removal of the metastasis as the only curative treatment option for these patients. However, approximately two-thirds of patients will present with recurrent liver metastasis within two years following hepatic resections. After surgical CRCLM resection, the release of mitogenic cytokines induces a liver regeneration process. The influence of these mitogenic signals on remaining micrometastases is unclear.

**Materials and methods:**
In this study, CRCLM PDOs (n=10) were generated, characterised and exposed to media that mimics either the regenerative liver niche or the intestinal niche of origin. The PDOs' response to these medium conditions was assessed regarding morphology and its proliferative capacity. By using drug screenings, potential drug candidates were identified and validated under liver regeneration conditions in a collateral damage co-culture model of CRCLM PDOs and corresponding normal cholangiocyte PDOs.

**Results:**
CRCLM PDOs retained histopathological and genetic features of the originating tissue. The different media conditions of the PDOs showed divergent responses to the regenerative liver niche depending on their mutational burden. Drug library screenings revealed aurora kinase inhibitors (AKIs) as potential drug candidates for targeting the growth of CRCLM PDOs. Co-cultivation of CRCLM PDOs with normal cholangiocyte PDOs showed that AKIs predominantly affected CRCLM PDOs while not impairing the growth of their normal counterparts *in-vitro*.

**Conclusion:**
In this study, we investigated the impact of the regenerative liver niche on CRCLM PDOs and how to prevent the action of these growth supporting signals on cancer cells. Firstly, our study revealed the usefulness of PDOs to investigate tumour dynamics in response to a changing microenvironment. Considering the observed selective killing of AKIs in the co-culture experiments, AKIs might constitute potential drug candidates to prevent tumour recurrence from remaining micrometastases without impairing liver regeneration after surgery. Further, *in-vivo* studies are now needed to test their potential to prevent intrahepatic tumour recurrence.

**High CD71 expressing Neutrophils Correlate with Reduced Outcome in Pancreatic Ductal Adenocarcinoma Patients**
ID: 333

**SCF - Novel therapeutics, Biomarker, Targeted Therapy**

Paul David[1], Frederik Hansen[1], Anke Mittelstädt[1], Susanne Merkel[1], Bettina Kloesch[1], Anne Jacobsen[1], Maximilian Brunner[1], Christian Krautz[1], Robert Grützmann[1], Georg Weber[1]

[1]*University Hospital Erlangen, Department of Surgery, Erlangen, Deutschland*

**Background:**
Pancreatic ductal adenocarcinoma (PDAC) is one of the most lethal malignancies, presenting a persisting global health burden. PDAC has shown poor response to conventional chemotherapies and immunomodulatory strategies. Neutrophils have a double-edged role in tumor progression exhibiting both pro-tumor and anti-tumor functions. CD71, also known as transferrin receptor 1, performs a critical role in cellular iron uptake and is highly expressed on proliferating cells, and especially on activated immune cells. CD71 is known to be elevated in various types of solid cancers and is associated with poor prognosis, however, the expression of CD71 on neutrophils in PDAC and its potential clinical impact is obscure.

**Materials and methods:**
Peripheral blood was collected from 22 clinical control and 60 PDAC patients who underwent elective surgery at the Department of Surgery of the University Hospital Erlangen between 2020 and 2023 were eligible to be enrolled in the present study. We used Flow cytometry and LEGENDPlex™ bead-based immunoassays in this study.

**Results:**
We analyzed CD71 on circulating neutrophils in PDAC and clinical control patients and found a significant increased expression in PDAC patients. Furthermore, high expression of CD71 on neutrophils in PDAC patients was associated with reduced outcome compared to low expression. Additionally, CD71 on neutrophils correlated positively with the levels of proinflammatory cytokines IL-6, IFN-γ, CD40-L, and BAFF in plasma of PDAC patients. Finally, we have demonstrated that high expression of CD71 on neutrophils was also associated with an increased expression of CD39 and CD25 on circulating T-cells.

**Conclusion:**
We hypothesize that CD71 on neutrophils is associated with tumor progression in PDAC. Further studies are needed to investigate the distinct functionality of CD71 expressing neutrophils and their potential clinical application.

**Local lymphatic T-bet regulates tumor specific CD8+ T cells responses during pancreatic ductal adenocarcinoma**
ID: 336

**SCF - Novel therapeutics, Biomarker, Targeted Therapy**

Paul David[1], Frederik Hansen[1], Anke Mittelstaedt[1], Anne Jacobsen[1], Susanne Merkel[1], Maximilian Brunner[1], Christian Krautz[1], Robert Gruetzmann[1], Georg Weber[1]

[1]*University Hospital Erlangen, Department of Surgery, Erlangen, Deutschland*

**Background:**
Pancreatic ductal adenocarcinoma (PDAC) is a devastating disease with one of the lowest 5-year survival rates of all solid tumors. Although multiple therapeutic approaches have been explored to improve the survival of PDAC patients, the outcome is poor. Henceforth, the exploration of a promising biomarker to assist in a patient’s treatment is of utmost importance. Here, we investigate the role of T-bet, a transcription factor that has a crucial role in the activation and differentiation of several immune cells in the blood and lymph nodes of PDAC patients.

**Materials and methods:**
We collected lymph node from station 8a (LN8a) from PDAC patients who underwent open or laparoscopic surgery and lymph node from station 12b (LN12b) from patients who underwent cholecystectomy at the Department of Surgery, University Hospital Erlangen. LN12b served as clinical controls for this study. We correlated the findings with patient’s clinic pathological parameters.

**Results:**
Increased relative mRNA expression of lymphatic T-bet was associated with reduced overall survival in PDAC patients. In the LN, $CD3^+$ T cells produced the maximum T-bet. Furthermore, patients with tumor infiltrated LN revealed decreased T-bet expressing cytotoxic and helper T cells. T-bet expressing $CD4^+$ and $CD8^+$ T cells positively correlated to PD-1 expressing $CD4^+$ and $CD8^+$ T cells and T-bet expressing $CD8^+$ T cells were negatively correlated to the expression of IL-6 and TNF-α in the LN. Interestingly, patients treated with neoadjuvant chemotherapy showed increased T-bet expressing $CD4^+$ and $CD8^+$ T cells.

**Conclusion:**
Our study unravels T-bet as a prognostic factor during PDAC and underscore the potential significance of the CD8-T-bet-axis as an immunotherapeutic target, especially in patients with high T-bet expression.

**Inhibition of Transglutaminase 2 and Sorafenib: A synergistic approach against hepatocellular carcinoma**
ID: 493

**SCF - Novel therapeutics, Biomarker, Targeted Therapy**

Amalia Katharina Haxhijaj[1], Elsie Oppermann[1], Wolf Otto Bechstein[1], Patrizia Malkomes[1 2 3]

[1]*Goethe University Hospital Frankfurt , Department for General, Visceral, Transplant and Thoracic Surgery, Frankfurt am Main, Deutschland*, [2]*Frankfurt Cancer Institute, Frankfurt am Main, Deutschland*, [3]*German Cancer Consortium (DKTK) and German Cancer Research Center (DKFZ), Heidelberg*

**Background:**
Transglutaminase 2 (TGM2) is a multifunctional enzyme that exerts either transamidase or G protein activity. TGM2 plays crucial roles in cancer-related processes such as cancer cell survival, angiogenesis, invasion and metastasis. TGM2 has also been regarded as a valuable target for cancer treatment and TGM2 inhibitors are currently undergoing clinical investigations.
In hepatocellular carcinoma (HCC), increased TGM2 expression has been observed, mainly in tumors prone to early recurrence. Further, TGM2 has been strongly implicated in drug resistance and HCC metastasis. However, the potential of TGM2 inhibitors in treating HCC remains unexplored.

**Materials and methods:**
We assessed TGM2 expression and enzymatic activity in three HCC cell lines: HepG2, Huh7 and SK-Hep-1. We investigated the impact of TGM2 inhibition, using small molecule inhibitors (ZDON and LDN27219), on cell expansion and apoptosis of these cell lines. Apoptosis related proteins triggered by TGM2 inhibition were identified by proteome profiler apoptosis array and Western Blot analysis was performed to validate these apoptotic proteins. Lastly, we determined the synergistic effects of TGM2 inhibitors in combination with sorafenib on cell expansion and apoptosis.

**Results:**
TGM2 expression and enzymatic activity were detected in all three HCC cell lines, with SK-Hep-1 exhibiting the highest expression and activity. Inhibition of TGM2 using ZDON and LDN27219 significantly reduced cell expansion by apoptosis induction. Proteome profiler apoptosis array revealed an increase in pro-apoptotic proteins, including FAS, FADD and TRAIL/R2 and an inhibition of the anti-apoptotic protein HSP29. These results were confirmed by Western Blot analysis.
A combined treatment of TGM2 inhibitors with sorafenib resulted in a synergistic induction of apoptosis through activation of the FAS/FADD/TRAIL/R2 pathway.

**Conclusion:**
Our results show that TGM2 inhibitors reduce cancer cell expansion in HCC cell lines and synergistically enhance the antiproliferative activity of the chemotherapeutic drug Sorafenib. These data suggest TGM2 as a potential therapeutic target in HCC by inducing apoptosis through an activation of the FAS/FADD/TRAIL/R2 pathway and support the rational development of new combination therapies targeting TGM2.

**Transglutaminase 2: A promising prognostic marker and therapeutic target in pancreatic cancer**
ID: 496

**SCF - Novel therapeutics, Biomarker, Targeted Therapy**

Laura Laußer[1], Jiaoyan Han[1], Elsie Oppermann[1], Johannes Lorenz[1], Sara Fatima Faqar-Uz-Zaman[1], Paul Ziegler[2 3], Peter Wild[2 3], Wolf Bechstein[1], Michael A. Rieger[4 5 6 7], Patrizia Malkomes[1 5 6]

[1]*Department of General, Visceral, Transplant and Thoracic Surgery, Goethe University Hospital, Frankfurt, Deutschland*, [2]*Dr. Senckenberg Institute of Pathology, Goethe University Hospital, Frankfurt, Deutschland*, [3]*University Cancer Center (UCT), Frankfurt, Deutschland*, [4]*Department of Medicine II, Hematology/Oncology, Goethe University Hospital, Frankfurt, Deutschland*, [5]*Frankfurt Cancer Institute, Frankfurt, Deutschland*, [6]*German Cancer Consortium (DKTK), German Cancer Research Center (DKFZ), Partner site Frankfurt/Mainz, Deutschland*, [7]*Cardio-Pulmonary-Institute, Frankfurt, Deutschland*

**Background:**
Pancreatic cancer (PDAC) is one of the most lethal cancers with poorest prognosis. To date, surgery remains the only curative treatment option, but it is accessible to only about 20% of all patients. Despite improvements in chemotherapeutic regimens, the majority of patients will develop tumor recurrence. Therefore, the identification of new prognostic and therapeutic targets are urgently needed and may provide immediate clinical benefits. Transglutaminase 2 (TGM2) is a multifunctional enzyme with transamidase and GTPase activity and has been implicated in the initiation and progression of various cancer types. In the context of PDAC, TGM2 has been associated with the development of drug resistance and metastasis. However, the clinical significance of TGM2 in PDAC patients has remained uncertain. Therefore, we determined the expression of TGM2 in a PDAC patient cohort and investigated the therapeutic potential of TGM2 inhibition in combination with gemcitabine.

**Materials and methods:**
We constructed Tissue microarrays containing samples of 165 clinically characterized PDAC patients and profiled TGM2 protein expression using immunohistochemical staining.
Further we assessed TGM2 expression and enzymatic activity in five PDAC cell lines (AsPC-1, BxPC-3, DAN-G, MIA PaCa-2 and PA-TU-8902). In our *in vitro* studies, we investigated the impact of TGM2 inhibitors (Z-DON, LDN-27219 and GK 921) on cell expansion and apoptosis. To identify apoptosis-related proteins triggered by TGM2 inhibition, we conducted a Proteome profiler apoptosis array. Lastly, we evaluated the synergistic effects of combining TGM2 inhibitors with gemcitabine.

**Results:**
TGM2 expression was upregulated in matched tumor samples in comparison to adjacent normal pancreatic tissue. A strong TGM2 expression predicted worse prognosis regarding overall and recurrence-free survival. Results from the multivariate analysis identified TGM2 as an independent risk factor for poor overall and disease-free survival.
In our functional analysis, pharmacologic inactivation of TGM2 using small-molecule inhibitors reduced cancer cell expansion in PDAC cell lines by inducing apoptosis. The Proteome profiler apoptosis array revealed that TGM2 inhibition resulted in an activation of the tumor suppressor p53 and a downregulation of anti-apoptotic proteins (HSP60, HSP27, HSP70, Survivin). Furthermore, TGM2 inhibitors demonstrated a synergistic enhancement of the anti-proliferative activity of the chemotherapeutic drug gemcitabine through the activation of p53-mediated apoptosis, as confirmed by Western Blot analysis.

**Conclusion:**
These data indicate that TGM2 could serve as an independent prognostic marker, offering improved risk stratification of PDAC patients. Additionally, the pharmacological inhibition of TGM2 triggers apoptosis in cancer cells through p53 activation, suggesting its potential as a promising target for a combination therapy approach in personalized PDAC treatment.

**Prognostic Value of Dipeptidyl Peptidase 3 (DPP3) and Bioactive Adrenomedullin (bio-ADM) in Esophagectomy: A Prospective Observational Study**
ID: 511

**SCF - Novel therapeutics, Biomarker, Targeted Therapy**

Jan Bardenhagen[1], Courtney Metz[1], Christine Torrey[1], Jakob Izbicki[1], Asmus Heumann[1], Valerie Isabel Nottberg[1]

[1]*Universitätsklinikum Hamburg Eppendorf, Klinik für Allgemein-, Viszeral- und Thoraxchirurgie, Hamburg, Deutschland*

**Background:**
Dipeptidyl Peptidase 3 (DPP3), a recently discovered biomarker known to be responsive to oxidative stress, has garnered significant attention due to its potential implications in cardiovascular and renal functions. Elevated DPP3 levels have been consistently linked to adverse clinical outcomes, particularly in cases involving cardiogenic shock. Bioactive adrenomedullin (bio-ADM) is a peptide with vasodilatory and diuretic properties, and its release is triggered by endothelial dysfunction, notably in conditions such as septic shock. Associations between bio-ADM and postoperative complications have been identified in the context of vascular surgery. Esophageal surgeries are often complicated by various postoperative issues, including anastomotic insufficiencies, pneumonias, and subsequent septic presentations. Notably, there is a dearth of comprehensive perioperative measurement studies within the domain of visceral surgical procedures, limiting our ability to definitively assess the prognostic value of these biomarkers in predicting patient outcomes.

**Materials and methods:**
This single-center, prospective, observational study was conducted at the University Medical Center Hamburg-Eppendorf and involved 20 high-risk patients undergoing esophagectomy. Pertinent perioperative data, clinical particulars, vital signs, and routine blood assessments were systematically gathered. DPP3 and Bio-Adrenomedullin (Bio-ADM) levels were quantified both before and after surgery on postoperative day 1, 3, 5, and 7 using the Nexus IB10 Analyzer.

**Results:**
20 patients (18 male and 2 female) underwent esophagectomy. Mortality rate within 30 days of surgery was 5%, morbidity rate was 75%. Median levels of DPP3 in patients undergoing esophagectomy exceeded reference ranges established for healthy individuals. Postoperative DPP3 levels were notably higher in patients with complications (21.89 ng/mL vs. 14.0 ng/mL) and exhibited an incremental trend with the severity of complications. There is a strong correlation between elevations in DPP3, CRP, and IL-6. Admission levels of DPP3 and rapid increases therein were predictive of sepsis outcomes. Elevated DPP3 levels exhibited a significant correlation with SOFA scores ($p=0.002$), suggesting a potential link to adverse outcomes. Additionally, a strong correlation between DPP3 elevations and anastomotic insufficiencies was identified ($p=0.2866$). However, due to the limited sample size ($n=5$ insufficiencies), statistical significance was not achieved. Additionally, elevated levels of bio-ADM postoperatively were observed to be associated with an unfavorable patient outcome subsequent to esophageal surgery. These elevated bio-ADM levels exhibited correlations with postoperative morbidity indicators such as sepsis and pneumonia, as well as an increased mortality rate, although the statistical significance was not reached ($p=0.129$).

**Conclusion:**
Both DPP3 and bio-ADM hold promise as biomarkers for the prediction of postoperative complications. Due to sample size correlations did not reach level of statistical significance. These findings suggest the potential utility of DPP3 and bio-ADM as biomarkers for risk assessment and outcome prediction in esophagectomy patients, warranting further investigation with larger cohorts.

Abstracts – DCK 2024 – 141st Congress of the German Society of Surgery – Leipzig, April 24–26 • DOI 10.1515/iss-2024-2008
Innov Surg Sci 2024; 9 (Special Suppl 1): s303–s385

**HBD1 is upregulated in biliary atresia, associates with disease stage and predicts native liver survival**
ID: 514

**SCF - Novel therapeutics, Biomarker, Targeted Therapy**

Christoph Slavetinsky[1], Jule Basenach[2], Pascal Damm[1], Claus Petersen[3], Ekkehard Sturm[2], Jörg Fuchs[1]

[1]*University Children's Hospital Tübingen , Pediatric Surgery and Urology, Tübingen, Deutschland*, [2]*University Children's Hospital Tübingen, Pediatric Gastroenterology and Hepatology*, [3]*Hannover Medical School, Pediatric Surgery*

**Background:**
Biliary atresia (BA) is characterized by progressive inflammatory, fibro-obliterative obstruction of bile ducts and subsequent liver fibrosis/cirrhosis. Despite an unclear pathogenesis, recent studies favor an etiology through microbe-triggered immune dysregulation in liver and bile ducts. Antimicrobial peptides (AMP) belong to the first line of innate immunity against invading microbes and possess regulatory functions to control inflammation. The AMP human beta-defensin 1 (hBD1) was found the be upregulated in adult liver disease and to be a highly predictive serum marker of mortality in patients with acute-on-chronic liver failure. The role of AMPs in hepatic physiology and pathophysiology in children is unknown.
We aimed to characterize expression of hBD1 in BA compared to normal and disease control liver tissue and to associate expression patterns with clinical course and outcome after Kasai portoenterostomy (KPE).

**Materials and methods:**
Gene expression of hBD1, its activator thioredoxin (TXN) and the profibrotic activity marker TGFß were analyzed by qPCR in liver tissue from patients with BA at early (eBA, at KPE; n = 33) and late (lBA, at LTX; n = 37) liver disease stages, both compared to age-matched normal liver tissue (n = 18), and familial cholestatic disorders (eFCD = 14; lFCD = 8) as disease controls. Results were assessed for native liver survival by Kaplan-Meier estimator and for clearance of jaundice after KPE, matched with age at biopsy/disease state, observed during course of disease, and correlated with liver fibrosis (TGF-ß and histologic Ishak fibrosis score) and cholestasis markers (serum bilirubin) by regression analysis. Serum levels of hBD1 in patients with BA compared to age-matched healthy children were analyzed by ELISA. The expression of the murine hBD1 homolog mouse beta-defensin 1 (mBD1) was analyzed in liver tissue from a rhesus rotavirus (RRV) mouse model of BA. MBD1 liver expression was analyzed in mice w/o virus, mice receiving virus but w/o development of BA, and mice receiving virus and developing BA. Group comparisons were made with One-way ANOVA using GraphPad Prism®.

**Results:**
Expression of hBD1 is differentially regulated in BA of both men and mice (mBD1). Liver expression of mBD1 is upregulated in an RRV mouse model of BA compared to non-infected mice and mice receiving RRV but w/o developing BA (P = 0.01). hBD1 is upregulated in patients of late BA (and late FCD) compared to early BA (P = <0.0001) and normal age-matched liver tissue (P = <0.0001). HBD1 is upregulated during course of disease and increases with age at biopsy. HBD1 expression in BA does correlate with TGFß expression ($R^2 = 0.17$, P = 0.01) and Ishak fibrosis score ($R^2 = 0.15$, P = 0.009) as markers for liver fibrosis but not with bilirubin serum levels (as cholestasis marker) ($R^2 = 0.002$, P = 0.86). Serum levels of hBD1 are upregulated in patients with BA compared to age-matched healthy children (P = <0.0001). Increased expression of hBD1 in early BA is associated with a failed clearance of jaundice after KPE (P = 0.03) and with shorter native liver survival ($X^2 = 9.8$, P = 0.002).

**Conclusion:**

HBD1 is differentially regulated in both human and experimental BA characterized by an exclusive upregulation in advanced stages of all pediatric cholestatic diseases. Determining the stage of the disease when diagnosing BA is difficult which often complicates therapy decisions and increases mortality in such patients. Increased expression of hBD1 at time of diagnosis can detect advanced liver fibrosis and progressed disease associating with failure of KPE and shorter native liver survival. Thus, hBD1 is a highly predictive biomarker early in pediatric cholestatic disease (and likely beyond) assessing the risk of liver failure and need for transplantation.

**RUNX1 as a potential biomarker for BET inhibitor resistant pancreatic cancer**
ID: 686

**SCF - Novel therapeutics, Biomarker, Targeted Therapy**

Leonie Witte[1], Ilaria Deidda[1], Christian Schneeweis[2], Judith Achler-Galitzki[1], Michael Ghadimi[1], Günter Schneider[1], Matthias Wirth[1 3 4]

[1]*University Medical Center Göttingen, Department of General, Visceral and Pediatric Surgery, Göttingen, Germany*, [2]*Technical University Munich, Institute for Translational Cancer Research and Experimental Cancer Therapy, Munich, Germany*, [3]*Charité Universitätsmedizin Berlin, Department of Hematology, Oncology and Cancer Immunology, Campus Benjamin Franklin, Charité - Universitätsmedizin Berlin, Corporate Member of Freie Universität Berlin and Humboldt-Universität zu Berlin, Berlin, Germany*, [4]*Max-Delbrück-Center for Molecular Medicine, Berlin, Germany*

**Background:**
Pancreatic ductal adenocarcinoma (PDAC) is estimated to be the second most common fatal cancer in humans by 2030 and is showing resistance to various therapeutic strategies. Epigenetic readers, such as the bromodomain and extra terminal domain (BET) protein family facilitate various cellular processes, such as transcription and chromatin remodeling. BET inhibitors are a promising class of drugs that specifically inhibit the BET protein family, including BRD4. Such BET inhibitors can disrupt the interaction between BRD4 and chromatin and are currently being tested in clinical trials, with variable responses.

**Materials and methods:**
We performed an unbiased compound screen with 1842 pharmacological compounds that included experimental preclinical anti-cancer drugs as well as FDA-approved drugs. We performed this screen in human and murine PDAC cell lines categorized as the aggressive squamous/basal-like subtype. Using CRISPR technology, we performed both genetic knockouts and endogenous activation (CRISPR activation) models in isogenic cells. To investigate our hypotheses on a global and unbiased level, we performed multiple *omics* experiments, such as ATACseq, ChIPseq and RNAseq, in addition to comprehensive database analyses. Single and combination treatments were analyzed using live cell imaging and quantitative endpoint measurements to determine viability (ATP quantification, metabolic activity), cell cycle (flow cytometry) and cell death (Annexin V/PI). Furthermore, immunoblots and qPCR assays were performed to validate individual signaling pathways and targets. The pharmacological interventions were additionally tested in different murine and human cell line models, predictive patient derived organoid (PDO) models and in cell line derived xenograft (CDX) models.

**Results:**
Starting from an unbiased screening approach, we identified RUNX1 as a potential therapeutic target in PDAC. We show that small molecule inhibition of RUNX1 is associated with enrichment of H3K27ac, an active chromatin mark, and the chromatin reader protein BRD4. The combined inhibition of RUNX1 and BET proteins (such as BRD4) synergistically suppresses tumor cell growth in PDAC cell lines, patient derived organoids, and xenograft models. Both genetic and pharmacological inhibition of RUNX1 and BRD4/BET enhance cell death and more potently suppressed advanced PDAC. In aggressive PDAC mesenchymal cells, we also show that de-repression of cell death-associated genes upon combined BET and RUNX1 inhibition is required for the induction of combination therapy-induced cell death in PDAC.

**Conclusion:**
Our data indicate that RUNX1 is a potential biomarker for effective BET inhibitor treatment and identifies a potential epigenetic-based therapeutic option for PDAC patients.

Abstracts – DCK 2024 – 141st Congress of the German Society of Surgery – Leipzig, April 24–26 • DOI 10.1515/iss-2024-2008
Innov Surg Sci 2024; 9 (Special Suppl 1): s303–s385

**Performance of a Novel Somatostatin Receptor Type 2 (SSTR2)-Targeted Near Infrared (NIR)-Fluorescent Probe in Meningioma and Neuroendocrine Tumor (NET) Mouse Models**
ID: 719

**SCF - Novel therapeutics, Biomarker, Targeted Therapy**

Gina Fürtjes[1], Merle Weitzenberg[2], Martin Ullrich[3], Jens Pietzsch[3], Christian Mawrin[4], Kristoffer Riecken[5], Roland Goldbrunner[1], Oliver Plettenburg[2], Oliver Bruns[6], Volker Neuschmelting[1]

[1]*Klinik für Allgemeine Neurochirurgie, Uniklinik Köln, Köln, Deutschland*, [2]*Institut für Medizinalchemie, Helmholtz Zentrum München, München*, [3]*Department of Radiopharmaceutical and Chemical Biology, , Institute of Radiopharmaceutical Cancer Research, Helmholtz-Zentrum Dresden-Rossendorf, Dresden*, [4]*Institut für Neuropathologie, Universitätsklinikum Magdeburg, Magdeburg*, [5]*Abteilung für Stammzelltransplantation mit Forschungsabteilung Zell- und Gentherapie , Universitätsklinikum Hamburg-Eppendorf, Hamburg*, [6]*Nationales Centrum für Tumorerkrankungen (NCT), Universitäts Krebscentrum UCC Dresden, Dresden*

**Background:**
We aim to develop a highly specific and sensitive optical method for intraoperative meningioma and neuroendocrine tumor (NET) imaging to enable tumor visualization and improve the resection completeness by targeting the reliably overexpressed somatostatin receptor type 2 (SSTR2). We have previously shown the proof-of-principle in physiologically SSTR2-positive organs. To determine the full translational potential of the probe, we aimed to test its performance in an SSTR2-expressing meningioma and in a NET (pheochromocytoma) mouse model in vivo.

**Materials and methods:**
Our probe consists of a somatostatin analogue (TATE) linked to a new developed, near-infrared fluorescent dye (sNIR) targeting the SSTR2 receptor. Human anaplastic meningioma IOMM-Lee cells were genetically modified using lentiviral *SSTR2* gene transfer (SSTR2+). Wildtype and SSTR2+ meningioma cell lines were ectopically and intracranially implanted in mice to mimic human meningioma. In the NET model, mouse pheochromocytoma (MPC) cells were implanted in the right shoulder of mice. Tumor-bearing mice were i.v. injected with TATE-sNIR and fluorescence imaged thereafter using identical protocols. A scrambled probe analogue and octreotide for blocking the SSTR2 binding site served as negative controls. The SSTR2 protein status of cells was evaluated using immunohistochemistry (IHC) ex vivo.

**Results:**
The SSTR2+ IOMM-Lee cells showed a significantly higher uptake of TATE-sNIR compared to wildtype cells and controls. In the ectopic meningioma model, a significantly increased uptake of the probe in the SSTR2+ tumors compared to low expressing wildtype tumors was observed. In the orthotopic meningioma model, the signal tumor-to-brain-ratio was extraordinarily high after TATE-sNIR injection (28.7; SD 13.7; n = 5) and significantly decreased after injection of scrambled TATE-sNIR (4.8, SD 2.0; n = 3) and the Octreotide blocking (11.1, SD 2.4; n = 3). The IHC proofs the overexpression of SSTR2. We were able to achieve comparable results in the pheochromocytoma model and observed the best tumor-to-background ratio after 3h post injection of TATE-sNIR.

**Conclusion:**
Our results demonstrate that our new developed targeted fluorescent probe TATE-sNIR is feasible to enable highly specific fluorescent imaging of SSTR2-overexpressing tumor xenograft and allograft models that reliably reflect human meningioma and NET. The extraordinary tumor-to-background ratio potentially enables fluorescent guided surgery in the future and demands to pursue further clinical translation of the probe.

Abstracts – DCK 2024 – 141st Congress of the German Society of Surgery – Leipzig, April 24–26 • DOI 10.1515/iss-2024-2008
Innov Surg Sci 2024; 9 (Special Suppl 1): s303–s385

**Patient-derived organoid cultures as a model system for targeted drug screening assays**
ID: 724

**SCF - Novel therapeutics, Biomarker, Targeted Therapy**

Melanie Spitzner[1], Constanza Tapia[2], Maximilian Hellkamp[2], Gizem Chelik[3], Denise Müller[4], Sushmita Nair[2 5], Constantin Pape[5], Philipp Ströbel[4], Volker Ellenrieder[6], Elisabeth Hessmann[7], Matthias Wirth[2], Michael Ghadimi[2], Günter Schneider[2], Marian Grade[8]

[1]*Department of General, Visceral and Pediatric Surgery, University Medical Center Göttingen, Göttingen, Deutschland*, [2]*Department of General, Visceral and Pediatric Surgery, University Medical Center Göttingen, Göttingen*, [3]*Institute of Cellular and Molecular Immunology, University Medical Center Göttingen, Göttingen*, [4]*Institute of Pathology, University Medical Center Göttingen, Göttingen*, [5]*Institute of Computer Science, Georg-August University Göttingen, Göttingen*, [6]*Department of Gastroenterology, Gastrointestinal Oncology and Endocrinology, University Medical Center Göttingen, Göttingen, Deutschland*, [6]*Department of Gastroenterology, Gastrointestinal Oncology and Endocrinology, University Medical Center Göttingen, Göttingen, Deutschland*, [1]*Department of General, Visceral and Pediatric Surgery, University Medical Center Göttingen, Göttingen, Deutschland*

**Background:**
Patient-derived organoids (PDO) are increasingly being used as model systems because they more closely mimic the in vivo architecture and preserve the genetic landscape of human cancers. Our aim was to genetically and functionally characterize a set of PDO cultures established from rectal and pancreatic cancer.

**Materials and methods:**
A panel of PDOs was established from 20 patients with rectal adenocarcinoma or pancreatic ductal adenocarcinoma (PDAC). Morphological characteristics of these organoid cultures were investigated by microscopy, and the mutational status was acquired by panel sequencing. PDOs were treated with a library of 17 drugs. As readout organoid morphology was captured and quantified using live-cell imaging (Incucyte®), and drug effects on cellular viability was assessed as endpoint measurements using a CellTiter-Glo® assay.

**Results:**
In general, PDOs carried cancer-type specific mutations such as TP53, APC, KRAS, CDKN2, and SMAD4. We observed heterogeneous grow patterns, which included different morphological PDO-types like cystic, regular, filled, unfilled, granular, dark membrane, or light membrane organoids. The drug screens revealed PDO-specific drug-response profiles, ranging from organoids with good response rates to multiple drugs to PDOs with resistant phenotypes to the vast majority of drugs, indicating a remarkable diversity of the drug response of the PDOs.

**Conclusion:**
The PDO cultures of rectal cancer and PDAC remarkably recapitulate the heterogeneity seen in patients. The diversity of the drug treatment responses might be exploitable for functional precision oncology approaches.

**Revolutionizing systemic treatment evaluation in GI cancer care:ctDNA as a unifiable biomarker to predict response to treatment after 2 weeks of chemotherapy at the same cut-off for GEC, PC and CRC**
ID: 812

**SCF - Novel therapeutics, Biomarker, Targeted Therapy**

Patrick Kirchweger[1 2 3 4], Bernhard Doleschal[5], Jonathan Burghofer[6], Simon Schwendinger[7], Emina Jukic[7], Matthias Biebl[1 2], Holger Rumpold[2 3]

[1]*Ordensklinikum Linz, Department of Surgery, Linz, Österreich*, [2]*Johannes Kepler University, Medical Faculty, Linz, Österreich*, [3]*Ordensklinikum Linz, Gastrointestinal Cancer Center, Linz, Österreich*, [4]*VYRAL, Visionary Young Researcher Association Linz, Lichtenberg, Österreich*, [5]*Ordensklinikum Linz, Department of Oncology, Linz, Österreich*, [6]*Ordensklinikum Linz, Laboratory for Molecular Genetic Diagnostics, Linz, Österreich*, [7]*Medical University Innsbruck, Institute of Human Genetics, Innsbruck, Österreich*

**Background:**
ctDNA has emerged as promising biomarker in gastrointestinal cancer. However, definition for detectability and cut-offs are reported very heterogeneously.
We first in man evaluated response to treatment and correlation to actual volumetric tumor burden at a unifiable cut-off for dynamic changes during chemotherapy in 3 different major GI-cancer types (n=924 samples).

**Materials and methods:**
Liquid biopsy samples for n=185 stage IV patients with gastroesophageal (GEC, n=37), pancreatic (PC, n=70) and colorectal cancer (CRC, n=78) have been prospectively acquired pretherapeutically and every 2 weeks during chemotherapy until restaging, analyzed using ddPCR and correlated with response to treatment and actual volumetric tumor burden.

**Results:**
Detection rates were 88.5% (CRC), 77.8% (GEC) and 64.3% (PC). Median pretherapeutic ctDNA MAF was 11.9% (CRC), 1.5% (GEC) and 1.6% (PC).
ctDNA was significantly correlated with total tumor volume, especially with hepatic and lung lesion volume, but not with primary tumor volume in all tumor entities.
Logistic regression revealed a decline of under 58% of the pretherapeutic MAF to predict response to treatment already after 2 weeks (CRC spec. 97.8%, sens. 92.3%, GEC spec. 100%, sens. 66.7%, PC spec. 100%, sens. 91.7%). AUC > 0.9.
This was accompanied with a significant impact on OS and PFS for all tumor entities:
GEC: 3.2 (95%CI 1.9-4.5) vs. 9.5 (95%CI 5.5-13.5) months, $p = 0.001$
PC: 2.5 (95%CI 2.2-2.9) vs. 7.7 (95%CI 4.0-11.3) months, $p < 0.001$
CRC: 2 (95%CI 1.3-2.7) vs. 11 (95%CI 9.0-13.0) months, $p < 0.001$

**Conclusion:**
ctDNA represents an already clinical applicable biomarker with remarkable sensitivity and specificity in displaying actual tumor burden, prediction of prognosis and response to treatment. This biomarker is superior to current gold standard markers CEA, CA19-9 and CA72-4 and predicts response to systemic treatment >80% faster than computed tomography.
ctDNA is able to predict response to treatment of systemic chemotherapy at a unifiable cut-off for metastasized GEC, PC and CRC after 2 weeks of therapy, which may revolutionize palliative treatment of gastrointestinal cancer. These findings indicate also promising applicability in localized stadiums. However, Its value in the neoadjuvant setting still needs to be explored.

**The impact of SATB1 in cell growth regulation in esophageal adenocarcinoma**
ID: 180

**SCF - Oncology**

Iris Zeller[1], Chuang Yang[1], Patrick Sven Plum[1], Thomas Büch[2], Achim Aigner[2], Ines Gockel[1], René Thieme[1]

[1]*Universitätsklinikum Leipzig, Klinik und Poliklinik für Viszeral-, Transplantations-, Thorax- und Gefäßchirurgie, Comprehensive Cancer Center Central Germany*, [2]*Universität Leipzig, Rudolf-Boehm-Institut für Pharmakologie und Toxikologie, Klinische Pharmakologie, Comprehensive Cancer Center Central Germany*

**Background:**
While the incidence of esophageal adenocarcinoma (EAC) is rapidly increasing, its response on cancer treatment remains poor, with a five-year survival rate of 20-40%. Consequently, the discovery of new molecular targets for an efficient therapy of EAC patients is necessary. SATB1 (Special AT-rich sequence-binding protein-1) is a nuclear matrix binding protein, which has been found to play a key role in epigenetic regulation and expression of oncogenes in several solid tumors. Through this regulation, it has a major impact in tumor invasion, proliferation, metastasis, angiogenesis and multidrug resistance. However, its role in esophageal adenocarcinoma is still unknown.

**Materials and methods:**
An in vitro Barrett's esophagus cell culture model was used to determine the SATB1 expression levels by qRT-PCR. The EAC cell lines OE33 and OE19 cells were used to conduct a siRNA-mediated knockdown of SATB1. In siSATB1 transfected cells, proliferation and dead cells were monitored by live cell imaging. Apoptosis and cell cycle were investigated by FACS analysis. Using western blot, the impact of a SATB1-knockdown on autophagy was investigated. Furthermore, senescence was analyzed by p21-mRNA-expression using qRT-PCR. Migration was accessed by transwell assays.

**Results:**
SATB1 was present in all cell lines of the Barrett's sequence (squamous epithelium, metaplasia, high-grade intraepithelial neoplasia and EAC) and had a significantly increased expression in OE33. Through live cell imaging, we discovered that a siRNA-mediated knockdown in tumor cell lines OE33 and OE19 led to a significant stagnation in tumor growth and an increase in dead cells. The mRNA expression of p21, a marker for senescence, was significantly upregulated in siSATB1-transfected OE33 cells. Moreover, an increase in autophagy-specific LC3-II was observed upon siSATB1 treatment in OE33 cells. Cell cycle analysis in siSATB1-transfected cells revealed a significant decrease in G2- and an increase in G1-phase, indicating a decelerated cell cycle in siSATB1 cells. Furthermore, siSATB1 cells had a lower capacity to migrate, as shown in transwell assays.

**Conclusion:**
The role of SATB1 in EAC is barely investigated. Of note, our experiments revealed that SATB1 is overexpressed in the EAC cell line OE33 and plays an important role in cell growth and migration. Based on our current results, it could be shown that the blocking of SATB1 leads to senescence, autophagy and stagnation in the cell cycle. Further investigation is necessary to elucidate the exact molecular pattern of SATB1 to fully understand its role in EAC development and progression.

### ADRB2 receptor in colorectal cancer progression

ID: 183

**SCF - Oncology**

Mathilda Knoblauch[1], Laura Geyer[2], Jens Werner[1], Klaus-Peter Janssen[2], Bernhard W. Renz[1]

[1]*Department of General, Visceral, and Transplantation Surgery, LMU Klinikum, Munich, Germany, München, Deutschland*, [2]*Department of Surgery, Technical University Munich, Munich, Germany, München, Deutschland*

**Background:**
Circulated catecholamines associated with stress have an influence on the tumor microenvironment and are critically involved in cancer progression. Agonists and antagonists of the sympathetic nervous system appear to influence solid tumor metastasis by modulating pathophysiological mechanisms of inflammation, angioneogenesis and epithelial-mesenchymal transition (EMT). We aim to clarify the influence of the sympathetic nervous system on colorectal cancer (CRC) metastasis. The goal of our project is to deepen our understanding of the neuro-cancer crosstalk in CRC metastasis.

**Materials and methods:**
CRC cells and organoids were examined in different treatment options regarding proliferation and migration. Organoids were treated for nine days with the following compounds: Isoproterenol (20µM), Atenolol (ß1 selective blocker, 50µM), ICI 118,551 (ß2 selective blocker, 20µM), or Propranolol (unselective beta blocker, 40µM), as well as by combined administration of 50 µM Atenolol and 20 µM ICI 118,551. Analysis of the proliferative potential of patient-derived colon cancer organoids with respect to treatment with pharmacological inhibitors (beta blockers). Live/dead staining of patient-derived organoids with the dyes Hoechst and SYTOX green, after treatment with the respective compounds.

**Results:**
Isoproterenol and Norepinephrine (NE) promote metastasis in CRC cells by driving EMT. ICI 118,551 (ß2 selective blocker) can inhibit cell and organoid proliferation and migration. ADRB2 mediates the crosstalk between adrenergic signaling and catecholamine derived cancer cell and organoid proliferation and migration. The sympathetic nervous system plays an important role in CRC metastasis.

**Conclusion:**
In colorectal cancer, we suggest that blocking ADRB2 receptor may be clinically useful and provides a preventive strategy. Further detailed studies on the underlying mechanisms are necessary to identify patients with a potential benefit for specific receptor blockade with established drugs for other indications (drug repurposing).

**Machine learning-based semi-automated digital image analysis to assess heterogeneity of personalized drug response ex vivo in pancreatic cancer slice cultures**
ID: 192

**SCF - Oncology**

Benjamin Heckelmann[1], Olha Lapshyna[1], Daniel Fetzner[1], Jannis Duhn[1], Anna Wandmacher[2], Louisa Bolm[1], Kim Honselmann[1], Oliver Schilling[3], Maren Stillger[3], Peter Bronsert[3], Christoph Röcken[4], Susanne Sebens[2], Tobias Keck[1], Ulrich Wellner[1], Rüdiger Braun[1]

[1]*Department of Surgery , University Clinic Schleswig-Holstein, Campus Lübeck, Lübeck, Deutschland*, [2]*Institute for Experimental Tumor Research, Christian-Albrechts-Universität zu Kiel, Kiel, Deutschland*, [3]*Institute of Clinical Pathology, University Clinic Freiburg, Freiburg, Deutschland*, [4]*Institute of Pathology, Christian-Albrechts-Universität zu Kiel, Kiel, Deutschland*

**Background:**
The lack of sufficient treatment options is one of the reasons why the prognosis of pancreatic cancer (PDAC) is limited. For the implementation of personalized therapeutic strategies, patient-derived ex vivo models are of great interest. To address the limitations of the more commonly used ex vivo models, we previously established organotypic slice cultures (OTSC) as an ex vivo model characterized by rapid establishment times and preservation of the multicellular tissue context. To accelerate the use of the ex vivo model, we developed workflows for the classification and recognition of tissue components in an open-access digital pathology program. Using built-in machine learning features, we were able to perform rapid drug response assessment of histopathologic staining in a semi-automated and rapid manner.

**Materials and methods:**
After surgical resection and pathological examination, 20 treatment-naïve, fresh PDAC tissue samples were cut into 300 µm slices using a vibratome and cultured for 3 to 5 days. The slices were subsequently cultured at a liquid-air interface on semi-porous, hydrophilic PTFE tissue culture inserts. After 24 hours of incubation, the standard regimens of gemcitabine, gemcitabine + paclitaxel, and FOLFIRINOX were added to the culture medium. During the cultivation period, slice viability was assessed daily via a resazurin reduction assay. Treatment response was measured by H&E and immunohistochemical (IHC) staining. IHC staining for tissue component differentiation was visualized by CK7 and PanCK for tumor cells and alpha-SMA, vimentin and Sirius red for the stromal compartment. CD3, CD4, CD8, CD68, CD163 were added to assess the immune cell compartment. Ki-67 and cleaved caspase 3 were stained to evaluate proliferation and apoptosis, respectively.
Slides were scanned as virtual whole-slide images (WSI) and analyzed using QuPath digital image analysis software. After setting a threshold for tissue detection and segmenting the tissue areas into tiles for tissue area standardization, we ran the built-in cell detection command based on the optical density of hematoxylin staining. Using the obtained morphological information, such as distinct staining intensity, optical density, and nuclear/cell area ratio between cells, we evaluated heat maps of the features suitable for distinguishing the different cell types. By implementing Haralick features, the digital image analysis tool also allowed us to classify based on the tissue texture surrounding the respective cells. We annotated tumor, stroma, and immune cell populations in 48 WSIs to build cell classifiers for the different histological stains. We then thresholded the staining intensity of the DAB staining to measure positive-stained cells classified as tumor, stroma, or immune. By establishing different scripts for the distinct H&E and IHC staining, we were able to measure the tissue response in a high-throughput-like and reproducible manner.

**Results:**
In untreated OTSCs, the resazurin reduction assay showed a -10% decrease in viability from day 1 to day 3 and day 5. From day 1 to day 5, microscopic histopathologic evaluation of H&E sections did not reveal any significant changes in tumor-to-stroma ratio or significant increase in necrosis during cultivation. Cancerous outgrowth was observed on the surface of OTSCs on several occasions. Immune cell populations remained stable until day 3 and decreased slightly on day 5. No gross changes in proliferation and apoptosis were observed during the cultivation period. However, in a proof-of-principle experiment, the proportion of apoptotic cells was increased after culturing primary PDAC-OTSCs for 15 days. Treatments induced heterogeneous tissue responses in individual patients. Although the overall response of OTSCs after treatment with gemcitabine or gemcitabine-paclitaxel was relatively poor, we identified a subgroup of OTSCs with significantly improved response, as shown by IHC staining, indicating a large response heterogeneity. The response to FOLFIRINOX was more homogeneous, as most FOLFIRINOX-treated OTSCs had significantly fewer proliferating tumor cells and higher rates of necrotic area than gemcitabine-paclitaxel-treated OTSCs. The proportion of apoptotic cells in FOLFIRINOX-treated OTSCs showed a heterogeneous response among patients. A correlation between ex vivo treatment response and survival will be evaluated by the ongoing patient follow-up via the StuDoQ registry of the German Society for General and Visceral Surgery.

**Conclusion:**

The variable response of different patients to the same treatment regimen suggests individual treatment sensitivity and resistance between patients due to intertumoral structural differences in tissue architecture. The combination of tissue and cell morphology-based readout algorithms and the preclinical ex vivo model of OTSCs allows the assessment of the tumor microenvironment in a quantitative manner. The automated workflow leads to a more accurate evaluation of drug response. In addition, the Haralick feature-based assessment of tissue texture could potentially be used to quantify phenotypic intratumor heterogeneity in both treated and untreated conditions. Initial proof-of-principle experiments using spatial transcriptomics and proteomics showed results consistent with immunohistochemical staining and will be expanded to investigate links between phenotypic and molecular response patterns.

**Adrenergic signaling in pancreatic cancer metastasis – Cancer Neuroscience**
ID: 244

**SCF - Oncology**

Mathilda Knoblauch[1], Lisa Fahr[2], Jens Werner[1], Ivonne Regel[2], Bernhard W. Renz[1]

[1]*Department of General, Visceral, and Transplantation Surgery, LMU University Hospital, LMU Munich, Munich, Deutschland*, [2]*Department of Medicine II, LMU University Hospital, LMU Munich, Munich, Deutschland*

**Background:**
Our investigations focus on the ß2-adrenergic receptor (ADRB2) which is reported to be the major mediator for chronic stress induced cancer. The goal of our project is to gain a better understanding of the complex cross-linked nerve and cancer cell mechanisms that can result in pancreatic cancer (PDAC) metastasis.

**Materials and methods:**
By using in vitro models (sphere formation, migration, rt-PCR) we investigate which pathways are involved in PDAC metastasis, and how adrenergic signaling may influence the course of the therapy. We performed RNA sequencing of catecholamine treated PDAC cells and non-treated controls. Immunohistochemistry staining of neuropathological markers was assessed of PDAC primary specimen and matched metastasis. A knockout of *Adrb2* (Adrb2-KO) was generated in murine pancreatic cancer cells using the CRISPR/Cas9 system. The Adrb2-KO cells were were characterized by proliferative and migrative potential. Transcriptional changes were analyzed by RNA sequenzing. We also evaluated patients who continuously use ß-blockers before, during and after PDAC surgery.

**Results:**
Norepinephrine (NE) increased the level of phosphorylated Akt in contrast to phoyphorylated ERK, suggesting that NE could possibly increase PDAC metastasis via Akt signaling pathway. Norepinephrine increases 24 hours growth rate of PDAC cells. We observed a significant reduction in migration after treatment with the specific ADRB2 antagonist ICI 118,551 (ICI). ICI reduced sphere formation, whereas NE enhanced sphere formation capacity. Matched-pair analysis of the median survival of patients with unselective beta-blocker (40 months) compared to the median survival of patients without beta-blockers (21 months) showed significantly longer survival. Immunohistochemistry staining and RNA sequencing are pending.

**Conclusion:**
Adrenergic receptor antagonists could be usefully employed as additive therapy. The clinical availability of adrenergic receptor antagonists would provide highly relevant therapeutic treatment opportunities in the future. We expect that the implementation of this project may contribute to a better understanding of adrenergic signaling pathways in pancreatic cancer.

**Nucleolin mediates the transfer of endocytosed gut bacteria towards the nuclei of circulating tumor cells which consequently promotes colorectal cancer metastasis**
ID: 284

**SCF - Oncology**

Reinhild Feuerstein[1], Christopher Berlin[1], Jost Lünstedt[1], Stefan Fichtner-Feigl[1], Rebecca Kesselring[1]

[1]*Uniklinik Freiburg - Allgemein- und Viszeralchirurgie, Freiburg im Breisgau, Deutschland*

**Background:**
Colorectal cancer (CRC) is a major contributor to global cancer-related mortality, particularly due to metastatic progression. Early-stage, non-metastasized CRC is largely curable, emphasizing the importance of comprehending metastasis for targeted therapeutic development. Current research predominantly centers on primary tumors, yet understanding CRC metastasis is crucial. The microbiome's role in primary CRC carcinogenesis is well-established, given the proximity to the gut microbiome and the breach of the epithelial barrier at the tumor site. Bacteria infiltrate primary tumors, shaping the Tumor Microenvironment (TME). Our prior work demonstrated tumor-induced reduction in antimicrobial defense, facilitating bacterial invasion. Despite the acknowledged impact of the gut microbiome on therapeutic responses, notably in immune checkpoint blockade therapy, the influence of bacteria on CRC metastasis remains inadequately elucidated. Advancing this knowledge is imperative for developing targeted metastasis-specific interventions.

**Materials and methods:**
In investigating the impact of the gut microbiome on CRC metastasis, we employed a surgical orthotopic organoid transplantation model for metastatic CRC. Within this mouse model, bacterial infiltration was validated in all metastatic sites—liver, lung, and peritoneum. Notably, the identified bacteria closely resembled those infiltrating the primary CRC, facilitated by a circulating tumor cell (CTC)-mediated intranuclear shuttle of bacteria to the metastatic site.

**Results:**
In liver metastases of this model, bacteria were identified, mirroring the composition of bacteria infiltrating the primary tumor. Notably, these bacteria were actively transported within CTCs from the primary tumor site to the liver. The study reveals that primary tumor-infiltrating bacteria are actively taken up by tumor cells becoming CTCs through caveolae-mediated endocytosis. Intriguingly, the bacteria were located inside the nucleus after endocytosis, with nucleolin playing a crucial role in this process.
This unique mechanism involves the recruitment of nucleolin to the plasma membrane upon bacterial contact, inducing the formation of a nucleolin/STAT1 complex, followed by nuclear transfer. Within the nucleus, bacteria may escape autophagy, lysosomal degradation, and immune detection, providing a safe niche within CTCs. The bacteria in CTCs induce STAT1-mediated secretion of pro-inflammatory cytokines and chemokines, promoting the formation of a pro-metastatic niche in the distant host organ. This niche is colonized by the intestinal microbiota and supports metastatic growth.
The translational aspect of the study, involving UICC stage IV CRC patients, demonstrates similar bacterial compositions in metastatic locations and primary tumors. Furthermore, bacterial persistence in matched locations is associated with a better prognosis. The findings suggest the potential development of bacteria and CTC-targeted therapies, especially for early-stage CRC patients, to prevent metastasis.

**Conclusion:**
In summary, our investigation employing a metastatic CRC model revealed a compelling connection between primary tumor-infiltrating bacteria and their presence in liver metastases. The bacteria, akin to those in the primary tumor, exhibited a unique mode of transport within CTCs. Notably, this transport involved active uptake through caveolae-mediated endocytosis, leading to the intriguing localization of bacteria within the nucleus of CTCs. Nucleolin emerged as a key player in this process, orchestrating the formation of a nucleolin/STAT1 complex that facilitated nuclear transfer.
Remarkably, this mechanism allowed bacteria to evade autophagy, lysosomal degradation, and immune detection, establishing a safe niche within CTCs. Once harbored in CTCs, bacteria induced STAT1-mediated secretion of pro-inflammatory cytokines and chemokines, fostering the creation of a pro-metastatic niche in distant host organs. This niche, colonized by the intestinal microbiota, provided favorable conditions for metastatic growth.
Furthermore, translational insights from UICC stage IV CRC patients unveiled similar bacterial compositions in metastatic locations and primary tumors. Notably, bacterial persistence in matched locations correlated with a better prognosis.
These findings advocate for the potential development of targeted therapies directed at bacteria and CTCs, particularly for early-stage CRC patients, with the aim of preventing metastasis.

**Single cell deconvolution of metastatic stage IV colorectal cancer**
ID: 344

**SCF - Oncology**

Luisa Schäfer[1], Bernhard Mauerer[1], Philipp Holzner[1], Rebecca Kesselring[1], Stefan Fichtner-Feigl[1], Christopher Berlin[1]

[1]*Uniklinik Freiburg - Allgemein- und Viszeralchirurgie, Freiburg im Breisgau, Deutschland*

**Background:**
Colorectal cancer (CRC) is the third most common cancer worldwide. Approximately 0.9 million people worldwide die from CRC each year. Metastasized CRC is associated with a poor prognosis and rapid disease progression. In stage IV CRC, peritoneal carcinomatosis is a major cause of death, along with liver metastases. Here, we studied the site-specific reconstitution of tumor cells and the corresponding tumor microenvironment in hepatic and peritoneal metastases of CRC in a sporadic organoid driven orthotopic CRC mouse model.

**Materials and methods:**
Murine colorectal tumor organoids, termed APTKA organoids, were surgically implanted beneath the cecum serosa. The organoids are devoid of *Apc*, *Tp53*, *Tgfbr2* and express constitutively active Kras (KrasG12D) and an activated/myristoylated isoform of AKT1. Following animal sacrifice, whole-tumor samples of primary tumor (PT), liver metastases (LM) and peritoneal carcinomatosis (PC) were analyzed by single-cell RNA sequencing. For analyses of infiltrative immune cells, single-cell suspensions from tissue samples were subjected to single-cell sorting.

**Results:**
Upon sacrifice, primary CRC growth rate was 98 %, liver and peritoneal metastases occurred in 67 % of all cases, respectively. The cellular composition of metastatic CRC (LM and PC) differs substantially from primary tumors. The tumor microenvironment (TME) of both metastatic tissues shows increased myeloid cell populations along with a decrease of B and T cell counts in LM and an almost complete exclusion in PC. Especially liver metastases show increased endothelial cell populations. In PC tissue, the amount of CAFs is elevated, with inflammatory altered CAFs (iCAFs) predominating. Furthermore, we found a massive deregulation of the stem cell compartment in both metastatic sites. In LM, the stem cell population is reduced to approximately one third compared to the primary tumor, whereas in PC tissue a more than twofold increase is detected. In addition, metabolic reprogramming is evident in metastatic CRC tissue. In stem cells of primary tumors, energy production mainly results from oxidative phosphorylation, whereas in LM glycolysis/gluconeogenesis is predominant and fatty acid metabolism ensures energetic capacity. Interestingly, coexpression analyses show overexpression of stem cell-supportive ligand-receptor pairs between CAFs and tumor cells in PC, leading to upregulation of stem cell supportive pathways. Compared with PT, the number of $CD8^{+}$ cytotoxic T cells is increased in metastatic tissue. However, analysis of their activity disclosed a dysfunctional, exhausted phenotype, especially in PC tissue, with a concurrent metabolic reprogramming. Additionally, differential pathway analysis confirms a downregulation of MHC Class II signaling pathways in metastatic CRC, implicating a reduction of MHC-II dependent antigen presentation, accompanied by increased PD-L1 pathway activation in PC.

**Conclusion:**
We identified cellular heterogeneity regarding tumor epithelium, stroma and immune cells in metastatic sites of CRC in comparison to the primary tumor. Interestingly, we found an intercellular site-specific network of cancer associated fibroblasts and tumor epithelium during peritoneal metastasis. We further demonstrated the dominance of the stem cell population in PC tissue, which preserves a self-promoting niche by stem cell supportive intercellular ligand-receptor signaling and increased fatty acid metabolism. Moreover, we deciphered a metastatic dysfunctional adaptive immunity by a loss of B cell dependent antigen presentation and consecutive effector T cell exhaustion. These findings significantly augment our comprehension of the intricate immune landscape in metastatic CRC, thereby laying a foundation for the development of future site-specific therapeutic approaches.

Abstracts – DCK 2024 – 141st Congress of the German Society of Surgery – Leipzig, April 24–26 • DOI 10.1515/iss-2024-2008
Innov Surg Sci 2024; 9 (Special Suppl 1): s303–s385

**The oncogenic transcription factor MYBL2 drives proliferation of myxoid/round cell liposarcoma**
ID: 357

**SCF - Oncology**

Julian Musa[1 2], Christine Sauerteig[3], Franziska Willis[1], Mohammed Al-Saeedi[3], Martin Schneider[1]

[1]*Department of General, Visceral, Thoracic, Transplantation, and Pediatric Surgery, University Hospital Giessen and Marburg, Campus Giessen, Deutschland*, [2]*Department of Translational Pediatric Sarcoma Research, German Cancer Research Center (DKFZ) Heidelberg, Deutschland*, [3]*Department of General, Visceral, and Transplantation Surgery, University Hospital Heidelberg, Deutschland*

**Background:**
Myxoid/round cell liposarcoma (MRCLS) are rare extremity-associated soft tissue sarcoma which are characterized by the pathognomonic driver oncogene FUS::DDIT3. In advanced tumor stages patient outcome is poor under current multimodal therapy, making development of novel prognostic markers and therapy strategies necessary to improve patient outcome. MYBL2 (alias B-MYB), a transcription factor known to regulate cell cycle progression and cell survival, is deregulated in many cancer entities, including sarcoma entities, and its high expression is associated with unfavorable patient outcome. Its role in MRCLS pathophysiology remains largely unclear.

**Materials and methods:**
RNA-sequencing of MRCLS cell lines, analysis of publicly available gene expression and clinical datasets (The Cancer Genome Atlas, TCGA and Genotype-Tissue Expression, GTEx), functional in vitro cell culture experiments using RNA interference (RNAi), qRT-PCR.

**Results:**
To evaluate MYBL2 gene expression levels in MRCLS, we analyzed RNA-sequencing data of two MRCLS cell lines (MLS-904 and MLS-1765). MYBL2 expression appears to be among the top 1% of most highly expressed genes in both MRCLS cell lines. Analyzing publicly available TCGA and GTEx data, MYBL2 is significantly overexpressed as compared to normal tissue and high MYBL2 expression is significantly associated with poor overall ($P < 0.001$) and recurrence-free patient survival ($P = 0.021$).
To obtain first functional insights into the role of MYBL2 in MRCLS, we performed a Gene Set Enrichment Analysis (GSEA) of MYBL2 co-expressed genes. Predominantly proliferation associated gene sets are positively and significantly enriched in MYBL2 co-expressed genes. RNAi-mediated knockdown of MYBL2 using two different MRCLS cell lines and four different siRNAs showed a strong reduction of proliferation in vitro without affecting cell survival.

**Conclusion:**
The transcription factor MYBL2 is overexpressed in MRCLS and regulates proliferation of MRCLS cell lines. MYBL2 might serve as a prognostic biomarker in MRCLS.
Further experiments to characterize the mechanism of MYBL2 upregulation, its mechanistic downstream effects as well as its potential role in sensitizing for specific targeted inhibitor treatments are currently ongoing.

**Tumor and stroma derived extracellular matrix components define pancreatic cancer metastasis**
ID: 414

**SCF - Oncology**

Lorena Llontop[1], Kim Honselmann Genannt Humme[1], Maren Dreckhan[1], Rüdiger Braun[1], Olha Lapshyna[1], Hauke Busch[2], Axel Künstner[2], Louisa Bolm[1], Meike Ten Winkel[1], Timo Gemoll[1], Thorben Sauer[1], Ulrich Wellner[1], Tobias Keck[1], Andrew Liss[3], Susanne Sebens[4]

[1]*Department of Surgery and Section for Translational Surgical Oncology and Biobanking, Ratzeburgerallee 160, Lübeck, Deutschland*, [2]*Institute for Systems Biology, Department of Dermatology, Ratzeburgerallee 160, Lübeck, Deutschland*, [3]*Pancreatic Biology Laboratory, Department of Surgery, 55 Fruit Street, Boston, United States*, [4]*Christian-Albrechts-Universität zu Kiel am Universitätsklinikum Schleswig-Holstein, Institut für Experimentelle Tumorforschung , Kiel, Deutschland*

**Background:**
Pancreatic ductal adenocarcinoma (PDAC) is the fourth most common cause of cancer death in industrial countries with less than 11% of patients surviving more than five years. When diagnosed, 50-60% of the patients already present with advanced disease and distant metastases with limited treatment options. One of the main characteristics of PDAC is its highly desmoplastic reaction which involves abundant amounts of extracellular matrix (ECM) in primary and metastatic tumors, making up almost 70-90% of the tumor volume. Understanding the biological role of these ECM components will be key to uncover new therapeutic targets in PDAC. However, the specific differences in ECM components between PDAC metastatic sites are not well known.

**Materials and methods:**
We performed statistical analysis on publicly available RNA expression profiles of PDAC primary and metastatic tumors (GSE71729). We retrieved microarray data from primary (n = 145), liver (n = 25), lung (n = 8), lymph node (n = 9), and peritoneal (n = 7) metastases. The expression values were analyzed using quantile normalization and differentially expressed genes were identified using limma. Genes were then ranked by fold-change in each tumor: peritoneum, lymph nodes, liver and lung, compared to control (primary). Gene set enrichment analysis (GSEA) was performed to identify transcriptional programs over-represented in metastatic sites compared to primary PDAC. We only considered gene sets significantly enriched that met a threshold of normalized enrichment score (NES) >1.5 or < −1.5 and false discovery rate (FDR) < 0.01. Next, only ECM genes uniquely and commonly expressed in metastatic tumors were further explored by ECM category (core matrisome proteins and matrisome-associated proteins) and subcategory (glycoproteins, collagens proteoglycans, ECM-affiliated proteins, ECM regulators and secreted factors). To validate the results, we analyzed RNA sequenced (by pair-end NextGen sequencing, Illumina) from primary (n = 5) and metastatic (liver, n = 6) PDAC patient-derived xenograft (PDX) mouse models. The resulting intensity files were demultiplexed with the bcl2fastq2 software and aligned to the human transcriptome using PICSES. Paired-end RNA-seq reads were aligned to the mouse mm10 and human hg19 reference genome assembly, obtained from the University of California at Santa Cruz (UCSC) using TopHat. Disambiguate was used to separate human and mice reads from the xenograft models. The reference annotations were quantified to determine their differential expression in known gene annotations and to obtain differentially expressed genes between various conditions. Expression values were normalized ($log_2$ TPMs) and a non-parametric Mann-Whitney U test was performed. Gene expression changes (> 1.3-fold or < -1.3-fold) were only considered if statistically significant (q < 0.01). A TPM value of 3 was used as a minimum threshold for expressed genes. Fold changes were calculated by the median difference of the -$log_2$ expression values. Species-specific differences in gene sequence allowed for the identification of genes expressed and changed in both the stroma (mouse) and cancer cells (human), separately.

**Results:**
Consistent with an important role of the ECM in PDAC biology, GSEA revealed multiple significantly regulated ECM-related gene sets between primary and metastatic sites in liver, lung, and lymph nodes but only one in peritoneal metastasis. Similar numbers of ECM genes were expressed in tumors originating from primary PDAC (n=416), peritoneum (n=377), lymph nodes (n=363), liver (n=353) and lung (n=350), with a great overlap of genes between all sites (317 common genes). However, ECM genes were highly differentially expressed (both, up and down) between primary and metastatic, showing higher variation of ECM components based on distance from the primary site sites (peritoneum 6% (23/377), lymph nodes 13% (49/363), liver 35% (124/353) and lung 43% (152/350), respectively). PDX analysis confirmed highly differentially expressed ECM genes between primary and liver metastasis (58% of all ECM genes). Interestingly, within the stroma (mouse sequences) most genes were down-regulated (57% down, 37% up), whereas in the cancer (human sequences) most of the genes were up-regulated (61% up, 23% down), alluding to distinct changes of ECM components in PDAC metastases.

**Conclusion:**
Exploratory extracellular matrix expression analysis in PDAC metastases suggests distinct alterations of ECM components between primary, peritoneal, lymph node, liver and lung metastases. ECM changes seem to be specific for each metastatic tumor site and cell origin in PDAC and allude to a multitude of novel therapeutic stromal and cancer specific targets in PDAC that need to be investigated in the future.

Abstracts – DCK 2024 – 141st Congress of the German Society of Surgery – Leipzig, April 24–26 • DOI 10.1515/iss-2024-2008
Innov Surg Sci 2024; 9 (Special Suppl 1): s303–s385

**Brg1 suppresses DEN-induced hepatocarcinogenesis in mice**
ID: 465

**SCF - Oncology**

Baocai Wang[1], Benedikt Kaufmann[1], Carolin Mogler[1], Jianye Wang[1], Mathias Schillmaier[1], Rupert Öllinger[1], Rickmer Braren[1], Roland Rad[1], Roland Schmid[1], Norbert Hueser[1], Helmut Friess[1], Guido von Figura[1], Daniel Hartmann[1]

[1]*Klinikum rechts der Isar der Technischen Universität München, München, Deutschland*

**Background:**
Hepatocellular carcinoma (HCC) is the most prevalent primary liver cancer. Mutations of SWItch/Sucrose NonFermentable (SWI/SNF) chromatin remodeling complex are commonly found in multiple tumors. Brahma-related gene 1 (Brg1) is a key member of the SWI/SNF family of proteins. However, the role of Brg1 in liver tumorigenesis and hepatocarcinogenesis remains unclear.

**Materials and methods:**
Liver tumors were induced in wild-type (Control) and hepatocyte-specific Brg1 knock out mice (Brg1 KO) by intraperitoneal diethylnitrosamine (DEN) injection. Tumorigenesis was analyzed after 11 months and molecular analyses were performed at different stages of tumorigenesis.

**Results:**
We showed that Brg1 expression is increased in tumor tissue compared to non-tumor tissue in DEN-induced HCC in wildtype mice. Liver tumor incidence, tumor numbers and tumor size were strongly increased in DEN-injected Brg1 KO mice compared to DEN-injected Control group, suggesting that Brg1 knockout contributes to tumor initiation in DEN-injected mice model. Besides, DEN-injected Brg1 KO mice had shorter survival compared to DEN-injected Control group.

**Conclusion:**
Our data reveal that deletion of Brg1 in hepatocytes promotes hepatocarcinogenesis in DEN-induced mice.

**Impact of different subtypes of obesity on CRC carcinogenesis in a mouse model**
ID: 507

**SCF - Oncology**

Luisa Schäfer[1], Bernhard Mauerer[1], Lisa Marx[1], Christopher Berlin[1], Goran Marjanovic[1], Rebecca Kesselring[1], Stefan Fichtner-Feigl[1], Claudia Lässle[1]

[1]*Uniklinik Freiburg - Allgemein- und Viszeralchirurgie, Freiburg im Breisgau, Deutschland*

**Background:**
Colorectal cancer (CRC) is the third most common cancer worldwide. Obesity is not only a major risk factor for the development of systemic diseases, moreover, obesity is also a significant risk factor for several types of cancer, including CRC. Stratification of different obesity subtypes and varying effects on carcinogenesis of CRC has not yet been done.

**Materials and methods:**
Two distinct obese mouse models were used, first a genetically induced (*ob/ob* mice) and second a high fat diet induced (C57BL/6J mice) obesity model. A total of 14 mice were studied, 7 *ob/ob* mice and 7 mice in the HFD group. After 6 weeks, murine CRC tumor organoids were implanted into the colon descendens via colonoscopy. After 4 weeks, animals were sacrificed, and tumor load was determined. Tumor tissue was further analyzed by flow cytometry. A stool sample was taken for microbiome determination.

**Results:**
Body weight is higher in *ob/ob* mice than in HFD mice. Regarding tumor burden, there is a considerable difference, *ob/ob* mice show a significant higher tumor burden than HFD mice. $CD8^+$ T-cells are significantly increased in the tumor microenvironment (TME) of HFD mice, whereas they are decreased in *ob/ob* mice. The two mouse models also show differences with respect to the microbiome. Respectively the Desulfovibrionales species are much higher in *ob/ob* mice than in the HFD microbiome, whereas Fusobacteriales and Lactobacillales are much higher in HFD mice.

**Conclusion:**
Both obesity mouse models showed tumor growth of CRC. However, the mice whose obesity is based on the leptin mutation as a genetic alteration (*ob/ob* mice) have a significantly higher tumor burden than the diet-induced obese mice (HFD mice). Consistent with this, HFD mice exhibit higher anti-tumoral immune cell infiltration.

Abstracts – DCK 2024 – 141st Congress of the German Society of Surgery – Leipzig, April 24–26 • DOI 10.1515/iss-2024-2008
Innov Surg Sci 2024; 9 (Special Suppl 1): s303–s385

**Uridine phosphorylase 1-associated metabolic reprogramming as a driver of immune evasion and progression of hepatocellular carcinoma**
ID: 570

**SCF - Oncology**

Pia Schuler[1], Gregor Ortmayr[1], Theresa Hillinger[1], Patrick Starlinger[2], Thomas Grüenberger[3], Doris Chen[4], Wolfgang Mikulits[1]

[1]*Center for Cancer Research, Comprehensive Cancer Center, Medical University of Vienna, Wien, Österreich*, [2]*Department of Surgery, Division of Hepatobiliary and Pancreatic Surgery, Mayo Clinic, Rochester, MN, United States*, [3]*Department of Surgery, HPB Center, Viennese Health Network, Clinic Favoriten and Sigmund Freud Private University, Vienna, Österreich*, [4]*Max Perutz Labs, Department of Chromosome Biology, Center for Integrative Bioinformatics Vienna, University of Vienna, Vienna, Österreich*

**Background:**
Only a subset of hepatocellular carcinoma (HCC) benefits from conventional immune checkpoint blockade (ICB). In the majority of patients, immune evasion or exclusion diminish response. Socalled immune-exhausted class of HCC is also associated with highly activated, aberrant TGF-beta signalling. In HCC, the receptor tyrosine kinase Axl cooperates with TGF-beta to cause aberrant, tumour-promoting TGF-beta signalling through phosphorylation of Smad3L-Ser213. We identified Uridine-phosphorylase 1 downstream of that collaboration. Interestingly, high expression is associated with adverse outcomes and immune evasion in several types of cancer. Recent data indicated further a prominent role in maintaining energy supply in cancer cells despite glucose scarcity. Considering a decline in oxygen and nutrient availability in the context of liver fibrosis and concomitant disturbance of hepatic microcirculation, UPP1 might grant additional metabolic flexibility favouring tumour cell survival. As established therapeutic strategies aim to restrict nutrient and oxygen supply, high UPP1 expression might limit the benefit of local ablative treatments like trans-arterial chemoembolization. Consequently, we aim to assess the role of UPP1 in altering tumour cell metabolism and how this might translate into the TME.

**Materials and methods:**
We used RNA-seq analysis of HCC models to identify Axl/TGF-beta targets. Modulation of Axl/TGF-beta activity and analyses in publicly available data sets of HCC patients were employed to verify target expression. Differences depending on varying metabolic conditions were assessed by Western Blot and qPCR. Genetic interference based on the CRISPR/CAS9 technology was utilised for functional studies in cellular HCC models.

**Results:**
RNA-seq analysis of HCC cells revealed uridine phosphorylase (UPP)1 as a promising target downstream of Axl/TGF-β collaboration since upregulation is associated with poor prognosis and immune evasion of HCC patients. Interference with Smad3L-Ser213 phosphorylation by inhibition of 14-3-3ζ or c-JNK confirmed UPP1 as a target of aberrant TGF-beta signalling. Genetic intervention with UPP1 reduced cell invasion and migration, while proliferation and survival of HCC cells remained unaffected. However, when kept in glucose-free medium supplemented by Uridine, only wildtype cells maintained proliferation. The addition of Uridine and corresponding nucleotides significantly increased the metabolic activity of HCC cell lines, indicating a utilisation as an alternate energy source. Interestingly UPP1 expression increased in the context of nutrient as well as oxygen deprivation.

**Conclusion:**
We identified UPP1 as a target of Axl-driven aberrant TGF-β signalling in HCC cells. In a nutrient-deprived TME, UPP1 upregulation plays an important role in HCC cell survival. This interesting link between metabolic escape and poor prognosis should be further investigated in relation to the impact

Abstracts – DCK 2024 – 141st Congress of the German Society of Surgery – Leipzig, April 24–26 • DOI 10.1515/iss-2024-2008
Innov Surg Sci 2024; 9 (Special Suppl 1): s303–s385

of immune cell infiltration and therapeutic options based on in vivo models and analyses in prospective HCC patient samples.

Abstracts – DCK 2024 – 141st Congress of the German Society of Surgery – Leipzig, April 24–26 • DOI 10.1515/iss-2024-2008
Innov Surg Sci 2024; 9 (Special Suppl 1): s303–s385

**Quantitative Assessment of histological Regression in Peritoneal carcinomatosis (QARP) as a novel scoring system after Pressurized Intra Peritoneal Aerosol Chemotherapy**
ID: 653

**SCF - Oncology**

Jan Philipp Ramspott[1], Mahmoud Abbas[2], Daniel Enrique Chourio Barboza[1], Alexander Bungert[1], Andreas Pascher[1], Eva Wardelmann[2], Judith C. Sporn[1]

[1]*Department of General, Visceral and Transplant Surgery, University Hospital Muenster, Muenster, Deutschland*, [2]*Gerhard-Domagk-Institute of Pathology, University Hospital Muenster, Muenster, Deutschland*

**Background:**
In gastrointestinal cancer patients peritoneal carcinomatosis is one of the leading causes of death. Pressurized Intra Peritoneal Aerosol Chemotherapy (PIPAC) is a promising treatment option for these patients. During PIPAC, a chemotherapeutic drug is applied to the abdominal cavity as an aerosol under pressure. One of the main challenges of PIPAC therapy remains the objective assessment of treatment response. Here, we present a novel scoring system to quantify treatment response after PIPAC based on histological regression: Quantitative Assessment of histological Regression in Peritoneal carcinomatosis (QARP).

**Materials and methods:**
Peritoneal carcinomatosis biopsies from 27 patients undergoing PIPAC were obtained and processed in a standardized fashion. According to our new five-tiered grading system QARP biopsies were scored as follows: grade 0: no residual tumor cells with regressive changes present; grade 1: 1 to 25% viable tumor cells per tumor focus with regressive changes present; grade 2: 26 to 50% viable tumor cells per tumor focus with regressive changes present; grade 3: 51 to 75% viable tumor cells per tumor focus with few regressive changes; grade 4: more than 75% viable tumor cells per tumor focus with minimal or no regressive changes. QARP scores were determined over time following each PIPAC cycle.

**Results:**
Patients median age was 62 years at the time of first PIPAC. Cancer origin was as follows: esophagogastric in eight patients (29.6%), colorectal in ten patients (37.0%), pancreatic in five patients (18.5%), biliary in three patients (11.1%), and appendiceal in two patients (7.4%). PIPAC with oxaliplatin was performed in colorectal cancer patients whereas all other primary cancers received doxorubicin based PIPAC. In total, 14 patients showed improvement in QARP scores or had stable low QARP scores and were labelled as “QARP responders” (QARP 0 or 1). Thirteen patients showed either stable high QARP scores (QARP 4) or worsening scores over time and were labelled as “QARP non-responders”. Higher QARP scores significantly correlated with higher PCI scores ($r=0.32$; $p=0.007$). No difference in overall survival was detected between QARP responders and QARP non-responders.

**Conclusion:**
Here we present our novel tool for microscopic quantification of treatment response after PIPAC therapy. Further studies are necessary to ascertain the reproducibility and prognostic significance of QARP to better identify these patients who benefits the most from intraperitoneal chemotherapy application.

Abstracts – DCK 2024 – 141st Congress of the German Society of Surgery – Leipzig, April 24–26 • DOI 10.1515/iss-2024-2008
Innov Surg Sci 2024; 9 (Special Suppl 1): s303–s385

**Scenario description as a complementary task for intraoperative mapping of language and visuospatial functions**
ID: 671

**SCF - Oncology**

Carla Tampier[1], Phillip Keil[1], Maura Luisa Becker[1], Roland Goldbrunner[1], Kristina Jonas[2], Carolin Weiß Lucas[1]

[1]*Klinik für Allgemeine Neurochirurgie, Uniklinik Köln*, [2]*Department Heilpädagogik und Rehabilitation, Universität zu Köln*

**Background:**
Picture and action naming are the most common tasks applied during awake surgery mapping, but are of limited use for longer test periods during the intraoperative monitoring phase. Moreover, due to the task's simplicity, more subtle language deficits such as syntax errors are easily overlooked. Spontaneous speech (e.g. with biographical triggers), however, is difficult to monitor and less controllable. In this study we developed and tested scenario drawings to serve as a more standardized stimulus material for language monitoring on the sentence level.

**Materials and methods:**
Twelve healthy subjects (6 women; 6 men) between 18-75 years of age were asked to describe black-and-white drawn pictures in two conditions, either in one sentence (A) or as detailed as possible with unlimited sentences (B). The pictures, either black and white (line pictures) or grey-scale colored (fill pictures), were then rated in a Likert scale (1-10) on “familiarity”, “recognisability”, “describability” and “emotions”. All sessions were performed via video-conference. The distinct task conditions were presented in pseudo-randomized order using a custom PsychoPy script. Groups were compared using Student's t-test. In addition, the aformentioned scenario pictures were used before and during awake glioma surgery to test the clinical feasibility.

**Results:**
In the comparison of line and fill pictures, “recognisability” was rated higher for fill pictures ($p=0.01$). The line pictures showed a trend towards a higher number of produced words and speaking time in both conditions A and B ($p<0.1$). When condition A was presented in the first block of testing, total word count and speaking time were increased compared to second block ($p<0.001$). Vice versa, in condition B, total word count and speaking time were increased when presented in the second testing block ($p<0.001$). In the pilot intraoperative application of the scenario descriptions in awake brain tumor surgery the pictures, especially the fill versions, showed to be feasible and very good accepted by the patients.

**Conclusion:**
Scenario description could be a useful complement to the existing awake surgery material. Due to the positive training effect on test performance of the unrestricted sentence description, a two-timed use (mapping with single sentence task and monitoring with unlimited sentence task) is conceivable. Here, usage of colored (fill) pictures is preferable with regard to recognisability.

Abstracts – DCK 2024 – 141st Congress of the German Society of Surgery – Leipzig, April 24–26 • DOI 10.1515/iss-2024-2008
Innov Surg Sci 2024; 9 (Special Suppl 1): s303–s385

**Multi-task fMRI-informed fibre tracking to guide awake monitoring strategies during brain tumour resection**
ID: 679

**SCF - Oncology**

Carolin Weiß Lucas[1], Ricardo Loução[1], Charlotte Nettekoven[1], Phillip Keil[1], Kristina Jonas[2], Maura Becker[1], Christoph Kabbasch[3], Jürgen Hampl[1], Marco Timmer[1], Roland Goldbrunner[1]

[1]*Center for Neurosurgery, University of Cologne, Faculty of Medicine and University Hospital Cologne, Köln, Deutschland*, [2]*Department of Special Education, Faculty of Human Sciences, University of Cologne, Köln, Deutschland*, [3]*Institute for Diagnostic and Interventional Radiology, University of Cologne, Faculty of Medicine and University Hospital Cologne, Köln, Deutschland*

**Background:**
The need of maximized brain tumour resection has to be balanced against the risk of functional damage. Lesion to important white matter connections is critical with regard to permanent deficits of essential physical and cognitive functions. We here present the clinical experience of using multi-task fMRI-informed tractography to plan the resection and intraoperative neuromonitoring of brain tumours in critical anatomical locations.

**Materials and methods:**
A consecutive series of 31 patients with intrinsic brain tumours in eloquent location were investigated preoperatively by 3T fMRI and diffusion imaging (dMRI). Depending on the tumour location, patients underwent an alternating movement task, and/or two language tasks: overt picture naming (PN) and forced choice (i.e., selection of a semantically related object) via left-handed button press based on the auditive presentation of a sentence. The fMRI activations were analysed in SPM 12 to define the starting/ending regions of interest (ROIs) for probabilistic tractography using FSL and MRTrix. Tracts identified in close neighbourhood of less than one cm from the tumour margins were classified “at risk”.

**Results:**
The exams were well tolerated and could be performed adequately by all patients. The results pointed towards a relatively robust identification of the major language tracts, i.e., ventral and dorsal stream components as well as the Frontal Aslant Tract (FAT). Clinical follow-up findings suggested a high risk of persistent communication deficits in patients with dorsal stream components (67% postoperative worsening, always persistent) or the FAT (67% postoperative worsening, persistent in half of the cases) at risk. In contrast, in our series, ventral stream components at risk were not associated with postoperative worsening, possibly due to the per se increased awareness and intraoperative tendency towards sparing likely essential structures.

**Conclusion:**
Multi-task fMRI-informed tractography could represent a powerful tool to optimize surgical decision making and intraoperative guidance. The results seem to highlight the need to expand the awake neuromonitoring strategies beyond ventral stream components such as directly “Broca” (inferior frontal gyrus)-related tracts to avoid prolonged communication deficits.

Abstracts – DCK 2024 – 141st Congress of the German Society of Surgery – Leipzig, April 24–26 • DOI 10.1515/iss-2024-2008
Innov Surg Sci 2024; 9 (Special Suppl 1): s303–s385

**KRASG12C Inhibitor-Based Combination Therapies for Pancreatic Cancer: Insights from Drug Screening**
ID: 695

**SCF - Oncology**

Constanza Tapia[1], Jonas Falke[1], Carolin Schneider[1], Lukas Krauß[1], Denise Müller[2], Engin Demirdizen[1], Melanie Spitzner[1], Tiago De Oliveira[1], Christian Schneeweis[3], Jochen Gaedcke[1 4], Silke Kaulfuß[4 5], Kimia Mirzakhani[4 5], Bernd Wollnik[4 5], Karly Conrads[4 6], Tim Beißbarth[4 6 7], Gabriela Salinas[4 8], Jonas Hügel[4 9], Nils Beyer[4 9], Sophia Reinländer[4 9], Ulrich Sax[4 9 10], Matthias Wirth[1 11], Lena Conradi[1 4 7], Maximillian Reichert[12 13 14 15 16], Volker Ellenrieder[4 7 17], Philipp Ströbel[2 4 7], Michael Ghadimi[1 7], Marian Grade[1 7], Dieter Saur[3 16], Elisabeth Hessmann[4 7 17], Günter Schneider[1 3 7]

[1]*University Medical Center Göttingen, Department of General, Visceral and Pediatric Surgery, Göttingen, Deutschland*, [2]*University Medical Center Göttingen, Institute of Pathology, Göttingen, Deutschland*, [3]*Technical University Munich, Institute for Translational Cancer Research and Experimental Cancer Therapy, Munich, Deutschland*, [4]*University Medical Center Göttingen, Clinical Research Unit 5002, KFO5002, Göttingen, Deutschland*, [5]*University Medical Center Göttingen, Institute of Human Genetics, Göttingen, Deutschland*, [6]*University Medical Center Göttingen, Department of Medical Bioinformatics, Göttingen, Deutschland*, [7]*CCC-N (Comprehensive Cancer Center Lower Saxony), Göttingen, Deutschland*, [8]*University Medical Center Göttingen, NGS Integrative Genomics Core Unit (NIG), Göttingen, Deutschland*, [9]*University Medical Center Göttingen, Department of Medical Informatics, Göttingen, Deutschland*, [10]*Campus-Institute Data Science (CIDAS), Göttingen, Deutschland*, [11]*Charité - Universitätsmedizin Berlin, Department of Hematology, Oncology and Cancer Immunology, Campus Benjamin Franklin, Berlin, Deutschland*, [12]*Technical University Munich, Medical Clinic and Polyclinic II, Klinikum rechts der Isar, Munich, Deutschland*, [13]*Technical University Munich, Translational Pancreatic Research Cancer Center, Medical Clinic and Polyclinic II, Klinikum Rechts Der Isar, Munich, Deutschland*, [14]*Technical University of Munich, Center for Protein Assemblies (CPA), Garching, Deutschland*, [15]*Technical University Munich, Center for Organoid Systems and Tissue Engineering (COS), Garching, Deutschland*, [16]*Technical University Munich, German Cancer Consortium (DKTK), partner site Munich, a partnership between DKFZ and University Hospital Klinikum rechts der Isar, Deutschland*, [17]*University Medical Center Göttingen, Department of Gastroenterology, Gastrointestinal Oncology and Endocrinology, Göttingen, Deutschland*

**Background:**
Pancreatic ductal adenocarcinoma (PDAC) is characterized by a high mortality rate and limited treatment options. Mutations in the KRAS oncogene drive the majority of PDAC cases, with $KRAS^{G12C}$ mutations present in a small subset. Specific KRASG12C inhibitors are showing promise in clinical trials; however, the mechanisms underlying the emergence of resistance and tumor adaptation are still insufficiently understood. Hence, the exploration of efficient pharmacological combination therapies is urgently needed. In this study, we conducted unbiased drug screening experiments to identify and validate $KRAS^{G12C}$ inhibitor-based combination therapies for PDAC.

**Materials and methods:**
Using $KRAS^{G12C}$-mutated MiaPaCa2 cells and patient-derived organoids (PDOs), we assessed the efficacy of Sotorasib, a $KRAS^{G12C}$ inhibitor. Cell viability was measured via CellTiter-Glo®, and $IC_{50}$ values were determined using GraphPad Prism. An unbiased drug screen with Sotorasib was performed on MiaPaCa2 cells, employing a library of 126 compounds. Dose-response curves were analyzed using the R package GRmetrics, and validation was conducted through clonogenic growth assays. Bliss synergy scores were computed using Synergy Finder software. Cell cycle progression was analyzed via propidium iodide (PI) staining and flow cytometry, and protein expression was evaluated using Western blot. Live cell analysis was performed on 2D cell lines and PDOs with Incucyte SX5®.

**Results:**
Sotorasib effectively targeted KRAS in 2D cell lines and PDOs as a single agent, inducing a cytostatic response with G1 phase cell cycle arrest. In the drug screen, several compounds targeting SOS1 (BI-3406, SHP2 (TNO-155), and broad-spectrum multi-kinase inhibitors (Nintedanib, Ponatinib), synergized with the KRASG12C inhibitor. These findings were validated in a novel $KRAS^{G12C}$-mutated patient-derived organoid model, and the synergism was further confirmed through clonogenic growth assays. Bliss synergy scores ranged from 10 to 30 after 7 days of treatment, highlighting the potential clinical significance of these combinations.

**Conclusion:**
This research contributes to developing more effective treatment strategies for PDAC and highlights the potential of Sotorasib-based combination therapies. The identified combination therapies involving KRASG12C inhibitors hold promise in improving the treatment outcomes for this challenging disease. Further mechanistic investigations are needed to fully understand the underpinnings of these synergistic effects. Altogether, these results provide valuable insights for guiding clinical trial design and the decisions made by molecular tumor boards, offering hope for enhanced therapeutic options in PDAC treatment.

**Patient-derived Xenografts Generated from Circulating Cancer Stem Cells in a 3D-in-vivo Model as a Novel Concept for Research and Clinical Decision-making in Pancreatic Cancer**
ID: 699

**SCF - Oncology**

Benedikt Wagner[1], Andreas Ettner-Sitter[2], Nicolas Ihlo[3], Merle Behr[3], Stefan Brunner[1], Florian Weber[4], Bettina Rau[5], Hans Jürgen Schlitt[1], Christoph Brochhausen[4], Dorothea Schott[6], Monika Pizon[6], Katharina Pachmann[6], Thiha Aung[2 7], Silke Härteis[2], Christina Hackl[8]

[1]*University Hospital Regensburg, Department of Surgery, Regensburg, Deutschland*, [2]*University of Regensburg, Institute for Molecular and Cellular Anatomy, Regensburg, Deutschland*, [3]*University of Regensburg, Faculty of Informatics and Data Science, Regensburg, Deutschland*, [4]*University of Regensburg, Institute of Pathology, Regensburg, Deutschland*, [5]*Hospital Neumarkt, Department of General, Visceral and Thoracic Surgery, Neumarkt in der Oberpfalz, Deutschland*, [6]*Laboratory Dr. Pachmann, Bayreuth, Deutschland*, [7]*Deggendorf Institute of Technology, Faculty of Applied Healthcare Science, Deggendorf, Deutschland*, [1]*University Hospital Regensburg, Department of Surgery, Regensburg, Deutschland*

**Background:**
Although significant improvements have been made in the treatment of pancreatic cancer in past years, the prognosis for this disease remains poor, with an overall 5-year survival rate of less than 10%. Therefore, new therapies and new preclinical models are needed. Circulating epithelial tumor cells (CETCs) represent a liquid equivalent of solid tumors. A rare subpopulation of CETCs, called circulating cancer stem cells (cCSCs), is responsible for recurrence and plays a key role in the metastatic cascade. Patient-derived xenografts (PDXs) are essential for oncology because they provide biologically relevant models for many cancer types and potential platforms for the development of treatment strategies for precision medicine. In this study, the development of patient-derived xenograft from cCSCs on the chorioallantoic membrane (CAM) was investigated.

**Materials and methods:**
The number of CETCs and tumorspheres was analyzed pre-, intra-, and postoperatively in 25 pancreatic cancer patients. For the identification of cCSCs, cell suspensions from peripheral blood were cultured in vitro under conditions favoring growth of tumorspheres. Furthermore, we used a rapid and efficient CAM culture system to generate PDXs from tumorspheres.

**Results:**
For the identification of cCSC from peripheral blood of pancreatic cancer patients, an innovative in vitro assay was established. The numbers of CETCs and tumorspheres was correlated with clinicopathological parameters like the completeness of resection (R-status) and the UICC stadium and showed higher numbers of CETCs and tumorspheres are associated with worse post-surgery UICC stadium. Application of tumorspheres successfully established PDXs within 7 days. These xenografts closely resemble the histological features of the primary tumor.

**Conclusion:**
The number of CETCs and tumorspheres is associated with some clinicopathological parameters. Applying the cCSCs onto the CAM membrane led to formation of tumors. Thus, the CAM model is a highly promising, inexpensive, and time-efficient model for generating PDXs using tumorspheres cultured from cCSCs. It also enables a novel model for testing new therapies for pancreatic cancer in a timely manner.
However, further studies are needed to evaluate the prognostic significance of cCSCs detection in pancreatic cancer.

Abstracts – DCK 2024 – 141st Congress of the German Society of Surgery – Leipzig, April 24–26 • DOI 10.1515/iss-2024-2008
Innov Surg Sci 2024; 9 (Special Suppl 1): s303–s385

**Unlocking chemosensitivity in gastric adenocarcinoma**
ID: 784

**SCF - Oncology**

Arianna Castagna[1], Can Yurttas[1], Frank-Jürgen Weinreich[1]

[1]*University Hospital Tübingen, Germany , Dept. of General and Transplant Surgery , Tübingen, Deutschland*

**Background:**
Late-stage gastric cancer (GC) is often associated with the development of peritoneal metastasis (PM), and the production of malignant ascites. However, the specific influence of ascites on tumor progression remains unclear. The interest in personalized ascites models is rising, intending to increase the understanding of metastasis-ascites interplay for better diagnostics and treatments. This *ex vivo* study compares cisplatin (CIS) and doxorubicin (DOX) HIPEC regimen, and examined how malignant ascites impacts GC grow.

**Materials and methods:**
The intestinal type GC cell line 23132/87 was taken in culture and allowed to adhere for 48h before addition of the testing solutions. The impedance generated by the cell layer was measured using an xCelligence RTCA SP device. The standard HIPEC drug regimens were studies, applying CIS or DOX alone, and CIS/DOX in combination. Further, patient derived ascites pure or diluted in physioneal was also examined. All experiments were analysed in triplicates. The impedance was measured in 15min intervals over 196h, computed and plotted using RTCA software version 1.2. Changes in impedance were provided as a dimensionless cell index (CI) normalized to the background impedance values.

**Results:**
We showed that it is feasible to create a basic cytotoxicity assay for GC 23132/87 using an xCELLigence RTCA SP. Kinetics and proliferation of cellular responses were real-time monitored. Combining CIS/DOX showed a superior cytotoxicity, if compared with CIS or DOX alone. Though, increasing the concentration of both drugs enhanced their effect on cell proliferation. Surprisingly, the addition of physioneal diluted ascites up to 90% showed a direct increase in cell proliferation, while the addition of 100% ascites led to a significant cytotoxic effect with impedance decrease.

**Conclusion:**
We established a reproducible and robust instrument-based cytotoxicity assay, which could offer information on GC chemosensitivity and on the relation ascites-tumor. The assay has been optimized for the GC 23132/87, but customized conditions can be applied for a broad range of different cell lines and cytotoxic compounds in order to establish personalised regimen for treating PM.

**Integrative molecular risk assessment of pre-malignant lesions on clinical resection samples for pancreatic neoplasia.**
ID: 793

**SCF - Oncology**

Matthäus Felsenstein[1], Cao Jin[1]

[1]*Charité Universitätsmedizin Berlin, Augustenberger Platz 1, Berlin*

**Background:**
Pancreatic Adenocarcinoma develops from non-invasive pre-malignant lesions. Cystic precursors, such as IPMN, are currently monitored via radiographic imaging, regularly causing delays in diagnosis or unnecessary treatments. Also from histological examination of resection margins, we are currently not able to determine, which lesions are at risk for malignant transformation. We employed snRNAseq and GeoMx spatial transcriptomics to correlate molecular data with histology, identifying high-risk markers for PDAC progression.

**Materials and methods:**
12 samples from 105 fresh frozen pancreatic resection specimen showed concomitant cystic precursor lesions (MCN, IPMN). We followed the 10x Genomics Single Cell Reagent Kit to process single nuclei from fresh frozen sections. Libraries were generated and sequenced on an Illumina NovaSeq XPlus platform. Fastq output files were pre-filtered using Cell Ranger, and clustered using Seurat pipeline (Rpackage). For spatial sequencing, regions of interest (ROI) were labelled containing dysplastic precursors and carcinoma with subsequent library preparation and sequencing.

**Results:**
Following single nuclei isolation and RNA extraction, we obtained moderate to high-quality RNA (RIN >4). Single nuclei data revealed significant intra- and interlesional heterogeneity. We identified cluster-specific markers and assigned them to known cell types. We executed the GeoMx workflow. Morphology markers identified dysplastic ducts (panCK), activated fibroblasts (aSMA), and sparse immune cells (CD45). Preliminary analyses revealed distinguishing clusters across carcinoma and the concomitant precursors.

**Conclusion:**
We integrated single-cell with spatial transcriptomics providing detailed insights into the molecular drivers for malignant transformation. These findings informs on clinical assessment of precursors and improves our understanding of precursor lesions at risk for carcinoma development.

**Role of the transcription factor Zeb1 in human pancreatic cancer-associated fibroblasts (CAFs)**
ID: 879

**SCF - Oncology**

Florian Krämer[1], Carmen Christoph[1], Christian Pilarsky[1], Robert Grützmann[2], Marc Stemmler[3], Nathalie Britzen-Laurent[1]

[1]*Translational Research Center, Chirurgische Klinik, Erlangen, Deutschland*, [2]*Chirurgische Klinik, Universitätsklinik Erlangen, Erlanngen, Deutschland*, [3]*Nikolaus-Fiebiger-Zentrum für molekulare Medizin, Erlangen, Deutschland*

**Background:**
Pancreatic ductal adenocarcinoma (PDAC) currently has an expected survival rate of approximately 6% over 5 years[1], mostly due to late diagnosis and the occurrence of (metachronous) metastasis.
In this context, the transcription factor ZEB1 plays a particularly important role. It has been identified and characterized as one of the major mediators of epithelial-mesenchymal-transition (EMT), a process which promotes invasion and metastasis and thus is associated with a poorer outcome in PDAC.[2]
Over the last few years, cancer associated fibroblasts (CAFs) have emerged as key regulators of disease progression. As yet, three different subpopulations of CAFs have been described in PDAC: inflammatory (i)CAFs, myofibroblast (my)CAFs and antigen-presenting (ap)CAFs. Each subpopulation interacts with tumor cells in its own way and thus modulates the microenvironment in PDAC.
Interestingly, the tumor microenvironment in pancreatic cancer is characterized by the coexistence of fibroblasts with different ZEB1 expression levels.

**Materials and methods:**
For our experiments we isolated cancer-associated fibroblasts (CAFs) and normal fibroblasts (NAFs) from tumor tissue. Primary cells with different ZEB1 expression levels were immortalised using hTERT-Transfection.
Differential gene expression between CAFs and NAFs and ZEB1 high/low expressing primary CAFs was investigated by RNA-sequencing, qPCR, Western Blot.
Additionally, we assessed the role of ZEB1 in immortalised CAFs by investigating its impact on proliferation, migration, and invasion as well as on CAF subtype differentiation using methods like Incucyte technology, wound healing and invasion assays as well as flow cytometry.

**Results:**
CAFs and NAFs displayed differences concerning ZEB1 expression. In addition, ZEB1 expression varied in CAFs and is associated with CAF subtypes, which might influence disease progression. Notably, it correlated with increased apCAF markers and with (partly) decreased myCAF and iCAF markers.
Furthermore, we observed that IFN-γ, which drives apCAF phenotype, was able to induce ZEB1 expression in CAFs.

**Conclusion:**
Our findings underscore the multifaceted impact of ZEB1 on PDAC progression. Further analysis involving a ZEB1-knockout cell line should investigate the interplay between ZEB1-expressing CAFs and tumor cells as well as the influence of ZEB1 on CAF subtype differentiation.

[1] McGuigan A, Kelly P, Turkington RC, Jones C, Coleman HG, McCain RS. Pancreatic cancer: A review of clinical diagnosis, epidemiology, treatment and outcomes.
[2] Krebs AM, Mitschke J, Lasierra Losada M, Schmalhofer O, Boerries M, Busch H, Boettcher M, Mougiakakos

D, Reichardt W, Bronsert P, Brunton VG, Pilarsky C, Winkler TH, Brabletz S, Stemmler MP, Brabletz T. The
EMT-activator Zeb1 is a key factor for cell plasticity and promotes metastasis in pancreatic cancer

**DPYD-polymorphism in rectal cancers (stages ≥ II) treated with upfront surgery and 5-FU-based postoperative chemotherapy – should we be more careful?**
ID: 898

**SCF - Oncology**

Paula Liersch[1 2], Sascha Dierks[3], Reiner Andag[3], Charlotte de Boer[4 5], Torsten Liersch[4], Birgit Jünemann[4], Andrea Hille[6], Julie Schanz[3 7]

[1]*Department of Haematology and Medical Oncology, University Medical Center of Göttingen, Germany*, [2]*Department of Preventive Dentistry, Periodontology and Cariology, University Medical Center of Göttingen, Germany*, [3]*Department of Clinical Chemistry, University Medical Center of Göttingen, Germany*, [4]*Department of General, Visceral and Pediatric Surgery, University Medical Center of Göttingen, Germany*, [5]*Department of Internal Medicine, Städtisches Klinikum Kiel, Germany*, [6]*Department of Radiotherapy and Radiation Oncology, University Medical Center of Göttingen, Germany*, [7]*Department of Haematology and Medical Oncology, University Medical Center of Göttingen, Germany, Göttingen, Deutschland*

**Background:**
In April 2020, the European Medicines Agency (EMA) recommended testing for the most common DPD-mutations, *DPYD**2A (c.1905+1G>A; IVS14+1G>A; rs3918290), *DPYD**13 (c.1679T>G; rs55886062), the polymorphism c.2846A>T (rs67376798) and Haplotype B3 (c.1236G>A; c.1129-5923C>G), in patients prior to 5-fluorouracil(5-FU)-based chemotherapy (CTx).
**Purpose:** Using blood samples of patients with upper rectal cancers (stages ≥ II; ≥ 12 cm above anal verge), *DPYD*-testing was performed to evaluate occult *DPYD*-polymorphism and its impact on CTx associated acute adverse events (AE), CTx dose reductions and long-term survival.

**Materials and methods:**
75 participants (median age: 69 yrs, f: 26, m: 49) of the GAST-05-phase-IIb-trial (ISRCTN35198481) had been tested for wildtype (WT) *DPYD**2A at staging and study entry. Upfront surgery was performed with partial (PME, n=41) or total (TME, n=34) mesorectal excision. Postoperatively, FOLFOX (4 cycles; FA: 400 mg/m², 5-FU: 2400 mg/m², OX: 100 mg/m²) was indicated for stages ≥ II. Acute AEs had to be recorded according to NCI-CTC-AE grading (vs 3.0). Survival was calculated using the Kaplan-Meier estimator, logrank-test as well as Cox proportional-hazards regression models. *DPYD*-re-testing was performed according to well-established methods blinded to clinical data.

**Results:**
*DPYD* re-testing revealed 100% WT for *DPYD**2A, *DPYD**13 and the polymorphism c.2846A>T. Interestingly, in 5 (10 %) patients receiving CTx a *DPYD*-Haplotype B3 status was detected. For 43 patients treated with FOLFOX, CTx-adherence (from cycle 1 to 4) was 100.0 %, 97.7 %, 95.3 % and 93.0 %, respectively. For patients with *DPYD*-WT, dosages of 5-FU and OX had been administered in 92.6 % and 75.3 %, and for Hap B3-patients in 68.8 % and 55.0 % of the target, respectively. In total of 612 AEs (all grades) with 28.9% hematologic, 27.6% gastrointestinal, 17.3% neurologic and 0.5 % cardiac events, AEs grade 3 / 4 had occurred in 3.3%. The relative risk (RR) for severe CTC-AEs (grades ≥ 3) was 2,43 in contrast to wildtype-patients. During follow-up (95 %CI: 62.1; 130.8 months), recurrences were diagnosed for *DPYD*-WT- and Hap B3-patients in 36.8 % and 80.0%, respectively. For WT patients, pairwise testing showed better DFS (p = 0.010), but not OS (p = 0.296). Multivariable regression analyses revealed patient`s age as significant parameter for DFS and OS.

**Conclusion:**
*DPYD*-re-testing in patients with upper LARC detected occult Hap B3 mutations. Its impact on treatment efficacy as well as survival remains unclear, but in clinical routine as well as in clinical trials investigators should be well aware about occult *DPYD*-polymorphism. To avoid severe AEs pretherapeutic testing is strongly recommended and should also be recognized by surgeons involved in multimodal treatment of patients with rectal cancer.

Abstracts – DCK 2024 – 141st Congress of the German Society of Surgery – Leipzig, April 24–26 • DOI 10.1515/iss-2024-2008
Innov Surg Sci 2024; 9 (Special Suppl 1): s303–s385

**Multiplex analysis of pancreatic, esophageal and rectal adenocarcinoma – a cross-cancer approach on the impact of neodjuvant therapy on the tumor microenvironment**
ID: 912

**SCF - Oncology**

Leonard Richter[1], Carmen Mota Reyes[1], Maximilian Kießler[1], Carolin Mogler[2], Ihsan Ekin Demir[1]

[1]*Klinikum rechts der Isar der Technischen Universität München, Klinik und Poliklinik für Chirurgie, München, Deutschland*, [2]*Institut für Pathologie, München, Deutschland*

**Background:**
The prognosis of gastrointestinal malignancies has improved significantly with the introduction of neoadjuvant therapy (neoTx). For rectal (RCa) and esophageal adenocarcinoma (OCa), neoTx has become the standard of care, resulting in downsizing of the tumor and significantly prolonged patient survival. However, the effects of neoTx on the topographical interactions between different populations of tumor-infiltrating immune cells, the degree of intratumoral immune infiltration, and the distance to tumor cells, as well as their spatial interactions with other important features of the tumor microenvironment (TME), are still unknown. In this cross-cancer histopathological profiling of the microenvironment of rectal and esophageal tumors, we aim to investigate the local effects of cytotoxic agents and determine whether these correspond to the pathological changes observed in pancreatic cancer (PCa).

**Materials and methods:**
In this study, we employed multiplex immunohistochemistry (mIHC) based on tyramide signal amplification (TSA) using OPAL® dyes and used a customized 6-plex approach to characterize tumor-associated stromal features (CD11c, CD34, NCAM, PGP9. 5, aSMA, PanCK) as well as tumor-infiltrating lymphoid (CD3, CD4, CD8, CD20, FOXP3, PanCK) and myeloid cell subsets (CD11b, CD33, CD68, CD208, HLA-DR, PanCK). FFPE samples from 50 neoadjuvantly treated RCa and 30 OCa patients and a matched cohort of primary resected patients were included in the analysis. Patients are matched among TNM, sex, age and for RCa the distance ab ano.

**Results:**
In PCa, neoTx alters the tumor microenvironment by depleting pro-tumorigenic immune cells, such as myeloid-derived suppressor cells (MDSCs) and regulatory T-cells, and suppressing tumor-associated stromal activation, neural invasion, and microangiogenesis. In our current study, we generated three standardized 6-plex panels for comprehensive histopathologic analysis of the immune architecture and associated stromal features of neoadjuvantly treated RCa and OCa patients compared with primary resected patients who underwent surgical resection with curative intent. Lymphoid subpopulations included Th-cells, cytotoxic T-cells, regulatory T-cells, and B-cells, while MDSCs, dendritic cells, M1- and M2-macrophages were analyzed in the myeloid panel within the same FFPE slide.

**Conclusion:**
Our findings highlight new differential cell identities in neoTx-treated PCa patients and confirm their suitability for optimizing future clinical immunotherapy trials. Our current approach validates multiplex IHC approaches for histological profiling of FFPE tumor samples in RCa and OCa and demonstrates the importance of a deep topographic characterization to understand the TME composition of gastrointestinal malignancies after neoTx.

Abstracts – DCK 2024 – 141st Congress of the German Society of Surgery – Leipzig, April 24–26 • DOI 10.1515/iss-2024-2008
Innov Surg Sci 2024; 9 (Special Suppl 1): s303–s385

**Continous and pulsed ultrasound therapy enhances liver organoid proliferation in vitro**
ID: 157

**SCF - Regeneration and Tissue Engineering**

Frederike Weber[1], Elyas Mirzaee Kakhki[1], Marc Roder[2], Jonas Negele[2], Claudia Blume[1], Lena Joppe[1], Sascha Kahlfuss[2], Maria Reich[3], Verena Keitel-Anselmino[3], Roland Croner[1], Cora Wex[1], Ulf Kahlert[1]

[1]*Magdeburg, Clinic for General-, Visceral-, Vascular and Transplant Surgery, Magdeburg, Deutschland*, [2]*Magdeburg, Institute of Molecular and Clinical Immunology, Magdeburg, Deutschland*, [3]*Magdeburg, Clinic for for Gastroenterology, Hepatology and Infectiology, Magdeburg, Deutschland*

**Background:**
Post-hepatectomy liver failure is a substantial complication after major and extended liver resection and associated with a high morbidity and mortality rate.[1] The clinical relevance of post-hepatectomy liver failure has increased as modern surgical techniques and expertise are increasingly leading to extended liver resections in previously inoperable patients.[1] These patients mostly received potentially hepatotoxic systemic therapy or suffer from chronic liver diseases, like steatosis. Hence, after resection the smaller remnant liver volume and impaired functional capacity leads to an increased risk of liver failure in these patients.[1] Therefore, novel techniques are needed to improve postsurgical outcome for these high-risk patients.

Recently, there has been a surge of interest in the concept of mechanotransduction and its induced proliferative and regenerative properties in several tissues.[2,3] In this approach, a mechanical stimulus like ultrasound propagates through the cells. By inducing vibration, various biological effects like membrane channel opening are triggered and consequently, transcriptional regulation occurs. This low intensity ultrasound induces molecular signals for cell proliferation, adhesion, migration, differentiation, and extracellular matrix production.[2-4] In tissues, ultrasound stimulation improves angiogenesis with additionally anti-inflammatory effects and attenuates metabolic dysfunction.[5-8] Regarding these promising results, we started to analyse the proliferative and regenerative response of the liver to this ultrasound technique for the first time.

**Materials and methods:**
We used low-intensity pulsed (LIPUS) and continous (LICUS) ultrasound stimulation of different intensities to stimulate patient-derived liver organoids at 3 consecutive days. These liver organoids mostly represent an adult tissue stem cell with cholangiocyte fate. For LIPUS we chose the intensities 100, 300, and 500 mW/cm$^2$ , whereas to limit the amount of ultrasound energy traveling through the organoids, lower intensities were required for the continous mode (100, 150, and 200 mW/cm$^2$). The organoids were then analysed for their proliferative response and apoptosis rate. Expression of adult stem cell markers like LGR5 and SOX9, together with cholangiocate marker CK19 were quantified using quantitative polymerase chain reaction (qPCR). Further metabolic changes in glucose (2NBDG) and lipid (cholesterol, LDL, $C_1$-$C_{12}$, $C_{16}$) uptake, together with mitochondrial metabolsim (MitoSox and MitoTracker) were analysed using fluorescence-acitvated cell sorting (FACS). Furthermore, organoid viability during nutritional starvation was evaluated in the control and ultrasound treated group using a resazurin based assay.

**Results:**
The proliferation rate of healthy liver organoids increased up to 5% for pulsed and up to 20% for continous ultrasound therapy, with simultanously low apoptosis rates. The expression of adult stem cell markers like LGR5 and SOX9 or the cholangiocyte marker CK19 are not altered between the control and treatment groups. Glucose uptake is not influenced by ultrasound therapy, whereas lipid particle uptake differs throughout the used therapy (pulsed or continous) and also throughout different intensities. Examplary, using LICUS at an intensity of 150 mW/cm$^2$ cholesterol uptake is increased, whereas the uptake of short chain fatty acids is reduced. Mitochondrial superoxide production and mitochondrial mass is reduced in both treatment groups of higher intensity. During nutritional starvation, organoids treated with LIPUS display a higher viability compared to the control for four consecutive days.

**Conclusion:**
Our study describes for the first time that continous and pulsed ultrasound therapy at different intensities enhance liver organoid proliferation *in vitro* with consecutive metabolic changes. Further transcriptional and functional analysis of ultrasound treated liver organoids are ongoing. This non-invasive and easily accessible approach has the potential to improve liver regeneration in high risk patients after major and extended hepatectomy and to reduce postsurgical morbidity and mortality.

**References:**

1. Rahbari NN, Garden OJ, Padbury R, et al. Posthepatectomy liver failure: a definition and grading by the International Study Group of Liver Surgery (ISGLS). *Surgery*. 2011 May;149(5):713-24. doi: 10.1016/j.surg.2010.10.001

2. Tan Y, Guo Y, Reed-Maldonado A, et al. Low-intensity pulsed ultrasound stimulates proliferation of stem/progenitor cells: what we need to know to translate basic science research into clinical applications. *Asian J Androl*. 2021;23(6):602. doi:10.4103/aja.aja_25_21
3. de Lucas B, Pérez LM, Bernal A, Gálvez BG. Ultrasound Therapy: Experiences and Perspectives for Regenerative Medicine. *Genes*. 2020;11(9):1086. doi:10.3390/genes11091086
4. Xia P, Shi Y, Wang X, Li X. Advances in the application of low-intensity pulsed ultrasound to mesenchymal stem cells. *Stem Cell Res Ther*. 2022;13(1):214. doi:10.1186/s13287-022-02887-z
5. Huang D, Gao Y, Wang S, et al. Impact of low-intensity pulsed ultrasound on transcription and metabolite compositions in proliferation and functionalization of human adipose-derived mesenchymal stromal cells. *Sci Rep*. 2020;10(1):13690. doi:10.1038/s41598-020-69430-z
6. Shindo T, Ito K, Ogata T, et al. Low-Intensity Pulsed Ultrasound Enhances Angiogenesis and Ameliorates Left Ventricular Dysfunction in a Mouse Model of Acute Myocardial Infarction. *Arterioscler Thromb Vasc Biol*. 2016;36(6):1220-1229. doi:10.1161/ATVBAHA.115.306477
7. Huerta TS, Devarajan A, Tsaava T, et al. Targeted peripheral focused ultrasound stimulation attenuates obesity-induced metabolic and inflammatory dysfunctions. *Sci Rep*. 2021;11(1):5083. doi:10.1038/s41598-021-84330-6
8. Tang L, Kang S, Yan W, et al. Low intensity pulsed ultrasound reduces liver inflammation caused by fatigue exercise. *J Acoust Soc Am*. 2023;153(2):1375-1385. doi:10.1121/10.0017355

**Imaging gastric cancer metastasis progression in an organotypic, three-dimensional functional model of the human peritoneum**
ID: 380

**SCF - Regeneration and Tissue Engineering**

Arianna Castagna[1 2], Frank-Jürgen Weinreich[1 2], Janine Spreuer[3], Kilian Wistuba-Hamprecht[3 4], Birgit Schittek[5], Marc André Reymond[2], Wiebke Solass[6 7]

[1]*University Hospital Tübingen, Germany , Dept. of General and Transplant Surgery, Tübingen, Deutschland*, [2]*University Hospital Tübingen, Germany, National Center for Pleura and Peritoneum , Tübingen, Deutschland*, [3]*University Hospital Tübingen, Germany, Section for Clinical Bioinformatics, Department of Internal Medicine I , Tübingen, Deutschland*, [4]*University Tübingen, Germany , Department of Immunology, Interfaculty Institute for Cell Biology , Tübingen, Deutschland*, [5]*University Hospital Tübingen, Germany, Dept. of Dermatology , Tübingen, Deutschland*, [6]*University Hospital Bern, Switzerland , Institute of Pathology , Bern, Schweiz*, [7]*University Hospital Tübingen, Germany, Institute of Pathology and Neuropathology , Tübingen, Deutschland*

**Background:**
Despite the introduction of multimodal treatment regimens, the prognosis of gastric cancer peritoneal metastasis (GCPM) remains poor. To establish more efficient therapies, a deeper understanding of pathophysiological mechanisms in the development of GCPM is necessary and this requires adequate functional models to test new therapeutics. Therefore, we established a three-dimensional human GCPM model to study tumor adhesion, invasion and growth.

**Materials and methods:**
A co-culture of peritoneal mesothelial cells with fibroblasts and collagen I was cultivated to further seed a human gastric cancer cell line (MKN45) on the surface. Different imaging techniques (optical microscopy, immunohistochemistry, scanning (SEM) and transmission (TEM) electron microscopy, as well as multiplex fluorescent microscopy served as tools to proof the sustainability of the model.

**Results:**
We showed that it is feasible to create a robust GCPM model. We showed that the model is reproducible under various conditions (6-, 12-, and 24-wells) and pre-analytical processing is possible. The imaging was feasible and allowed the comparison of morphological changes on the GCPM model to normal human peritoneum.

**Conclusion:**
We established a reproducible and robust organotypic model of GCPM which can be used to generate deeper knowledge on the pathophysiology of GCPM and might serve as a platform for testing different chemotherapy schemes in order to establish a personalized treatment of GCPM.

**Degenerative Cervical Myelopathy: Effects of Surgical Decompression on Functional Adaptation within the Somatosensory Cortex**
ID: 782

**SCF - Regeneration and Tissue Engineering**

Leonie Hams[1 2], Charlotte Nettekoven[1], Christoph Kabbasch[3], Anna Zdunczyk[4], Peter Vajkoczy[4], Roland Goldbrunner[1], Carolin Weiß Lucas[4]

[1]*University Hospital Cologne and Faculty of Medicine, Center of Neurosurgery, Köln, Deutschland*, [2]*University Hospital Cologne and Faculty of Medicine, Department of Neurology, Köln, Deutschland*, [3]*University Hospital Cologne and Faculty of Medicine, Institute for Diagnostic and Interventional Radiology, Köln, Deutschland*, [4]*Charité University Hospital, Department of Neurosurgery, Berlin, Deutschland*

**Background:**
Degenerative Cervical Myelopathy (DCM) is the most common cause of spinal cord dysfunction in adults. Despite extensive multimodal diagnostic possibilities, conclusive outcome predictors for surgical therapy are still lacking. Somatosensory deficits are a common early symptom of DCM. Here, the functional adaptation in the somatosensory system was investigated before and after surgical decompression using functional magnetic resonance imaging (fMRI).

**Materials and methods:**
The study involved 14 DCM patients (age 57 ± 15 years, 10 male) planned for surgical decompression and (until now) 11 age- and gender-matched control subjects (age 54 ± 15 years, 8 male). All participants underwent a clinical examination and a block design fMRI (3T) session consisting of alternating, passive somatosensory stimulation of the subjects' hands and feet. The presurgical blood oxygenation level dependent (BOLD) response was analyzed in MATLAB® 2019a and SPM12 at the group level using a three-way ANOVA with the factors “group” (patients/controls), “limb” (hand/foot) and “side” (left/right). For three patients which already completed follow-up examinations after 3, 9 and 24 months a single subject GLM analysis was performed addressing the postsurgical BOLD changes.

**Results:**
Preoperatively, the patient group featured reduced activation in the somatosensory cortex compared to the control group ($p \leq 0.001$, uncorrected). Also, the three patients with complete follow-up data individually revealed reduced BOLD response prior to surgery. One of those patients showed a continuous clinical improvement in the follow-up examinations (pre-op: JOA 10, 24 months post-op: JOA 17). This is consistent with an increased cortical response in the postoperative fMRI sessions, involving a larger volume of activation ($p \leq 0.05$, FWE corrected) in the somatosensory cortex than at baseline. The other two patients with only mild clinical impairment (pre-op: JOA 14 and 16.5) neither showed a relevant clinical improvement nor an increase of cortical response.

**Conclusion:**
DCM patients showed reduced cortical responsiveness to somatosensory stimuli preoperatively. In addition, it was shown on a single patient level that BOLD responses might increase in parallel with clinical improvement after surgery. In other clinically less impaired patients, cortical activation and clinical status remained reduced. This points towards a differential functional network responsiveness to surgery, dependent on individual factors such as the severity of preoperative symptoms.

**Heparan sulfate fine-tuned interleukin (IL)-1 signaling inhibits insulin secretion of grafted pancreatic islets**
ID: 96

**SCF - Transplantation**

Selina Wrublewsky[1], Sandra Rother[2], Franziska Pohlemann[1], Toni Radanovic[2], Fabian Junker[2], Anne S. Boewe[1], Stefan Schunk[3], Leticia P. Roma[4], Gloria Ruiz-Gómez[5], Teresa M. Pisabarro[5], Patrick E. Macdonald[6], Michael D. Menger[1], Matthias W. Laschke[1], Emmanuel Ampofo[1]

[1]*Institute for Clinical and Experimental Surgery, Saarland University*, [2]*Center for Molecular Signaling, Präklinisches Zentrum für Molekulare Signalverarbeitung (PZMS), Saarland University*, [3]*Department of Internal Medicine IV (Nephrology and Hypertension) and Translational Cardio-Renal Medicine, Saarland University*, [4]*Biophysics Department, Center for Human and Molecular Biology, Saarland University*, [5]*Structural Bioinformatics, BIOTEC TU Dresden*, [6]*Department of Pharmacology and Alberta Diabetes Institute, University of Alberta*

**Background:**
Inhibition of interleukin (IL)-1 signaling has been shown to improve the outcome of clinical islet transplantation. However, the mode of action of IL-1α and IL-1β during this process is still elusive. Therefore, the aim of the present study was to investigate (i) the cellular source of IL-1α and IL-1β within the islets, (ii) the role of IL-1 signaling for the endocrine function of β-cells and (iii) the effect of IL-1α and IL-1β on islet transplantation.

**Materials and methods:**
Islets were isolated from wild type (WT), IL-1α$^{-/-}$ and IL-1β$^{-/-}$ donor mice and exposed to IL-1α or IL-1β under hypoxic conditions for 24 h. The viability and cellular composition of these islets were determined by using flow cytometry, calcein/propidium iodide staining and immunohistochemistry. Secretion of insulin, IL-1α and IL-1β was analyzed by an enzyme-linked immunosorbent assay (ELISA). Expression of insulin, MafA and pancreatic duodenal homeobox protein (PDX)-1 was analyzed by quantitative real-time (qRT)-PCR and Western blot. Binding of IL-1α and IL-1β to heparan sulfate of islets was analyzed by surface-plasmon resonance (SPR) spectroscopy. The molecular recognition of HEP by IL-1α was investigated through molecular modeling. *In vivo*, WT, IL-1α$^{-/-}$ and IL-1β$^{-/-}$ islets were transplanted into WT mouse dorsal skinfold chambers and their revascularization was assessed by intravital fluorescence microscopy. Moreover, we used the streptozotocin-induced diabetic mouse model to study the endocrine function of the grafts.

**Results:**
We found that IL-1α and IL-1β are solely expressed by islet resident macrophages and both cytokines reduce insulin secretion. Additional analyses revealed that only IL-1α is capable of binding to heparan sulfate of islets, which, in turn, diminished the inhibitory effect of IL-1α on insulin secretion. Through molecular modeling two main recognition sites of tetrameric and hexameric heparan sulfate on IL-1α were predicted. The analysis of the underlying mechanism for the decreased insulin secretion demonstrated that the loss of both cytokines increases insulin expression in a PDX-1- and MafA-dependent manner. By using the mouse dorsal skinfold chamber model, we found an improved insulin-driven revascularization and reduced immune cell infiltration of transplanted IL-1α$^{-/-}$ and IL-1β$^{-/-}$ islets when compared to controls. Accordingly, the transplantation of IL-1α$^{-/-}$ and IL-1β$^{-/-}$ islets under the kidney capsule of diabetic mice led to an accelerated restoration of normoglycemia.

**Conclusion:**
With the binding of IL-1a to heparan sulfate, we were not only able to identify a completely new regulatory mechanism, but we also demonstrated that the loss of IL-1α or IL-1β improves the revascularization, and thus, the engraftment of islets. Hence, the herein introduced anti-inflammatory approach represents a promising strategy to increase the success rates of clinical islet transplantation.

**Aging is transferable: Old Organs Accelerate Aging and Induce Senescence in Young Recipients**
ID: 135

**SCF - Transplantation**

Friederike Martin[1 2], Jasper Iske[3], Maximilian Rösel[3], Andreas Schröter[4], Merih Gizlenci[1], Igor Maximilian Sauer[2], Johann Pratschke[2], Hao Zhou[1], Stefan G. Tullius[1]

[1]*Division of Transplant Surgery, Department of Surgery, Brigham and Women's Hospital, Harvard Medical School, Boston MA, United States*, [2]*Chirurgische Klinik | Charité Campus Virchow-Klinikum, Charité Universitätsmedizin Berlin, Berlin, Deutschland*, [3]*Klinik für Herz-, Thorax-, und Gefäßchirurgie, Deutsches Herzzentrum der Charité, Berlin, Deutschland*, [4]*Regenerative Medicine and Experimental Surgery, Department of General, Visceral and Transplant Surgery, Medizinische Hochschule Hannover, Hannover, Deutschland*

**Background:**
The utilization of old organs has increased steadily because of a growing life expectancy and an ever-increasing shortage of organs for transplantation. Aging is associated with the accumulation of senescent cells that produce a characteristic set of pro-inflammatory markers, the so-called senescent-associated secretory phenotype (SASP). Here, we ask whether heterochronic (age-mismatched) transplants of old organs accelerate aging and induce senescence in young recipients. Mechanistic studies included the transfer of cell-free mtDNA (cf-mtDNA) as a characteristic product of senescent cells and the depletion of senescent cells prior to Tx.

**Materials and methods:**
Young or old isogeneic hearts (C57BL/6; 3 and 18+ months) were transplanted into young and middle-aged recipients (3 and 12months). Recipients underwent sequential physical and neurobehavioral tests over 5 months. SASP factors, including mtDNA and senescent cell accumulation were measured in recipient tissues (lymph nodes, brain, muscle, liver, adipose tissue) by rtPCR, RNA *in situ* hybridization, cytokine-analysis, immunofluorescence staining, and immunohistochemistry; plasma components were analyzed by RNA *in situ* hybridization, and cytokine-analysis. Delineating mechanisms that drive senescence transfer, we injected C57BL/6 mice with mtDNA to imitate the effects of heterochronic Tx. Senolytics (Dasatinib & Quercetin) were applied to define the effects of depleting senescent cells prior to organ procurement.

**Results:**
mtDNA levels were significantly elevated in young and middle-aged recipients immediately after the transplantation of old organs when compared to levels in recipients of young organs ($p \leq 0.01$ by 24 hrs). Quantifying the burden of senescent cells in non-transplant tissues (livers, drainage lymph nodes, and adipose tissue) 30 days after heterochronic cardiac Tx revealed increased numbers of senescent cells with the characteristic expression of $p21^{Cip1}$ and $p16^{Ink4a}$ (20.3-fold increase in liver, $p \leq 0.0001$; 3.1-fold in drainage LNs, $p \leq 0.01$; 1.8-fold in adipose tissue, $p \leq 0.05$).
Physical and neurobehavioral tests by 1 and 4 months showed compromised capacities for recipients of old compared to young hearts (Rotarod, minimum grip strength, and locomotor activity test $p \leq 0.01$, $p \leq 0.0001$, and $p \leq 0.01$ after 1 month; Novelty-Y-maze $p \leq 0.01$ after 4 months).
Mechanistically, mtDNA injections, imitating a possible pathway of senescence transfer, led to increased levels of $p21^{Cip1}$ and proinflammatory SASP factors in brains of young recipients ($p21^{Cip1}$, IL-6, and TNFα, all $p \leq 0.05$). Moreover, physical capacities were compromised in recipients after the injection of mtDNA. The role of senescent cells and their SASP product as the culprit of transferring senescence was confirmed with the treatment of senolytics that depleted senescent cells, decreased mtDNA levels after transplantation and improved physical capacities in recipients of old heart (Rotarod $p \leq 0.01$).

**Conclusion:**

Heterochronic (old to young) organ transplants accelerate senescence in recipient animals, leading to compromised physical and cognitive capacities. The accumulation of cf-mtDNA plays a critical role in accelerated aging while the depletion of senescent cells with senolytics prevents the hastened aging process. Those preclinical findings are of potential critical relevance for clinical transplantation.

Abstracts – DCK 2024 – 141st Congress of the German Society of Surgery – Leipzig, April 24–26 • DOI 10.1515/iss-2024-2008
Innov Surg Sci 2024; 9 (Special Suppl 1): s303–s385

**Absent in melanoma (AIM)2 is crucially involved in the revascularization and endocrine function of transplanted pancreatic islets**
ID: 174

**SCF - Transplantation**

Cedric Wilden[1], Selina Wrublewsky[1], Michael D. Menger[1], Matthias W. Laschke[1], Emmanuel Ampofo[1]

[1]*Institute for Clinical & Experimental Surgery, Saarland University, Homburg, Deutschland*

**Background:**
Inflammation-induced cell death, which is caused by an insufficient revascularization of the grafts, is a major problem of pancreatic islet transplantation. The inflammasome, absent in melanoma (AIM)2, promotes the maturation and release of cytokines, and thus triggers the inflammatory response. However, the function of AIM2 in pancreatic islets is still elusive. Therefore, we analysed whether this inflammasome affects the revascularization and endocrine function of transplanted islets.

**Materials and methods:**
Islets were isolated from wild type (WT) and *AIM2*$^{-/-}$ mice and cultured for 24 hours. The cellular composition of the islets was analyzed by immunohistochemistry. Insulin secretion was determined by an enzyme-linked immunosorbent assay (ELISA) and insulin expression by quantitative real-time (qRT)-PCR. The regulatory mechanism of the decreased insulin secretion after loss of AIM2 was deciphered by Western blot. In vivo, islets were transplanted into mouse dorsal skinfold chambers and their revascularization was assessed by intravital fluorescence microscopy. Moreover, we used the streptozotocin-induced diabetic mouse model for evaluating the endocrine function of the grafts.

**Results:**
We could demonstrate that loss of AIM2 did not affect the viability and cellular composition of isolated islets. However, we detected a decreased glucose-stimulated insulin secretion in *AIM2*$^{-/-}$ islets. The analyses of the underlying mechanism revealed that *AIM2* deficiency increases the protein level of p202, a transcriptional regulator of interferon (IFN)-β and IFN-γ gene expression. Of note, we found that both cytokines exert inhibitory action on insulin gene expression. In vivo, transplanted *AIM2*$^{-/-}$ islets exhibited a smaller revascularized area and lower functional microvessel density when compared to controls. Furthermore, the transplantation of *AIM2*$^{-/-}$ islets under the kidney capsule of diabetic mice did not restore physiological blood glucose levels.

**Conclusion:**
Taken together, the present study provides the first evidence that AIM2 is of major importance for the adequate endocrine function and revascularization of transplanted islets by inhibition of IFN-β and IFN-γ expression.

**Elderly liver grafts are more susceptible to ischemia-reperfusion after long cold ischemia than grafts from young donors – an ex vivo liver machine perfusion analysis in Sprague Dawley Rats**
ID: 696

**SCF - Transplantation**

Leonard Boerger[1], Kilian Walter[1], Anna Pietsch[1], Luca Padoan[1], Karl Hillebrandt[1 2], Joseph Gassner[1 2], Felix Krenzien[1 2], Johann Pratschke[1], Igor Maximilian Sauer[1], Nathanael Raschzok[1 2], Simon Moosburner[1 2]

[1]*Department of Surgery, Campus Charité Mitte* | *Campus Virchow-Klinikum, Experimental Surgery, Charité - Universitätsmedizin Berlin, Berlin, Deutschland*, [2]*Berlin Institute of Health at Charité - Universitätsmedizin Berlin, BIH Academy, Clinician Scientist Program, Berlin, Deutschland*

**Background:**
Organ shortage is an acute and ongoing issue in liver transplantation worldwide. One measure to alleviate this crisis, especially in countries without an option for Donation after Cardiac Death (DCD), has been the consideration of grafts from so-called extended criteria donors (ECD). One of the ECD factors is donor age > 65 years. Considering current demographic trends, the population of elderly donors has great potential to expand the donor pool. However, new challenges arrive when utilizing liver grafts from elderly donors, as recipient have inferior reported outcomes. Normothermic ex vivo liver machine perfusion (NMP) has recently been proposed as a technique to increase graft viability and assess graft quality prior to transplantation. Because NMP has not yet been widely implemented in clinical practice, the gold standard of organ preservation, for most of the world, is still static cold storage (SCS). The length of cold ischemia time (CIT) in the period between organ procurement and transplantation is a crucial factor for organ damage. We here aimed to characterize young and elderly liver grafts after prolonged SCS using a previously published standardized rat model of NMP and wanted to investigate the effects of a combined damage model of donor age and long CIT on graft viability and quality.

**Materials and methods:**
A total of 24 livers from Sprague Dawley rats aged 3 months or 12 months (3M or 12M) were procured. The portal vein, common hepatic artery and bile duct were isolated and cannulated during microsurgery. After flushing with cold histidine-tryptophan-ketoglutarate (HTK) solution via both vessels, livers from each group were randomly distributed into two subgroups with either 6 or 12h of CIT (6h CIT, 12h CIT). After static cold storage, all grafts were flushed with cold saline and perfused for 6 hours using a proprietary dual vessel NMP system (n=6/group). Produced bile was continuously collected. Perfusate and tissue samples were taken for histological, biochemical analyses, and blood gas analyses throughout perfusion.

**Results:**
Livers of older animals were heavier (20.70g vs. 17.45g; p=0.021). Arterial and portal venous perfusion pressures frequently rose above the respective reference ranges and required pharmacological intervention, regardless of age or length of CIT. Comparison showed no significant difference between the groups regarding perfusion pressures. Livers in all groups cleared lactate with a peak of 37mg/dl to 8mg/dl. Alanine-aminotransferase levels rose slightly up to a maximum of 486 U/l (IQR 297, 705) across groups, with the highest liver damage seen in livers from older donors with prolonged cold ischemia time (12M | 12h CIT; 751 U/l, IQR 644, 887; p=0.039). Overall cellular damage, as measured by lactate-dehydrogenase was equally significantly elevated (12M | 12h CIT 8659 U/l vs. 4125 U/l 3M | 12h CIT, p=0.029). Bile was produced continuously throughout the perfusion and peaked after two hours with 835mg/h (IQR 660,912). The median bile production did not differ between intervention groups (after 6 hours p=0.8). H&E staining showed vital liver tissue after 6 hours of NMP, with a tendency towards more sinusoidal dilatation and slight necrosis in grafts from longer cold ischemia or elderly donors. Energy reserves depleted faster in grafts after longer CIT with glucose levels being significantly lower (12M | 12h CIT 148mg/dl vs. 12M| 6h CIT 236mg/dl; p<0.001).

**Conclusion:**
Overall damage increased incrementally by prolonged CIT, but was exacerbated in grafts from older donors which had longer CIT. We assume this correlation between age, CIT, and liver damage to be associated with an increased susceptibility of older grafts to ischemia-reperfusion injury. Further analyses into activation of inflammatory pathways, especially in older grafts and existing regulatory mechanisms are required.

**Macrophage colony stimulating factor (CSF1) promotes liver regeneration during normothermic machine perfusion (NMP)**
ID: 759

**SCF - Transplantation**

Sarah Schulze[1 2], Katharine Irvine[2], Nick Butler[3], Sahar Keshvari[2]

[1]*Technische Universität München, Klinikum rechts der Isar, München, Deutschland*, [2]*University of Queensland, Mater Research, Translational Research Institute, Brisbane, Australia*, [3]*University of Queensland, Princess Alexandra Hospital, Brisbane, Australia*

**Background:**
Ex-vivo liver normothermic machine perfusion (NMP) is a promising solution for preserving liver grafts from extended criteria donors, addressing the shortage of donor organs. It permits assessment of hepatocellular and biliary viability before transplantation and has become an established procedure in transplant centres. NMP not only assesses organ quality but also enables the investigation of new therapies by facilitating drug administration during perfusion. We have shown in preclinical models that stimulating macrophages with the growth factor Colony Stimulating Factor 1 (CSF1) promotes liver regeneration and resolution of advanced fibrosis[1] and steatosis (unpublished) in mice, and liver ischaemia-reperfusion injury in rats (unpublished). This study aimed to investigate the peri-surgical regulation of circulating CSF1 in patients undergoing liver resection or transplantation, and to determine the impact of CSF1 on human livers during normothermic machine perfusion (NMP).

**Materials and methods:**
Using the normothermic machine perfusion (NMP) we analyzed the role of CSF1 on liver preservation by administrating it to the perfusion circuit. Oxygenated NMP has been proposed as a technique to prolong the window of opportunity for transplantation and assess organ viability. We measured circulating CSF1 during liver resections, liver transplantation and NMP up to 24h. We analyzed blood monocytes, liver macrophages, organ viabilty and cytokine signalling.

**Results:**
We show that the administered circulating CSF1 is rapidly consumed by the perfused liver during NMP preservation depending on the donor organ quality. As macrophages in the donor organ are essential for liver regeneration, providing the main source of hepatocyte growth factors, we demonstrate histological analyses of continuous biopsy samples during NMP. We show cell proliferation (Ki67) and macrophage cell infiltration (CD68), which may reflect a change in tissue macrophage morphology and monocyte recruitment from the perfusate. Cytokines, including IL-10, were elevated in the perfusate at different time points post treatment, suggesting that a pro-inflammatory and anti-apoptotic milieu, induced by macrophage stimulation, results in augmented liver regeneration. During reperfusion we found variable levels of CSF1 in the recipient’s blood circulation. Interestingly, post-operative CSF1 levels seem to differ from the peri-surgical concentrations, suggesting an ongoing pro-regenerative effect during liver regeneration several days after transplantation.

**Conclusion:**
It is the first time that direct CSF1 effect on the human liver has been tested. Given the positive effects of acute CSF1 treatment on macrophages and regeneration, we suggest that the pro-inflammatory milieu created by CSF1-dependent macrophages contributes to post-surgical liver regeneration, and that CSF1 could be a valuable multi-faceted therapy in peri-surgical settings in patients. By alleviating

[1] Keshvari, S. et al. Therapeutic potential of macrophage colony-stimulating factor in chronic liver disease. Disease models & mechanisms 15, doi:10.1242/dmm.049387 (2022).

the impact of ischemic injury and preserving liver function, it could increase availability of organs for transplant, allowing marginal donor grafts to be transplanted in the future.

**The Complex Interactions of Pain, Physical Activity, and Mental Health in Lipedema Patients and The Role of Pain Reduction via Liposuction.**
ID: 80

**SCF - Varia**

Marie-Luise Aitzetmüller-Klietz[1], Lena Busch[1], Matthias Hamatschek[1], Matthias Paul[2], Carsten Schriek[3], Philipp Wiebringhaus[1], Matthias Aitzetmüller-Klietz[1], Maximilian Kückelhaus[1], Britta Pehlke[1], Tobias Hirsch[1]

[1]*Plastic and Reconstructive Surgery, Institute of Musculoskeletal Medicine, Muenster, Deutschland*, [2]*University Hospital Münster, Department of Cardiovascular Medicine, Division Steinfurt, Steinfurt, Deutschland*, [3]*Academy for Diagnostics and Prevention, Münster, Deutschland*

**Background:**
Lipedema is characterized by large accumulations of subcutaneous fat with a prevalence of 5-15% in women, primarily affecting the legs and limbs. Lipedemic tissue is characterized by pain, sensitivity to pressure, and feeling of tension [1], [2]. The painful tissue can cause impairment in mobility and the possibility to engage in physical activity. Also, lipedema patients often suffer from comorbid adiposities, accentuating the high relevance to enhance weight loss [3], [4]. Lipedema can lead to impairment in mobility and psychosocial problems and patients suffering from lipedema report significant impairment on their quality of life (QoL) and higher levels of depression [5]. Meanwhile, physical activity is an important influencing factor for mental health and the support of physical activity represents a high potential option for effective treatment of depressive disorders [6]. As pain in patients suffering from lipedema stand in a complex interaction with physical activity and mental health, it was the aim of this study to gain more profound insight into such differentiated interrelations.

**Materials and methods:**
We included $n$=511 patients diagnosed with lipedema with a mean age of $M$=40.16 (±12.45) years and a mean BMI of $M$=33.86 (±7.80) kg/m$^2$. Mental health was measured via two questionnaires: Depression severity was measured via PHQ-9 [7] (nine items on a four-point Likert scale). QoL was measured via WHOQOL-BREF [8] (26 items on a six-point Likert scale) with the subscales mental, physical, social, environmental, and overall health. Pain in the referring areas was measured via a single-item questionnaire (ten-point Likert scale). Physical activity was measured via the number of hours per week assessed on seven dimensions (gym, nordic walking, swimming, biking, running, others, and none). In statistical analysis, a Response Surface Analysis (RSA) was conducted. RSA is based on polynomial regression analysis and advances traditional regression analyses as differentiated effects of the two predictor variables on the outcome variable can be analyzed depending on the degree of their congruence [9]. In the analysis, physical activity and pain were used as independent variables and scales measuring depression severity and QoL were used as dependent variables. Data analysis was conducted via the programming language R.

**Results:**
Mean depression severity measured via PHQ-9 was $M$=10.84 (±6.39) indicating moderate depression severity. In preliminary moderator analyses, the stage of lipedema had no effect on the regression of the outcome variables on pain and physical activity for analyses ($p$>0.005). Explained variance was high for the model predicting depression severity ($R^2$=0.18, $p$<0.001) and physical health ($R^2$=0.30, $p$<0.001) and moderate for overall health ($R^2$=0.12, $p$<0.001). Pain had a strong positive main effect in the analysis for depression severity and a strong negative main effect on mental, physical, and overall health (all $p$<0.01). Physical activity had a strong negative main effect in the analysis on depression severity ($p$<0.01) and a positive main effect on mental and overall health (both $p$<0.05). Also, additive and incongruence effects of pain and physical activity were found on depression severity, mental, physical, and overall health were found (all $p$<0.05).

**Conclusion:**
We found that pain does not only lead to low mental health and depression symptoms in lipedema patients but also that pain can interfere with the potential of drawing benefit from engaging in physical activity. According to the current state-of-the-art, most lipedema patients receive a conservative therapy, e.g., including compression garment, manual lymphatic drainage, and intermittent pneumatic compression. However, most of the outcomes are of limited long-term effect [3]. Therefore, solutions leading to long-term reduced impairment and pain are of high relevance. In this context, liposuction can lead to substantial symptom reduction (e.g., pain relief) and might pave the way to benefit from engaging in physical activity [10], [11]. In sum, both multimodal strategies in pain treatment and liposuction are of high interest to reduce pain and thus to be able to benefit from the positive effects of physical activity.

[1] I. Forner-Cordero, G. Szolnoky, A. Forner-Cordero, and L. Kemény, “Lipedema: an overview of its clinical manifestations, diagnosis and treatment of the disproportional fatty deposition syndrome–systematic review,” *Clin. Obes.*, vol. 2, no. 3–4, pp. 86–95, 2012.
[2] D. W. Buck and K. L. Herbst, “Lipedema: a relatively common disease with extremely common misconceptions,” *Plast. Reconstr. Surg. Glob. Open*, vol. 4, no. 9, 2016.
[3] K. Peprah and D. MacDougall, “Liposuction for the treatment of lipedema: a review of clinical effectiveness and guidelines,” 2019.
[4] M. Hamatschek *et al.*, “Characteristics and Patient Reported Outcome Measures in Lipedema Patients—Establishing a Baseline for Treatment Evaluation in a High-Volume Center,” *J. Clin. Med.*, vol. 11, no. 10, p. 2836, 2022.
[5] C. Clarke, J. N. Kirby, T. Smidt, and T. Best, “Stages of lipoedema: experiences of physical and mental health and health care,” *Qual. Life Res.*, pp. 1–11, 2022.
[6] D. M. Landers and S. M. Arent, “Physical activity and mental health.,” 2007.
[7] K. Kroenke and R. L. Spitzer, “The PHQ-9: a new depression diagnostic and severity measure,” *Psychiatric annals*, vol. 32, no. 9. SLACK Incorporated Thorofare, NJ, pp. 509–515, 2002.
[8] WHOQOL Group, “The World Health Organization quality of life assessment (WHOQOL): position paper from the World Health Organization,” *Soc. Sci. Med.*, vol. 41, no. 10, pp. 1403–1409, 1995.
[9] L. R. Shanock, B. E. Baran, W. A. Gentry, S. C. Pattison, and E. D. Heggestad, “Polynomial regression with response surface analysis: A powerful approach for examining moderation and overcoming limitations of difference scores,” *J. Bus. Psychol.*, vol. 25, no. 4, pp. 543–554, 2010.
[10] S. Rapprich, A. Dingler, and M. Podda, “Liposuction is an effective treatment for lipedema–results of a study with 25 patients,” *JDDG J. Dtsch. Dermatol. Ges.*, vol. 9, no. 1, pp. 33–40, 2011.
[11] A. Baumgartner, M. Hueppe, I. Meier-Vollrath, and W. Schmeller, “Improvements in patients with lipedema 4, 8 and 12 years after liposuction,” *Phlebol. J. Venous Dis.*, vol. 36, no. 2, pp. 152–159, Mar. 2021, doi: 10.1177/0268355520949775.

Abstracts – DCK 2024 – 141st Congress of the German Society of Surgery – Leipzig, April 24–26 • DOI 10.1515/iss-2024-2008
Innov Surg Sci 2024; 9 (Special Suppl 1): s303–s385

**Isolation of primary human hepatocytes from from surgical liver specimens**
ID: 158

**SCF - Varia**

Laura Feiner[1], Matthias Glanemann[1], Claudia Rubie[1]

[1]*Universitätsklinikum des Saarlandes, Klinik für Allgemein-, Viszeral-, Gefäß- und Kinderchirurgie, Homburg/Saar, Deutschland*

**Background:**
The isolation of primary human hepatocytes (PHH) is an important tool to investigate liver cancer with special focus on the tipping point from various non cancerogeneous liver dieseases like non alcoholic fatty liver disease (NAFLD), cirrhosis and non alcoholic steatohepatitis (NASH) to hepatocellular carcinoma (HCC).

**Materials and methods:**
An established method ist the two-step EGTA/collagenase perfusion technique. However, this method is only applicable if the tissues are at least 10 g in weight and have an intact capsule. Further, they should have a single, small cutting surface and visible vessels. However, due to new surgery techniques surgery specimens received from the patients often do not match these criteria. Therefore, we present a new method that we adjusted to our requirements.

**Results:**
This method is based on the application of the GentleMACS™ Octo Dissociator with Heaters (Miltenyi Biotec) which is suitable for small liver pieces (>1 g) and easy in handling. However, only one kit is avaiable for human liver, the human tumor dissociation kit, which is established only for tumor tissues. We established a protocol for the isolation of hepatocytes and non parenchymal cells from healthy liver tissues.

**Conclusion:**
In summary, we present a new method for the isolation of PHH from resected liver tissue samples from HCC patients and colorectal liver metastases comparing advantages and disadvantages for the application of this technique.

**Association of patient characteristics on bacterial composition in Surgical Site Infections: Insights from a National Surveillance Study**
ID: 425

**SCF - Varia**

Sarah Peisl[1], Hugo Armando Guillen-Ramirez[1], Daniel Sánchez-Taltavull[1], Andreas Widmer[2 3], Rami Sommerstein[2 4 5], Guido Beldi[1]

[1]*Department of Visceral Surgery and Medicine, Inselspital, Bern University Hospital, University of Bern, Bern, Schweiz*, [2]*National Centre for Infection Control, Swissnoso, Bern, Schweiz*, [3]*Division of Infectious Diseases and Hospital Epidemiology, University of Basel Hospitals and Clinics, Basel, Schweiz*, [4]*Department of Infectious Diseases, Inselspital, Bern University Hospital, University of Bern, Bern, Schweiz*, [5]*Faculty of Health Sciences and Medicine, University of Lucerne, Luzern, Schweiz*

**Background:**
The type of surgical procedure determines microbial composition of surgical site infections (SSI). However, it remains unknown to what extent patient-dependent (surgery-independent) factors contribute to the microbial composition of SSI. The objective of this work was to identify patient-dependent predictors for the microbial composition in SSI across different types of surgery.

**Materials and methods:**
This retrospective cohort study analyzed data extracted from a national surgical surveillance program, encompassing patients who underwent surgery between January 2009 and December 2020. To identify predictors of microbial composition in SSI, patients presenting a SSI with available microbiology results were selected for machine learning analyses. Germs identified in SSI as well as 20 relevant clinical features (e.g., sex, age, BMI, duration of surgery, type of surgical procedure, surgical antimicrobial prophylaxis) were included for the analysis. The data was divided into an 80% training set and a 20% independent test set. Subsequently, multilabel classifiers Adaptive Boosting and Gaussian Naive Bayes were used to identify key predictors.

**Results:**
Data from 538,893 patients undergoing visceral (39.5%), orthopedic (37.1%), gynecological (15.9%), and heart surgery (7.3%) were included. Overall, SSI were recorded in 3.8% of the patients (N = 18,642) among which microbiology from wound swabs were available in 10,632 patients. Most common pathogens identified in SSI were Enterobacterales (57%), *Staphylococcus* spp. (31%) and *Enterococcus* spp. (28%). Patient dependent factors age (mean feature importance of 0.27 (95%CI 0.22, 0.32), BMI (0.231 (95%CI 0.180, 0.280)) and duration of surgery (0.230 (95%CI 0.183, 0.283)) were strong and independent predictors for the microbial composition of SSI, followed by the type of surgical procedure (0.07, 95%CI: 0.05, 0.09). On partial dependence plots, increasing age and duration of surgical procedure were shown to be associated with a shift from *Staphylococcus* spp. to Enterobacterales and *Enterococcus* spp. in SSI.

**Conclusion:**
Age, BMI, and duration of surgery influence the microbial composition of SSI, implying the relevance of host factors, independently of the surgical procedure. This signifies that the origin of SSI's development extends beyond the mere spillage of microbes from the surgical site or surroundings. It underscores the pivotal influence of host factors in driving SSI, potentially through compromised segregation of the intestinal microbiota. Such knowledge could facilitate the development of targeted preventive measures and personalized treatment strategies to mitigate the occurrence and impact of surgical site infections

**NLRP3 inflammasome activation in neutrophils promotes hepatic stellate cell activation in murine non-alcoholic steatohepatitis**
ID: 430

**SCF - Varia**

Benedikt Kaufmann[1], Laela M. Booshehri[2], Aleksandra Leszczynska[2], Helmut Friess[1], Daniel Hartmann[1], Lori Broderick[2], Hal M. Hoffman[2], Ariel Feldstein[2]

[1]*Klinikum rechts der Isar der Technischen Universität München, München, Deutschland*, [2]*University of California, La Jolla, United States*

**Background:**
Chronic inflammation represents a central driver of disease progression in Nonalcoholic Steatohepatitis (NASH). Activation of the NLRP3 inflammasome, a cytosolic multiprotein complex that mediates sterile inflammatory responses, in leukocytes has been proposed to be a key driver of murine and human NASH. However, the relevance of NLRP3 inflammasome activation in either macrophages or neutrophils that contribute to the pathology of NASH remains unknown. In this study the contribution of neutrophil specific NLRP3 inflammasome activation in NASH was investigated.

**Materials and methods:**
Neutrophil specific Nlrp3 knock-out mice were generated to investigate the role of NLRP3 inflammasome activation in neutrophils. A chronic (choline-deficient, L-amino acid-defined high-fat diet (CDAA-HFAT)) liver injury model was used to induce in vivo NLRP3 activation and fibrotic-non-alcoholic steatohepatitis (NASH). Mice were put on CDAA-HFAT diet for 10 weeks and liver steatosis, inflammation and fibrosis were analysed by immunohistochemistry and RT-PCR.

**Results:**
compared to WT mice. Interestingly, neutrophil-specific Nlrp3 knock-out resulted in reduced mRNA levels of α-SMA and vimentin, markers for HSC activation, compared to WT mice analysed by RT-PCR. Furthermore, α-SMA expression was decreased on protein level in neutrophil-specific Nlrp3 knock-out mice compared to WT mice analysed by immunohistochemistry. Liver fibrosis, a key feature of NASH, was reduced in neutrophil-specific Nlrp3 knock-out mice compared to WT mice. Expression of mRNA of Col3a and CTGF were reduced in neutrophil-specific Nlrp3 knock-out mice compared to WT mice.

**Conclusion:**
This study provides novel insights in the cell-specific role of NLRP3 activation in neutrophils in the pathogenesis of NASH. NLRP3 inflammasome activation in neutrophils was identified as crucial for the progression to fibrotic-NASH.

Abstracts – DCK 2024 – 141st Congress of the German Society of Surgery – Leipzig, April 24–26 • DOI 10.1515/iss-2024-2008
Innov Surg Sci 2024; 9 (Special Suppl 1): s303–s385

**Adjusting the RAPID score with 2 additional variables significantly increases its predictive value in patients with empyema**
ID: 442

**SCF - Varia**

Gabriel Plitzko[1], Björn-Ole Stüben[1], Franka Urban[1], Marius Kemper[1], Jonas Wakker[1], Jakob Izbicki[1], Kai Bachmann[1]

[1]*Klinik für Allgemein-, Viszeral- und Thoraxchirurgie, Universitätsklinikum Hamburg-Eppendorf, Hamburg, Deutschland*

**Background:**
Pleural empyema is a serious condition leading to a significant burden on health care systems. Treatment ranges from non-surgical interventions (antibiotic therapy, chest tube placement) to thoracoscopic or open surgery. The RAPID (renal, age, purulence, infection source, and dietary factors) score is a clinical risk score which identifies patients at risk of death and may be used to formulate individual treatment strategies accordingly. The aim of this study was to identify perioperative risk factors which significantly impact treatment outcomes but are currently not included in the RAPID score.

**Materials and methods:**
245 patients with pleural empyema surgically treated at the University Medical Centre, Hamburg, Germany, were retrospectively analysed. All patients which received either minimally invasive or open thoracic surgery were included. Epidemiological and perioperative data was analysed to identify risk factors which impact long-term overall outcomes.

**Results:**
The mean age was 59.4 years. There was a male predominance across the cohort (71.4% compared to 28.6%), with no significant differences across ages below or above 60 years. 53 (21.6%) patients died within the first 90 days. Diabetes type 1 and 2, renal replacement therapy, immunosuppression, postoperative bleeding, intraoperative transfusion as well as microbiologically confirmed bacterial invasion of the pleura all led to higher mortality rates. Higher RAPID scores accurately predicted higher 90-day mortality rates. Modifying the RAPID score by adding the comorbidities diabetes and renal replacement therapy significantly increased the predictive value of the score.

**Conclusion:**
Various perioperative and patient related risk-factors not included in the RAPID score negatively impact postoperative outcome in patients receiving surgical treatment for pleural empyema. These should be taken into consideration when deciding on the best course of treatment. If confirmed in a prospective study including non-surgical patients with a significantly larger cohort, it may be worth considering expanding the RAPID score to include these.

**Exploring MAFLD Progression through the Synergy of Patient-Derived Organoids and Computational Modeling**
ID: 521

**SCF - Varia**

Robert Frédéric Pohlberger[1 2], Thomas Stiehl[3], Thorsten Cramer[1 4 5], Steven Olde Damink[4 5], Sander Rensen[4 5], Anjali A. Roeth[1 4]

[1]*Klinik für Allgemein-, Viszeral- und Transplantationschirurgie, Universitätsklinikum RWTH Aachen, Aachen, Deutschland*, [2]*IZKF-Interdisziplinäres Zentrum für Klinische Forschung, Universitätsklinikum RWTH Aachen, Aachen, Deutschland*, [3]*Institute for Computational Biomedicine and Disease modelling, Universitätsklinikum RWTH Aachen, Aachen, Deutschland*, [4]*Department of Surgery, Maastricht University Medical Center, Maastricht, Netherlands*, [5]*NUTRIM - School of Nutrition and Translational Research in Metabolism, Maastricht University Medical Center, Maastricht, Netherlands*

**Background:**
Fatty liver is a common disease within society, affecting approximately 25 % of the world's population. Here, special attention will be paid to MAFLD and MASH. This subordinate form of fatty liver disease results in lipotoxicity and inflammatory damage to hepatocytes. Utilizing cutting-edge patient-derived organoids and advanced computational modeling, our objective is to establish a mechanical framework capable of elucidating and forecasting the underlying metabolic shifts throughout the progression of MAFLD.

**Materials and methods:**
Patient-derived organoids were cultured by established methods. The tissue samples were collected after informed consent by patients. Tissue was digested, and the resulting cells were cultured in a 3D-extracellular matrix. Organoids were cultured for at least one week. Afterwards, the growth kinetics and shape of the organoids were monitored. Patients were divided into two groups: healthy and fatty liver. A computational model was developed focusing on proliferation, differentiation, and cell death. These models were instrumental in generating hypotheses regarding the interplay of critical metabolic pathways in fatty liver disease stages. Data, e.g., proliferation, apoptosis, or cell-cell contact, used in the computational model was taken from immunohistochemistry.
Both groups were compared to differences in the progress of a fatty liver.

**Results:**
Organoids from both groups, healthy and fatty liver, were successfully cultured. Organoids from patients with different gender, ages, and disease histories (healthy or fatty liver) showed different growth kinetics and shapes. We successfully incorporated experimental data, growth kinetics, and immunohistochemistry into computational models for a robust fit.
This integration provided valuable insights into disease progression, enriching our comprehension of its dynamics.

**Conclusion:**
In summary, our study leveraged patient-derived organoids and cutting-edge computational modeling to assess the growth kinetics of NAFLD organoids, revealing individualized growth patterns and morphological changes over time. This collaborative synergy between experimental and computational methods demonstrates promising prospects for advancing organoid research.

**Der Verlust des Sphingosin-1-Phosphat-Rezeptors S1PR4 führt zu deutlich erhöhter Sterblichkeit in einem murinen Modell der peritonealen Sepsis**
ID: 584

**SCF - Varia**

Maurice Hannemann[1], Andreas Felix Lührs[1], Janik Riese[1], Jonas Menz[1], Annabel Kleinwort[1], Stephan Kersting[1], Tobias Schulze[1]

[1]*Universitätsmedizin Greifswald, Klinik und Poliklinik für Allgemeine Chirurgie, Viszeral-, Thorax- und Gefäßchirurgie; Chirurgisches Forschungslabor, Greifswald, Deutschland*

**Background:**
Die Sepsis ist eine lebensbedrohliche Erkrankung, bei der es aufgrund einer dysregulierten Immunantwort auf eine Infektion zu einer Organdysfunktion kommt (1). In Deutschland erkranken jährlich mindestens 230.000 Patienten an einer Sepsis, mindestens 85.000 davon versterben (2). Nach einer pulmonalen Ursache stellt dabei die intraabdominelle Infektion mit 23% den zweithäufigsten Infektfokus dar (3). Die peritoneale Sepsis bildet einen der Hauptfaktoren für Morbidität und Mortalität in der abdominellen Chirurgie (4).
Obwohl die zell- und molekularbiologischen Mechanismen der Sepsis sehr intensiv untersucht wurden, verbleiben aktuell noch wesentliche Lücken in unseren Kenntnissen über die Ursachen des dysregulierten Gleichgewichts zwischen proinflammatorischen und antiinflammatorischen Vorgängen sowohl in der frühen als auch in den späteren Phasen der Sepsis (5).
Das phylogenetisch hoch konservierte Lysophospholipid Sphingosin-1-Phosphat (S1P) dient im Körper sowohl als Membranbestandteil als auch als intra- und extrazellulärer Botenstoff. Die Wirkung als extrazellulärer Botenstoff wird über fünf differential exprimierte Membranrezeptoren (S1PR) vermittelt. S1PR vermitteln vielfältige Wirkungen auf Zellen des angeborenen und adaptiven Immunsystems, des Gerinnungssystems und des Endothels (6-8). Der S1P-Rezeptor $S1PR_4$ beeinflusst Migrationsprozesse und Zytokinsekretion von Immunzellen und ist damit auch in die Steuerung von Prozessen involviert, die in der Entwicklung einer Sepsis eine wichtige Rolle spielen (8-10). Obwohl gezeigt werden konnte, dass S1P-Spiegel im Blut eine prognostische Bedeutung in der Sepsis haben, wurde die Rolle von $S1PR_4$ in der abdominellen Sepsis bisher nicht untersucht (11).

**Materials and methods:**
Als Sepsismodell wurde das etablierte Colon Ascendens Stent Peritonitis (CASP) Modell einer polymikrobiellen abdominellen Sepsis genutzt (12). Mithilfe eines $S1PR_4$-defizienten Mausstammes auf dem C57BL/6J Hintergrund ($s1pr_4^{-/-}$) erfolgte die vergleichende Analyse mit Wildtyp-Tieren (*wt*) des gleichen genetischen Hintergrundes im CASP-Modell. Die Untersuchung der Immunzellpopulationen der Peritonealhöhle, des Knochenmarks, der Milz und der mesenterialen Lymphknoten erfolgte nach 6h, 24h und 7 Tagen mittels FACS. Die Bestimmung des Zytokinprofils im Plasma und der Peritoneallavage erfolgte zu den gleichen Zeitpunkten mittels durchflusszytometrischem Multiplex-Immunoassay (biolegend LegendPlex™). Die Organschädigung wurde mittels Bestimmung von ASAT und ALAT im Plasma erfasst. Die Bestimmung der Bakterienlast in Leber, Lunge, Niere, Blut und Peritoneallavage nach 24h erfolgte quantitativ nach 24-stündiger Kultur auf Columbia Blutagar. Schließlich erfolgte die Bestimmung einer Überlebenskinetik über 7 Tage.

**Results:**
$s1pr_4^{-/-}$ Tiere zeigte ein signifikant verringertes Überleben nach Sepsisinduktion mittels CASP (7-Tagesüberleben: $s1pr_4^{-/-}$: 55%, *wt*: 83%, p = 0,03). Die Marker für Organschäden zeigten im frühen Verlauf eine stärkere Erhöhung in $s1pr_4^{-/-}$ Tieren. Die korpuskuläre Gerinnung war in der frühen Phase der Sepsis in $s1pr_4^{-/-}$ Tieren deutlich stärker eingeschränkt. Es bestanden deutliche Unterschiede in der Zusammensetzung der peritonealen Immunzellpopulationen zwischen $s1pr_4^{-/-}$ und *wt* Tieren betreffend die peritonealen B-Zell-, Granulozyten und Makrophagenpopulationen. Die Bakterienlast im Blut, in der

Leber und der Niere war nach 24h in *s1pr4*$^{-/-}$ Tieren im Vergleich zu *wt* Tieren signifikant erhöht. Die Zytokinprofile in frühen Stadien der peritonealen Sepsis zeigten deutliche Unterschiede (signifikant erhöht nach 6h in *s1pr4*$^{-/-}$ Tieren im Vergleich zu *wt* Tieren: IL-1α, IL-3, TNF-α, IL-33, IL-10, IL-17A und IL-6; signifikant erniedrigt nach 6h in *s1pr4*$^{-/-}$ Tieren: TGF-β1).

**Conclusion:**
Die vorliegenden Ergebnisse legen eine protektive Rolle von $S1PR_4$ in der abdominellen Sepsis nahe. Das Fehlen dieses Rezeptors resultierte in einer deutlich erhöhten Mortalität nach CASP. Die signifikant erhöhten Erregerzahlen im Blut und in den parenchymatösen Organen in *s1pr4*$^{-/-}$ Tieren weisen auf eine kompromittierte Immunantwort gegen das peritoneale bakterielle Inokulum hin. Weitere Untersuchungen sind notwendig, um einen funktionellen Zusammenhang zwischen den verminderten Zahlen peritonealer Immunzellen und der erhöhten Mortalität der *s1pr4*$^{-/-}$ Tiere im CASP-Modell eindeutig nachzuweisen und andere potentielle Mechanismen, z.B. eine endotheliale Schrankenstörung, auszuschließen. Unsere Ergebnisse weisen auf eine potentielle Rolle von $S1PR_4$ als pharmakologisches Target in der abdominellen Sepsis hin.

Literatur:

1. Singer M, Deutschman CS, Seymour CW, Shankar-Hari M, Annane D, Bauer M, Bellomo R, Bernard GR, Chiche J-D, Coopersmith CM, Hotchkiss RS, Levy MM, Marshall JC, Martin GS, Opal SM, Rubenfeld GD, van der Poll T, Vincent J-L, Angus DC. The Third International Consensus Definitions for Sepsis and Septic Shock (Sepsis-3). JAMA. 2016;315(8):801-810.
2. IQTIG (Hrsg.) Entwicklung eines Qualitätssicherungsverfahrens „Diagnostik, Therapie und Nachsorge der Sepsis“. Abschlussbericht, Berlin 2022
3. Stearns-Kurosawa DJ, Osuchowski MF, Valentine C, Kurosawa S, Remick DG. The pathogenesis of sepsis. Annu Rev Pathol 2011;6:19-48.
4. S. Maier, T. Traeger, A. Westerholt, Prof. Dr. C.-D. Heidecke. Besonderheiten der abdominellen Sepsis. Die Chirurgie 2005;9
5. Hotchkiss RS, Monneret G, Payen D. Sepsis-induced immunosuppression: from cellular dysfunctions to immunotherapy. Nat Rev Immunol2013;13:862-874.
6. Matloubian M, Lo CG, Cinamon G, Lesneski MJ, Xu Y, Brinkmann V, et al. Lymphocyte egress from thymus and peripheral lymphoid organs is dependent on S1P receptor 1. Nature 2004;427:355-360.
7. Dorsam G, Graeler MH, Seroogy C, Kong Y, Voice JK, Goetzl EJ. Transduction of multiple effects of sphingosine 1-phosphate (S1P) on T cell functions by the S1P1 G protein-coupled receptor. J Immunol 2003;171:3500-3507.
8. Schulze T, Golfier S, Tabeling C, Rabel K, GralerMH, Witzenrath M, et al. Sphingosine-1-phospate receptor 4 (S1P4) deficiency profoundly affects dendritic cell function and TH17-cell differentiation in a murine model. Faseb J 2011;25:4024-4036.
9. Müller J, v. Bernstorff W, Heidecke C-D, Schulze T. Differential S1P receptor profiles on M1 and M2 polarized Macrophages affect macrophage cytokine production and migration. BioMed Research International 2017:in press.
10. Golfier S, Kondo S, Schulze T, Takeuchi T, Vassileva G, Achtman AH, et al. Shaping of terminal megakaryocyte differentiation and proplatelet development by sphingosine-1-phosphate receptor S1P4. Faseb 2010;24:4701-4710.
11. Winkler MS, Nierhaus A, Holzmann M, Mudersbach E, Bauer A, Robbe L, Zahrte C, Geffken M, Peine S, Schwedhelm E, Daum G, Kluge S, Zoellner C. Decreased serum concentrations of sphingosine-1-phosphate in sepsis. Crit Care 2015; 19(372): 015-1089.
12. Maier S, Traeger T, Entleutner M, Westerholt A, Kleist B, Huser N, et al. Cecal ligation and puncture versus colon ascendens stent peritonitis: two distinct animal models for polymicrobial sepsis. Shock. 2004;21:505-511.

Abstracts – DCK 2024 – 141st Congress of the German Society of Surgery – Leipzig, April 24–26 • DOI 10.1515/iss-2024-2008
Innov Surg Sci 2024; 9 (Special Suppl 1): s303–s385

**Implementation of individual name tagging in the operation room can improve interprofessional communication**
ID: 844

**SCF - Varia**

Alexander Bungert[1], Jan Philipp Ramspott[1], Alina Knipping[1], Carsten Szardenings[2], Andreas Pascher[1]

[1]*Universitätsklinikum Münster (UKM), Klinik für Allgemein-, Viszeral- und Transplantationschirurgie, Münster, Deutschland*, [2]*Universitätsklinikum Münster (UKM), Institute of Biostatistics and Clinical Research, Münster, Deutschland*

**Background:**
Effective and reliable communication is the cornerstone of safe communication in the operating room (OR). The OR is one of the most dynamic places in the hospital where multiple disciplines (surgeons, nurses, anaesthesiologists and service staff) have to work together in perfect harmony to ultimately improve patients' outcomes. To create familiarity by name regarding constantly changing team members, individual name tagging was implemented in the OR. Our study evaluates the project in terms of employees' overall ratings, compliance, implementation, and influence on interprofessional communication.

**Materials and methods:**
We retrospectively analysed the impact of name tagging in the OR. Name tags (either first or last name), coloured according to the specific department, have been put on the sterile bonnet since March 2023. Two months after implementation, a total of 440 anaesthesiologists, general, visceral and trauma surgeons, nurses, and service staff were invited to answer an evaluation questionary of nine questions.
101 people answered the query which, amongst others, asked for overall ratings, compliance, evaluation of specific items as well as positive and negative aspects which were stated in free text. Statistical analyses were done by a statistician using R.

**Results:**
Most of the interviewed staff (median=4; scale 1-5, 1=negative, 5=positive) rated the implementation of name tagging positive. Greatest benefit was seen in items such as communication in general, direct contact to colleagues and delegation of tasks. Most of the staff (>90 %) was adherent to the new project and used it regularly. Negative aspects like potential loss of sterility due to material defects, loss of respectability, and environmental impact were announced at the same time. Potential for improvement was seen in the bonding method of attachment or in the implementation. Here, location of the name tag or size was mentioned for instance.

**Conclusion:**
All in all, Individual name tagging in the OR can improve interprofessional communication. Besides the well-established World Health Organization (WHO) Surgical Safety Checklist or the Crisis Resource Management (CRM), individual name tagging is one tool to enhance patient safety due to decreasing reservations or intimidations towards hitherto unknown colleagues. More studies are required to determine long term effects on patient safety, outcome or employees' satisfaction.
